# Supplementary material for: Developing a competency assessment framework for medical laboratory technologists in primary healthcare settings in India
Source: PLoS One. 2024 Apr 1;19(4):e0294939. doi: 10.1371/journal.pone.0294939 (PMC10984544; doi:10.1371/journal.pone.0294939)
Supplement: S1 File — (PDF) [file pone.0294939.s001.pdf]

S1 Supporting Information- Literature Based Medical Laboratory Technologist's Competencies

Category	Sr. No.	Competencies
**Safe Work Practices**	1	Able to apply the principles of routine practices
	2	Able to use personal protective equipment appropriately, e.g., gloves, gowns, mask, face shields, aprons
	3	Able to apply laboratory hygiene and infection control practices
	4	Able to minimize possible dangers from biological specimens, laboratory supplies and equipment
	5	Able to use laboratory safety devices, e.g., biological safety cabinets, fume hoods, laminar flow cabinets, safety pipetting devices, safety containers and carriers, safety showers, eye washes
	6	Able to label, dates, handles, stores, and disposes chemicals, dyes, reagents, and solutions according to legislation, e.g., WHMIS
	7	Able to handle and disposes sharps (BMW Policy)
	8	Able to store, handle, transport and disposes biological and other hazardous materials according to BMW Policy
	9	Able to use disinfection and sterilization method to disinfect materials used
	10	Able to minimize potential hazards related to disinfection/sterilization methods
	11	Able to apply measures in response to laboratory accidents/incidents
	12	Able to apply spill containment and clean up procedures for biological and other hazardous materials
	13	Able to ensure the availability of adequate availability of medical and diagnostic supplies
	14	Able to document all incidents related to safety and personal injury
**Data/Sample Collection**	15	Able to verify relevant information provided for test request
	16	Able to assist in fine needle aspiration-Swap the skin with antiseptic solution; prepare the needle of very fine diameter for the process; take and record the vitals.
	17	Able to provide information to the client on specimen collection, transportation, and storage
	18	Able to confirm the identity of the patient and performs venipuncture and capillary blood collection to obtain appropriate samples for laboratory analysis
	19	Able to perform sample collection and chain of custody procedures relating to specimens with legal implications
	20	Able to adhere to established protocols for labelling and traceability of specimens
**Specimen Preparation**	21	Able to delivers specimens considering priority and stability
	22	Able to assess suitability of the specimen for testing
	23	Able to verify that the pertinent data on the specimen and requisition correspond
	24	Able to access specimens into laboratory information systems
	25	Able to adhere to guidelines for specimen retention, storage, transportation, and disposal

S1 Supporting Information- Literature Based Medical Laboratory Technologist's Competencies

	26	Able to prepare specimens for analysis
	27	Able to identify, documents and initiates corrective action for pre-examination (pre- analytical) errors
	28	Able to prepare blood, body fluids and other clinical specimens for microscopic examination
**Equipment Instrument and Regiments**	29	Able to prepare reagents, calibrators, standards, and quality control materials
	30	Able to perform point-of-care testing and assess results
**Assessment and Analysis**	31	Able to apply for the principles of microscopy: bright field; fluorescence; polarizing; inverted
	32	Able to apply the physical and chemical principles of staining & the quality of staining and initiates corrective action
	33	Able to apply the principles of light measuring systems used in common instruments: absorption spectrophotometry; reflectometry; turbidimetry
	34	Able to assess results, identifies sources of interference and initiates corrective action
	35	Able to apply principles of electrophoresis and chromatography
	36	Able to apply principles of immunoassays
	37	Able to demonstrate knowledge of principles of mass spectrometry
	38	Able to apply principles of particle analysis used in common haematology instrumentation
	39	Able to perform manual counting procedures
	40	Able to demonstrate the knowledge of principles of flow cytometry
	41	Able to apply the principles of haemostasis to perform coagulation testing
	42	Able to analyse to detect and identify common clinically significant micro-organisms Selects appropriate culture media and environment for isolation
	43	Able to Identify and evaluates the morphology of cellular and non-cellular elements in microscopic preparations
	44	Able to differentiates between clinically significant and insignificant findings
	45	Able to perform compatibility analyses
**Recording and Reporting**	46	Able to recognizes the relationship between analyses, diagnoses, clinical information, and treatment by assessing results based on specimen integrity; reference values; critical values; method limitations, e.g., dynamic ranges, interferences, specificity, sensitivity; patient delta checks; clinical conditions; other laboratory findings
	47	Able to report results that meet quality control criteria
	48	Able to Identify unexpected or implausible results and takes appropriate action prior to reporting
	49	Able to recognize and acts on critical values

S1 Supporting Information- Literature Based Medical Laboratory Technologist's Competencies

	50	Able to document results accurately
**Infection control**	51	Able to perform the standards precautions to prevent the spread of infection in accordance with Organization requirements
	52	Able to minimize contamination of materials, equipment, and instruments by aerosol and splatter
	53	Able to follow protocols for care following exposure to blood or other body fluids as required
	54	Able to place appropriate signs when and where required
	55	Able to maintain hand hygiene by washing hands before and after patient contact and /or after any activity likely to cause contamination
	56	Able to cover cuts and abrasions with waterproof dressings and changes as necessary
	57	Able to demarcate and maintain clean and contaminated zones in all health care work
	58	Able to abide BMW guidelines
**Quality Management**	59	Able to follow established protocols as defined in policy, process and procedure manuals
	60	Able to use simple statistics to monitor and track the acceptability of quality control results Identifies, documents and reports deficiencies that may affect the quality of testing
	61	Able to perform and document preventative maintenance according to established protocols
	62	Able to recognize malfunctions in equipment/instruments, initiates and documents for corrective action
	63	Able to participate in internal and external quality assurance activities, e.g., proficiency testing, audits, accreditation
	64	Able to demonstrate knowledge of inventory maintenance
	65	Able to demonstrates information management skills, e.g., computer, laboratory information systems and related technology
	66	Able to identify and correct any hazards that he/she can deal with safely, competently, and within the limits of his /her authority
	67	Able to report promptly and accurately any hazards that he/she is not allowed to deal with to the relevant person and warn other people who may be affected
	68	Able to Inspect equipment, structure, or materials to identify the cause of errors or other problems or defects
	69	Able to complete any health and safety records legibly and accurately
	70	Able to report any identified breaches in health safety, and security procedures to the designated person
	71	Able to identify and recommend opportunities for improving health safety and security to the designated person
**Critical Thinking**	72	Able to engage in reflective practice; stops and thinks about practice, consciously analyses decision making and draws conclusions to improve future practice
	73	Able to Organize work to accommodate priorities

S1 Supporting Information- Literature Based Medical Laboratory Technologist's Competencies

	74	Able to ensure efficient use of resources, e.g., time, equipment,
	75	Able to demonstrate effective problem solving/trouble-shooting strategies and initiates appropriate follow up
**Communication and Interactions**	76	Able to communicate effectively with colleagues, patients/clients and other health care professionals:<br>**A.** Active listening; **B.** Verbal communication; **C.** Non-verbal communication; **D.** Written communication; **E.** Conflict management; **F.** Identifying barriers to effective communication; G. Using technology appropriately to facilitate communication
	77	Able to demonstrate effective teamwork skills
	78	Able to demonstrates interdisciplinary/interprofessional team skills:<br>**A.** Communication; **B.** Collaboration; **C.** Role clarification; **D.** Reflection
	79	Able to demonstrates adaptive skills when interacting with patients
**Professional Practice**	80	Able to maintain confidentiality of healthcare information
	81	Able to Comply with legislations that govern medical laboratory technology
	82	Able to Recognize limitations of own competence and seeks action to resolve
	83	Able to obtain informed consent prior to procedure and respects a patient's right to refuse
	84	Able to identify the need for and participates in continuing education and training
	85	Respects the diversity, dignity, values, and beliefs of patients/clients and colleagues
	86	Able to demonstrate knowledge of interpersonal skills: A. Recognizes signs of individual and group stress; B. Recognizes signs of patient stress; C. Exhibits empathy when assisting patients and colleagues
