# Supplementary material for: Developing a competency assessment framework for medical laboratory technologists in primary healthcare settings in India
Source: PLoS One. 2024 Apr 1;19(4):e0294939. doi: 10.1371/journal.pone.0294939 (PMC10984544; doi:10.1371/journal.pone.0294939)
Supplement: S2 File — (PDF) [file pone.0294939.s002.pdf]

Competencies for Medical Laboratory Technicians in Primary Health Care Setting

Domain	Sr. No.	Competencies
**Human Values and Professional Ethics**	1)	Able to understand the human values and ethics in a clinical laboratory
	2)	Able to maintain the confidentiality of healthcare information
	3)	Able to demonstrate their responsibilities towards patients, their attendants (Public), regulatory bodies, the profession, and towards himself/herself as expected from Medical Laboratory Professionals
	4)	Respects the diversity, dignity, values, and beliefs of patients/clients and colleagues
	5)	Able to contribute towards continual improvement in the professional services focussing on essential aspects like quality of work
	6)	Able to comply with legislation that governs medical laboratory technology at local, state-level, or national levels as applicable
	7)	Able to recognize limitations of own competency and seeks action to resolve
	8)	Able to obtain informed consent before the procedure wherever required and respect a patient's right to refuse
	9)	Able to develop his/ her interest to participate in continuing education and training – Interest to learn and improve the quality of the work environment
**Quality Management**	10)	Able to understand and demonstrate the concepts of quality management system including Quality Control, Quality Assurance, Quality improvement, consistency, reproducibility, Turn Around Time etc,
	11)	Able to understand Corrective actions, Root cause analysis, and Preventive actions in case of any incidents/accidents and observable quality degradation during pre-analytical, analytical and post analytical phase of testing.
	12)	Able to follow established protocols as defined in the quality policy, process, and procedure manuals
	13)	Able to use simple mathematics to monitor and track the acceptability of quality control results. Identifies, documents, and reports deficiencies that may affect the testing quality and the

		generated test report.
	14)	Able to understand and perform preventive maintenance and calibration of Laboratory instruments according to established protocols
	15)	Able to participate in internal and external quality assurance activities, e.g., audits, accreditation
	16)	Able to demonstrate knowledge of inventory control by adopting FIFO (First in and First Out)
**Communication Skills**	17)	Able to communicate effectively with patients/clients, colleagues, and other health care professionals in local or regional language with respect to:<br>A. Active listening<br>B. Verbal communication<br>C. Non-verbal communication<br>D. Written communication<br>E. Conflict management<br>F. Identifying barriers to effective communication<br>G. Using technology appropriately to facilitate communication
	18)	Able to understand medical terminology used at least related to the functioning of Medical Laboratories
	19)	Able to understand and use the abbreviations commonly used in medical terminology
	20)	Able to demonstrate effective interdisciplinary/intra professional team skills through:<br>A. Communication<br>B. Collaboration<br>C. Role clarification
	21)	Able to demonstrate adaptive skills when interacting with patients
**Critical Thinking**	22)	Able to engage in reflective practice; consciously analyses, makes decisions, and draws conclusions to improve future practice
	23)	Able to organize work to accommodate valid priorities
	24)	Able to ensure efficient use of 4 M (Money, Man, Material, and Minutes).

Competencies for Medical Laboratory Technicians in Primary Health Care Setting

	25)	Able to demonstrate effective problem solving/trouble-shooting strategies and initiates the appropriate follow-up
	26)	Able to think about higher education and its importance in improving service quality
**Equipment Instruments, and Consumables**	27)	Able to prepare reagents, calibrators, standards, and quality control materials
	28)	Able to demonstrate shelf life of ready to use commercially procured and in-house materials prepared materials and reagents
	29)	Able to check the functional quality of all reagents / diagnostic kits/cards/ chemicals used in the Laboratory
	30)	Able to use/demonstrate positive and negative controls and their importance in all tests
	31)	Able to demonstrate the work principles of all the laboratory instruments/equipment
	32)	Able to perform risk assessment while using any equipment
	33)	Able to have the concepts of calibration of equipment and its importance in laboratory services
	34)	Able to maintain equipment working and its integrity by its regular maintenance
	35)	Able to inform competent authorities if the equipment has gone out of order
	36)	Able to maintain maintenance record of all equipment from time to time
	37)	Able to demonstrate functional quality check of all the available equipment/instruments
	38)	Able to label the equipment/instrument with their respective unique I.D.s, date of purchase, date of installation, date of putting into service, date of the last calibration, and name and contact of address mechanic whom to inform in case of emergency
	39)	Able to prepare and demonstrate SOPs for the use of all equipment/instruments, test procedures, and their display on workbenches
**Test Requisitions Data and Sample**	40)	Able to understand relevant information provided for test request on requisition form mainly, which is essential for the interpretation of results or critical alerts or recording purposes
	41)	Able to confirm the identity of the patient and performs venepuncture and capillary blood collection to obtain appropriate samples for laboratory analysis
	42)	Able to provide all relevant information about precautions on specimen collection,

Competencies for Medical Laboratory Technicians in Primary Health Care Setting

**Collection**		transportation, and storage to the patients/clients wherever required.
	43)	Able to perform patient counselling /preparation for collection of clinical samples
	44)	Able to demonstrate the types and use of procedures or tools for sample collection like vacutainers for blood samples
	45)	Able to demonstrate what type of sample is to be collected for a required test like serum plasma or whole blood etc
	46)	Should know what types of anticoagulants are to be used for blood or other samples if required
	47)	Able to demonstrate skin disinfection procedures before taking any blood sample or otherwise wherever required
	48)	Able to perform the collection of samples for tests routinely done at PHCs and chain of custody procedures relating to specimens as per requirements of the test procedure
	49)	Able to pack the samples safely and transport clinical samples to higher competent laboratories for advance testing or any other purpose, keeping in view all sample and environmental safety requirements
**Specimen Preparation**	50)	Able to identify and process specimens considering priority. Able to verify that the pertinent data on the specimen and requisition correspond
	51)	Able to assess the suitability of the specimen for testing
	52)	Able to prepare specimens for analysis like; blood, body fluids, and other clinical specimens for any of examination including microscopic.
Assessment & Analysis	53)	Able to follow the test/equipment SOPs properly and understand their importance
	54)	Able to apply the physical and chemical principles of staining & the quality of staining and initiates corrective action
	55)	Able to apply the principles of routine microscopy
	56)	Able to apply the principles of light measuring systems used in common instruments: reflectometry; turbidimetry
	57)	Able to assess results, identifies sources of interference, and initiate corrective action

Competencies for Medical Laboratory Technicians in Primary Health Care Setting

	58)	Able to perform Card-based immunoassays tests and tests using commercially available ready to use kits for tests done at PHCs with knowledge of all the precautions affecting the test results
	59)	Able to apply principles of commonly used analyzers/ semi-auto analyzers for haematology and biochemistry if available at respective PHC.
	60)	Able to perform manual counting procedures using cell counters etc.
	61)	Able to apply the principles of haemostasis to perform coagulation testing
	62)	Able to perform some common biochemistry tests using manual methods
	63)	Able to Identify and evaluates the morphology of cellular and non-cellular elements in microscopic preparations
	64)	Able to differentiate between clinically significant and insignificant findings to the test done at PHC
	65)	Able to differentiate between clinically significant and insignificant findings to the test done at PHC
	66)	Able to perform point-of-care testing and assess results
	67)	Able to adhere to guidelines for specimen retention, storage, transportation, and disposal
**Recording and Reporting**	68)	Able to report results of all types of tests done in PHCs based on manual reporting like microscopy, color change based, strip tests/ card tests etc. meeting quality control criteria
	69)	Able to understand the standard units used in test reports as agreed between laboratory professionals and clinicians
	70)	Should know the importance of referring to any preliminary report of the same test on the same patient
	71)	Able to compare the results with positive and negative controls
	72)	Able to record the test results to Laboratory Information System or on registers as appliable
	73)	Able to keep records/reporting data in safe custody
	74)	Able to recognize and acts on critical values by timely communicating the critical reports to the concerned physician

Competencies for Medical Laboratory Technicians in Primary Health Care Setting

	75)	Able to release a report within TAT (Turn Around Time)
	76)	Able to confirm that the Laboratory's report distribution/delivery system is efficient, and the report reaches the clinician or patient confidentially and in time
**Laboratory Safety and Laboratory Acquired Infection control**	77)	Able to demonstrate general and specific Safety Precautions of Clinical Laboratory
	78)	Able to use personal protective equipment appropriately, e.g., gloves, gowns, masks, face shields, aprons
	79)	Able to know about laboratory hygiene and infection control practices / Policy
	80)	Able to minimize possible dangers from biological specimens, and the use of biosafety equipment
	81)	Able to use laboratory safety devices, e.g., safety pipetting devices, safety containers, and carriers.
	82)	Able to label, date, handle, store, and dispose of chemicals, dyes, reagents, and solutions according to legislation, e.g., Master Safety Data Sheet (MSDS)
	83)	Able to handle and disposes of sharps (Bio-Medical Waste Policy) with special references to syringes and their needles.
	84)	Able to store, handle, transport, and disposes of biological and other hazardous materials according to Bio-Medical Waste Policy
	85)	Able to use disinfection and sterilization methods to disinfect materials to be used or disposal
	86)	Able to select disinfectant as required
	87)	Able to test the efficacy of Sterilizers
	88)	Able to document all incidents related to safety and personal injury like Needle Stick Injury
	89)	Able to apply the standard precautions to prevent the spread of infection as per organization requirements / local rules and other rules as appliable
	90)	Able to minimize contamination of materials, equipment, instruments, and environment by aerosol and splatter

	91)	Able to follow protocols for care following exposure to blood or other body fluids as required
	92)	Able to place appropriate signs wherever and whenever required, e.g., hazardous, flammable, restricted entry, containment zone etc.
	93)	Able to maintain hand hygiene by washing hands before and after patient contact and/or after any activity likely to cause contamination.
	94)	Able to use alcohol-based hand sanitizers, if justified, e.g., soap and water are not available or any other such barrier.
	95)	Able to deal with sharp cuts and abrasions
