# Supplementary material for: Developing a competency assessment framework for medical laboratory technologists in primary healthcare settings in India
Source: PLoS One. 2024 Apr 1;19(4):e0294939. doi: 10.1371/journal.pone.0294939 (PMC10984544; doi:10.1371/journal.pone.0294939)
Supplement: S3 File — (PDF) [file pone.0294939.s003.Pdf]

Competency Assessment Tool- Medical Laboratory Technologist

A. General Information:

A1. Date & Time:	A4. Designation:
A2. Age:	A5. Health Facility:
A3. Gender:	A6. District:

		High School	Intermediate	Technical Education
A7. Education & In Service Training:	Place			
	Year of Completion			

A8. Postings details	Place of Posting	District	Period (From-to)	Regular/ Contractual	Reason(s) for transfer
1					
2					
3					
4					
5					

B. General Questions (Self Appraisal):

B1. Do you know, how many tests are provisioned for your facility?
B2. How many laboratory tests are being done in this facility? Can you name those tests?
B3. In your opinion, how competent you are to perform your day-to-day activities?
B4. In a scale of 1-5, how would you rate your overall clinical / technical knowledge & skills to perform your duty?<br><br>(1-very little, 2- less, 3- adequate, 4- more than needed, 5-exceptional)
B5. What competencies you have, those are of use the most in carrying out the assigned duties?
B6. Do you feel that you have additional skill sets to perform tasks, other than the assigned duties to you, competently?

B7. In your opinion, what other competencies do you need to perform better in your job?
B8. What all trainings have been given to you to make yourself competent to carry out the assigned duties?
B9. Is there any mechanism to provide training/ orientation before introduction of any new sample collection tool?
B10. What additional trainings are required for you to carry out your duties, efficiently?
B11. Do you feel that you are performing well or at least, at par with your expectations in this job?<br><br>If yes/no, then why do you feel so?
B12. How would you describe your working conditions, here at this facility?
B13. What are the working conditions, which enables / disables you to carry out your duties?
B 14. What is the supply chain mechanism to ensure the availability of adequate diagnostic supplies and consumables, equipment in your facility/ laboratory?

C. Competencies Domains[1]

Competencies Domains	Codes
**Human Values and Professional Ethics**	1
**Quality Management**	2
**Communication and Interaction**	3
**Critical Thinking**	4
**Equipment Instruments and Consumables**	5
**Test Requisition Data, Sample collection and transportation**	6
**Specimen Preparation**	7
**Assessment & Analysis**	8
**Recording and Reporting**	9
**Laboratory Safety and Infection Control**	10

This tool is meant to assess the competencies of Medical Laboratory Technologist in terms of **Knowledge[2]**, **Skills[3]** and **Attitudes[4]** through **Checklist**, **Direct Observation of Procedural skills**, **Mini Clinical Laboratory Evaluation**.

C.1. Questionnaire (Knowledge and Attitude)

Domain Code	S. N.	Points	Knowledge/ Attitude/ Skill	Response (Yes/No)	Remarks
1	1.	Do you understand the terms "human values and ethics"?	Knowledge		
	2.	Do you feel that human values and ethics are important in delivering Laboratory Services at your facility?	Attitude		
	3.	Do you understand the purpose of maintaining confidentiality for health care Information?	Knowledge		
	4.	Do you think, maintaining confidentiality for health care information should be must in Primary Health Centres?	Attitude		
	5.	Do you think, it is important for Medical Laboratory Technologist to respect the diversity, dignity, values	Attitude		

[1] Broadest Category of Competencies
[2] Concepts and Theories
[3] Use of techniques to integrate knowledge into practice
[4] A person's feelings, values, and beliefs, which influence their behaviour and the performance of tasks

		and beliefs of patients /clients and colleagues, for delivering Laboratory Services?			
	6.	Do you know what the critical components for continual improvement for laboratory Services are?	Knowledge		
	7.	Do you feel the need of improvement in your work?	Attitude		
	8.	Do you know what are the legislations/ regulatory bodies that governs medical laboratory in your facilities?	Knowledge		
	9.	Do you know what the compliances are required at your institutions regarding legislation that governs medical laboratory Services?	Knowledge		
	10.	Do you think, legislation compliances that governs medical laboratory services for facility are useful?	Attitude		
	11.	Did you ever find difficulty in executing Laboratory Processes in relation to your own knowledge or skills?	Attitude		
	12.	Do you have any idea, that how could you acquire those knowledge and skills?	Knowledge		
	13.	Do you know about Informed Consent and its use including patients right to refuse?	Knowledge		
	14.	Do you feel, Informed consenting including patient's right to refuse is important for delivering laboratory Services?	Attitude		
	15.	Do you feel that the continuing education and training programs could be helpful, in improving your performance, work environment and career progression?	Attitude		
2	16.	Do you understand the term "Quality Management system" in medical laboratory Services?	Knowledge		

	17.	Do you understand the term "Quality Control including internal and external QC"?	Knowledge		
	18.	Do you understand the term "Quality Assurance"?	Knowledge		
	19.	Do you understand the term "Quality Improvement"?	Knowledge		
	20.	In your sense, do you think Quality assurance is important for improving laboratory services?	Attitude		
	21.	If no to above 5 questions, then, Would you like if Quality Management System implemented in your laboratory?	Knowledge		
	22.	Do you maintain and display turnaround time (TAT) for all the tests done in your laboratory specific to each test?	Knowledge		
	23.	Do you feel that the concept of consistency, reproducibility and Turnaround time is important for delivering laboratory services?	Attitude		
	24.	Do you think, the application of quality control concept can improve the quality of laboratory test reports?	Attitude		
	25.	Do you Know the concept of Corrective Action and Preventive Action (CA-PA)?	Knowledge		
	26.	Do you know the concept of "Root cause analysis" ?	Knowledge		
	27.	Do you think, the concept of root analysis is useful in taking preventive actions in case of any incidents/accidents and observable quality degradation?	Attitude		
	28.	Do you know about the protocols defined in the quality policies, process, and procedure manuals, if any ?	Knowledge		
	29.	Do you feel the protocols as defined in the quality policies, processes and procedure manuals are useful	Attitude		

		in ensuring quality laboratory Services?			
	30.	Do you know how to make the arithmetic calculations like Average, simple multiplications, divisions etc?	Knowledge		
	31.	Do you think, simple statistics like, mean, average calculations knowledge helps you in conducting and generating lab test report?	Attitude		
	32.	Do you know about Calibration and preventive maintenance of equipment?	Knowledge		
	33.	Do you think calibration and validation of equipment and the test procedures are important for proper test results?	Attitude		
	34.	Do you feel that proper maintenance and preventive measures in relation to equipment are important for their proper functioning?	Attitude		
	35.	Do you know that the equipment should be tested in routine for their efficacy?	Knowledge		
	36.	Do you know about First in and First Out (FIFO)?	Knowledge		
	37.	Do you feel that FIFO is useful in maintaining inventory for reagents?	Attitude		
4	38.	Do you know about valid priorities (Coagulation Studies prior to emergency Surgery, Blood grouping in accidental and other medical emergencies) during clinical testing?	Knowledge		
	39.	Do you feel that importance should be given to valid priorities	Attitude		
	40.	Do you know the concept of Critical alerts?	Knowledge		
	41.	Suppose you have come to know that immediate communication of test results can save the patient's life, will you send the critical alerts to the concerned?	Attitude		

	42.	Do you know the importance of 4 M (Money, Man, Material, and Minutes)?	Knowledge		
	43.	Do you feel that the right use of 4M would help in efficient functioning of your laboratory?	Attitude		
	44.	Do you know about higher education in your profession?	Knowledge		
	45.	Do you think that higher education will improve your professional work quality?	Attitude		
5	46.	Do you know about calibrators, standards and other quality control materials?	Knowledge		
	47.	Do you prefer to use calibrators, standards, and other quality control materials? (pH Meter- Acidic, alkaline and neutral calibrator/ Standards, Weighing Balance- Weights)	Attitude		
	48.	Do you know the importance of Shelf life of reagents, chemicals and diagnostic kits etc.?	Knowledge		
	49.	Do you feel the importance of displaying shelf life of inhouse prepared reagents, like Giemsa Stain for blood cell counting and morphology ; Zeil-Neelson stain for Acid fast Bacilli; Buffers for many other reagents, e.g. for biochemistry test?	Attitude		
	50.	Do you know about the use of positive and negative controls while processing the test samples?	Knowledge		
	51.	In your sense, do you feel that use of positive and negative controls for tests is important?	Attitude		
	52.	Do you know about the work principles of equipment/instruments used in your laboratory, e.g. Autoclave; Hot Air oven, Sahli's apparatus, Neubauer counting chamber?	Knowledge		

	53.	Do you know about Neubauer counting chamber?	Knowledge		
	54.	Do you know about the concept of risk assessment for any processes or using instruments/ equipment?	Knowledge		
	55.	Would you prefer to conduct risk assessment for processes or using instruments/ equipment?	Attitude		
	56.	Do you feel the need of regular maintenance for maintaining equipment/ instrument ?	Attitude		
	57.	Do you feel the importance of informing competent authorities in case any equipment goes out of order?	Attitude		
	58.	Do you know all the equipment/instrument should be labelled with their respective unique I.D.s, date of purchase, date of installation, date of putting into service, date of the last calibration, and name and contact of address mechanic whom to inform in case of emergency?	Knowledge		
	59.	Do you feel that it is important to label all the equipment/instrument with their respective unique I.D.s, date of purchase, date of installation, date of putting into service, date of the last calibration, and name and contact of address mechanic whom to inform in case of emergency?	Attitude		
	60.	Do you know what SOPs are?	Knowledge		
	61.	Do you feel preparation and display of SOPs for all the processes and equipment are useful?	Attitude		
6	62.	Do you know what the components of test requisition forms are?	Knowledge		
	63.	Do you consider that the following components are important to be included in the test requisition form ?	Attitude		

		Test requisition form: Name, age, sex, Patient ID, Sample and Test required, Clinical diagnosis, Previous investigation result if any, Clinician Identity, Date, Box for Sampling date and time. Signature of recommending doctor, name of Ward/OPD/Clinic			
	64.	Do you consider that the conformity of patient identification before collection of any sample is very much important?	Attitude		
	65.	Do you know that the capillary blood sample should be taken by using a Lancet and not by a needle?	Knowledge		
	66.	Do you know that the instruction to be given to the patient in relation to the sample collection, for example<br><br>1. "How to collect a urine sample for microbial culture from a male or a female patient?"<br>2. How to collect urine sample for routine examination and for 24 hour urine protein analysis?<br>3. How to collect throat sample from suspected cases of diphtheria?<br>4. Instructions to patients to collect sputum sample from suspected pneumonia or Tuberculosis cases?	Knowledge		
	67.	Do you think, is it important to give instruction to be given to the patient in relation to the sample collection? for example<br><br>1. how to collect a urine sample for microbial culture from a male or a female patient?<br>2. How to collect urine sample for routine examination and	Attitude		

		for 24-hour urine protein analysis<br>3. How to collect throat sample from suspected cases of diphtheria.<br>4. Instructions to patients to collect sputum sample from suspected pneumonia or Tuberculosis case			
	68.	Do you consider that the patient counselling/ preparation before clinical sample collection?	Attitude		
	69.	Do you know about the sample collection tools, provisioned at your hospital?	Knowledge		
	70.	Do you know that 70% alcohol works better than 95% alcohol for skin disinfection at the time of blood sample collection?	Knowledge		
	71.	Do you know that after collecting the sample, if transportation is required, the sample is carried in closed sample carrier, with the conditions as per test required?	Knowledge		
7	72.	Do you know about the suitability properties of samples for its intended sample test done in his laboratory? (Should explain at least one example like; Renal Function Test and Liver Function Tests require clear serum of the patient blood but if the blood sample is haemolysed it is not suitable for these tests to be performed)	Knowledge		
	73.	Do you know how to prepare the specimen for analysis?	Knowledge		
8	74.	Do you know all the procedures for processing the samples for various tests available in your laboratory?	Knowledge		
	75.	In spite of your capability to remember all the procedures in your mind, do you feel that preparation and the display of SOPs	Attitude		

		of all the procedures and the use of equipment are important?			
	76.	Do you know about various types of microscopes, and which one is available in your lab?	Knowledge		
	77.	Do you know about the source of interference?	Knowledge		
	78.	Do you prefer to compare your test result with pre-established standard?	Attitude		
	79.	If your test result is not acceptable, would you like to find out any source of interference?	Attitude		
	80.	Do you know how to make test wise corrective actions, root cause analysis and preventive action?	Knowledge		
	81.	Do you prefer to put effort to make corrective and preventive actions?	Attitude		
	82.	Do you know about all the diagnostic kits and Card based Immunoassay tests done in your lab?	Knowledge		
	83.	Do you know about the precautionary steps that affect the test results?	Knowledge		
	84.	Do you know about the working principles of commonly used analysers/semi-auto analysers in your laboratory?	Knowledge		
	85.	Do you know about the acceptable reference ranges of test results, example; Normal Values of various parameters like; Blood Urea, creatinine, glucose levels etc.	Knowledge		
	86.	Do you know about the critical values of test results and alarming critical alerts?	Knowledge		
	87.	In case you observe any test results in critical range (Life threatening), would you like to inform the clinician immediately, by taking extra efforts?	Attitude		

	88.	Do you know about point of care testing?	Knowledge		
	89.	In case of emergency or physical inability to take patient to the laboratory, would you like to go at sight for possible laboratory tests?	Attitude		
	90.	Do you know that the clinical Specimens are required to be retained for some specific period even after successful completion of tests?	Knowledge		
	91.	Do you know how the clinical samples are stored during the retention period?	Knowledge		
	92.	In case, the sample is supposed to be transported to some other laboratory, do you know the procedures for packing and transportation of the sample under suitable condition maintaining the sample integrity intact?	Knowledge		
	93.	Do you know how to dispose of various clinical samples after use /after completion of retention period as per prevailing/applicable biomedical waste management rule?	Knowledge		
	94.	Do you understand the concept of “segregation at source” in relation to the Bio Medical Waste Management?	Knowledge		
9	95.	Do you know about the concept of transcript check?	Knowledge		
	96.	Do you feel that the third-party transcript check is beneficial to medical laboratory services?	Attitude		
	97.	Do you know the standard units used in test reports of various parameters, like the units used in blood sugar, Hb, TLC, DLC, and platelets count etc.	Knowledge		
	98.	Do you feel that the physician should mention on the test	Attitude		

		requisition form if the same test has been done on the same patient recently?			
	99.	Do you know the importance of knowing the previous test report, if the same has been done on the same patient recently?	Knowledge		
10	100.	Do you know about general (Chemical, Biological and non-biological like electrical safety, fire safety) and specific safety precautions in clinical laboratory like, how to deal with blood spill, exposure of your skin to acid or alkalis etc?	Knowledge		
	101.	Do you know about the major and minor blood spills?	Knowledge		
	102.	Do you know that blood spills, should be dealt with pre-set standard procedures ?	Knowledge		
	103.	Do you know what are the precautions required to deal with fire safety?	Knowledge		
	104.	Have you ever been trained/ participated in any fire safety training/ mock drill?	Knowledge		
	105.	Do you know which precautions are required to deal with electrical safety?	Knowledge		
	106.	Do you know that in the electrical fittings, proper earthing is important to avoid electric shocks from any metallic equipment and its damage?	Knowledge		
	107.	Have you ever considered, proper earthing in electrical circuit is important to avoid electric shocks from any metallic equipment and its damage?	Attitude		
	108.	Do you know about the laboratory hygiene and infection control practices / Policy in the laboratory?	Knowledge		

	109.	If there is an infection control policy, do you feel it is worthful to follow?	Attitude		
	110.	Do you know what are the biosafety equipment?	Knowledge		
	111.	Do you know the difference between laminar airflow and a biosafety cabinet?	Knowledge		
	112.	Do you know that the micropipettes or auto pipettes should be used in place of mouth pipetting?	Knowledge		
	113.	Do you know that the labelling dating, handle, store and dispose of chemicals, dyes, reagents, and solutions should be as per applicable legislations is mandatory?	Knowledge		
	114.	Do you know that every chemical accompanied by a document called as Master safety data sheet (MSDS), which explains "how that chemical can be used safely" explaining specifically the safety precautions specifically to all the requirements?	Knowledge		
	115.	Are you aware of that the burning type of syringe needle destroyers are not recommended these days? if not then why?	Knowledge		
	116.	Do you know that how does the mechanical syringe needle destroyers are safe to be used?	Knowledge		
	117.	Do you know that the disinfection and sterilization are different processes?	Knowledge		
	118.	Do you know the different type of disinfectants used in the laboratory for disinfection of floors, work benches, environmental disinfections and discarding jars?	Knowledge		
	119.	Do you know different group of disinfectants and their application?	Knowledge		
	120.	Do you use autoclave or hot air oven?	Knowledge		

	121.	Do you know the concept of efficacy testing of sterilizers?	Knowledge		
	122.	Do you check the efficacy of sterilizer used in your laboratory?	Attitude		
	123.	Do you know about Needle Stick Injuries?	Knowledge		
	124.	In case of needle stick injury, do you contact the physician for further action?	Attitude		
	125.	Do you know about the needle stick injury recording process?	Knowledge		
	126.	In the case of breakage of glass tubes containing clinical samples or some microbial suspension, inside the centrifuge machine, do you know how to handle this situation?	Knowledge		
	127.	Do you wear masks and gloves while working in the laboratory?	Attitude		
	128.	Do you know about the type of infection, that could be acquired during sample collection?	Knowledge		
	129.	Do you know about the precautions measure while working in the laboratory?	Knowledge		
	130.	Do you know that laboratory environment can be contaminated while working with clinical samples/ live organisms?	Knowledge		
	131.	Do you know the protocols could be followed in case of exposure to body fluids (as applicable)	Knowledge		
	132.	In case, there are no established protocol, do you think, these are required to be established and followed?	Attitude		
	133.	Do you know that hands are the most important vehicle for microbial transmission?	Knowledge		
	134.	Do you feel that handwashing is important particularly before and after contact with the patient or patient sample or any other	Attitude		

		activities likely to cause contamination?			
	135.	Do you know that the proper handwashing with soap and water reduces hospital acquired infections by more than 80 %?	Knowledge		
	136.	Do you know under what circumstances alcohol-based hand sanitizer are recommended for use?	Knowledge		
	137.	Do you know that if you encounter any sharp cuts, then how will you respond?	Knowledge		
	138.	In case of sharp cuts, do you feel the need to report to medical officer/ physician nearby?	Attitude		

C.2. Observational Tools (Skills)

Score Definition

A. None—No demonstrated skills at all/does not perform the task(s) completely
B. Limited Demonstrated very limited strengths/skills in this area
C. Some—Demonstrated some ability/skills in this area.
D. Strong—Demonstrated strong skills/strength in this area.
E. Excellent—Demonstrated excellent skills/strength in this area.
F. Not applicable
G. Don't know- Not even heard about that skill
H. Skill limitation is clearly related to resource limitations

Domain Code	S. N.	Observation Points	Means of Verification	Response (Score)
1	1.	Responsibilities towards Patients and their attendants	Listens to patient's ideas and concerns.<br>Doesn't rush patient and doesn't take too much time; Maintains speaking in low pitch voice; Provides documentation that is accurate and complete	
	2.	Respects diversity, dignity, values, and beliefs	Verbal and nonverbal communication with the Patients/Attendants having Physical differences (Racial Difference, Skin colour, texture, facial features); Ethnicity; Gender; Age; Social Class; Sexual Orientation; Differently abled;	
	3.	Compliances towards legislation that applies for Medical Laboratory Services in that facility	Documentation in relation to ; Notifiable Diseases, Biomedical Waste Management Rules 2016 , Minimum Standards of Medical Diagnostic Laboratories as per notifications from Government bodies from time to time like; Clinical Establishment Act	
	4.	Prior Informed Consent from the patient/ Patient Representative	Procedure explanation, Potential harm and benefits, Respects Patients' right to refuse	
2	5.	Quality Control	Check internal Reference Samples and External Reference documentation related to affiliation with some EQAS Agencies for example- Indian Association of Medical Microbiologist (IAMM) runs Medical Microbiology EQAS	

	6.	Reproducibility	Documentation to be checked in relation to the reproducibility assessment-for example-One sample is tested for one parameter under the same conditions using same facility, same reagents by the same person, for two or more times should give the same result.	
	7.	Corrective Action and Preventive Action (CA-PA)?	Documentation in terms of<br>-Incident/Accident Log/Record<br>-CA-PA Log/ Record	
	8.	Root Cause Analysis and preventive actions in case of any incidents/accidents and observable quality degradation	Root cause Steps-<br>• Define the Problem<br>• Find Causes<br>• Find root Cause<br>• Find Solutions<br>• Take Actions	
	9.	Follow Protocols as defined in the quality Process and procedure manuals	Verification of patient /specimen identification<br>Specimen collection and transport<br>Specimen receipt<br>Specimen rejection criteria<br>Specimen handling,<br>Preparation, and storage<br>Specimen acceptance and rejection<br>Sample custody (Chain of custody forms, etc.)<br>Data entry and review	
	10.	Simple calculations	Final Result of Test like ;<br>A. Conjugated, Non conjugated and Total Bilirubin<br>B. Calculation of Prothrombin Index (PTI)	
	11.	Calibration	Internal Calibration of Commonly used equipment e.g., Weighing Balance; and Micro Pipettes etc and **check their calibration records**	
	12.	Preventive Maintenance	Hot Air Oven; Autoclave, Weighing Balance, Haemoglobin Testing Equipment (Sahli's apparatus or colourimeter) any type of Analyzer etc.<br>If done, **check records.**	
	13.	Efficacy Testing	Hot Air Oven; Autoclave or any other sterilizer; Incubators; pH meters etc<br>If done, **check the records.**	

	14.	First in First Out (FIFO)	Whether First in First out concept is followed?<br><br>Verify the following?<br><br>1. Check the Inventory for any expired materials kept in the stock including perishable items in refrigerator<br>2. Match the stock the with the stock register or digital data., if any , as applied therein<br>3. Check that the recently procured items are kept serially behind the previously procured items<br>4. Whether the stocks are placed as per their hazardous category requirements for example A. Alcohols are stored separately under lock B. Acids are always kept separately on floor only.	
3	15.	Communication	A. Active listening<br>B. Verbal communication<br>C. Non-verbal communication<br>D. Written Communication-Check documents like – Leave application; Indenting to Procure required Items etc.<br>E. Using technology appropriately to facilitate communication, like emails; WhatsApp messaging etc.	
	16.	Terminology/ Abbreviations related to Medical Laboratory Testing	Examples Hb, TLC, DLC, RA Factor, Rh Factor, CRP, HIV, RTPCR, Urine R/E etc.	
	17.	Effective interdisciplinary/intra professional Communication & his/her role clarification in the team	To observe his/her interdisciplinary/intra professional Communication.<br><br>Whether he fulfils his role in the medical team	
5	18.	Use of Calibrators/ Standards	Check the documentation<br><br>Check the standards- weights for routine calibration of weighing balance and pH Meter- Standards	

	19.	Shelf life/ Expiry Date	Whether you observe -Chemicals, Reagents and commercial kits etc. used in your laboratory before proceeding for test.	
	20.	Use of positive and negative control for tests	Availability of Positive and negative control reagents<br><br>Check the use log of controls samples or reagents	
	21.	Regular maintenance	Check the regular maintenance Records	
	22.	Labelling of equipment/instrument	Respective unique I.D.s,<br>date of purchase,<br>date of installation,<br>date of putting into service,<br>date of the last calibration, and<br>Name and contact of address mechanic whom to inform in case of emergency	
	23.	SOPs for equipment/instrument use, and the test procedures	Check the various SOPs maintained and displayed by the concerned laboratory professional	
6	24.	Test requisition form	Test requisition form: Name, age, sex, Patient ID, Sample and Test required, Clinical diagnosis, Previous investigation result if any, Clinician Identity, Date, Box for Sampling date and time. Signature of recommending doctor, name of Ward/OPD/Clinic	
	25.	Sample Collection Instructions	To observe whether he/she gives the instructions to the patient (how to collect the sample) for example;<br>1. how to collect a urine sample for microbial culture from a male or a female patient?<br>2. How to collect urine sample for routine examination and for 24-hour urine protein analysis<br>3. How to collect throat sample from suspected cases of diphtheria.	

			Instructions to patients to collect sputum sample from suspected pneumonia or Tuberculosis case	
	26.	Counselling/ Preparation of patient before clinical sample collection	To observe whether before taking any kind of sample like blood sample , throat swab, he/she explains to the patient about the procedure and he/she is going to prepare the patient psychologically.	
	27.	Use of sample collection tool	1. Test Specific Sample Collection vials (Reusable/Disposable)-<br>A. if recirculated (Glass) vials are being used then whether these are properly prepared Cleaned, sterilized and addition of suitable coagulants as per test requirement<br>2. Syringes and needles swabs as per the test requirement<br>3. For capillary blood, whether lancet is being used, skin disinfectants, absorbent cottons<br>4. Tourniquet Available<br>5. Equipment required to preserve sample till the processing	
	28.	Percentage of alcohol in skin disinfectant used for skin at the time of blood sample collection	Whether he is using alcohol/spirit at the concentration between 70 to 90% ?	
	29.	Use of sample transportation Carrier (Insulated Box)	- Available<br>- Use	
7	30.	Identification and to prepare the samples for processing	Detailed matching of the samples and patient details given on requisition form.	
	31.	Specimen suitability	Should explain at least one example like; Renal Function Test and Liver Function Tests require clear serum of the patient blood but if the blood sample is haemolysed it is not suitable for these tests to be performed.	
8	32.	Adhering SOPs	To observe if any SOPs are displayed and followed	
	33.	Manual Counting of Blood cells	To observe whether the professional is able to perform the manual blood cell counting like, TLC, DLC, Platelets count etc.	

	34.	Manual Biochemistry Tests	To observe whether the professional is able to perform manually the common biochemistry tests example-Blood Sugar, Urea, Creatinine, electrolytes, Urine sugar, urine proteins, pregnancy tests etc.	
	35.	Microscopic Analysis of Clinical samples for cell morphology and counting	To observe whether the professional is able to perform for various clinical samples for cell morphology and counting. Example- Urine and other body fluid microscopy	
	36.	Reference Values	To observe whether reference values of each test results have been displayed in the laboratory or on the test requisition form	
	37.	Point of Care Testing	Observe any list of tests for Point of Care Testing, If yes, please observe the availability of consumables and portable equipment, required for the tests	
	38.	Specimen retention, storage, transportation, and disposal	Whether the samples are being retained? if yes, then whether stored at suitable conditions, maintaining the sample integrity<br><br>To observe whether the professional is skilled for packing of clinical sample to transport safely.<br><br>To observe whether the laboratory is equipped with the colour coded BMW Baskets.<br><br>Whether “segregation at source” is being followed or not	
9	39.	Recording and Reporting	Observe the recording of results (Rough and final) - Sample Identity (Transcript Check)	
	40.	Standard Units used in Recording and Reporting	Observe for tests like the units used in blood sugar, Hb, TLC, DLC, and platelets count etc.	
	41.	Positive and negative control Results to be matched with their known values	Observation – Before final report recording, does professional match the positive and negative control values with their actual and known values respectively.	
	42.	Test Result Reporting	Observe the record- either Laboratory Information System or registers.	

	43.	Laboratory data Safety	Observe the accessibility to the laboratory records by any un-authorized persons.<br><br>Records kept under lock.<br><br>Maintains the charge handover takeover records, while during shift change or while leaving for or joining back from leave or holidays.	
10	44.	General Safety precautions in medical laboratory	To demonstrate dealing with major and minor blood spills in and outside the equipment.<br><br>Observe his/ her laboratory for fire extinguisher which has been validated within year.<br><br>Observe the documentation related to fire safety training/ mock drill.<br><br>Observe for any the naked electrical connection /wire	
	45.	Laboratory Safety	To observe what type of pipetting devices are being used in the laboratory.<br><br>Use of leak proof sample containers and their carriers (For sample collection and/ or for transportation of samples)	
	46.	Chemical Safety	To observe if any MSDS has been recorded in the laboratory documentation.<br><br>Observe that the chemicals have been kept following their hazardous category.	
	47.	PPE Use	Observe how the professionals equip themselves with the PPE.	
	48.	laboratory hygiene and infection control practices / Policy	Whether the laboratory has any written / documented and displayed infection control policy.<br><br>If yes, does the professional follow that policy?	
	49.	Inventory Management	Observe that the labelling dating, handling, storing, and disposing of chemicals, dyes, reagents, and	

			solutions are as per applicable rules and regulations/MSDS.	
	50.	Disposal of used Syringes and needles	To observe how does the needles and syringes are being disposed of?<br><br>Whether the laboratory has dedicated functional syringe needle destroyer<br><br>What type of syringe needle destroyer you have: burning type and mechanical cutting based?	
	51.	Selection of disinfectants to be used in discard Jars	To observe "what type of disinfectants are being used in the laboratory where the professional is working?	
	52.	Sterilizer Efficacy	To observe the process testing of sterilizer	
	53.	Dealing with spill inside centrifuge machine	To observe "how the professional deals with a broken contaminated tube within the centrifuge machine"	
	54.	Appropriate signage	To observe for appropriate signage for hazardous, flammable, restricted entry, containment zone etc.	
	55.	Hand hygiene	To observe whether the professional follows the proper hand hygiene protocol as per WHO Protocol.	
	56.	Sharp Cuts	To observe the register/ documented record in which sharp cuts are being recorded.	

C.3. Mini Clinical Laboratory Skills

Score Definition

A. None—No demonstrated skills at all/does not perform the task(s) completely
B. Limited Demonstrated very limited strengths/skills in this area
C. Some—Demonstrated some ability/skills in this area.
D. Strong—Demonstrated strong skills/strength in this area.
E. Excellent—Demonstrated excellent skills/strength in this area.
F. Not applicable
G. Don’t know- Not even heard about that skill
H. Skill limitation is clearly related to resource limitations

Dom.	S.N.	Clinical Evaluation Points	Means of Verification	Response (Score)	Remarks
5	1	Functional Quality check for Reagents /diagnostic Kits	Normal blood smear to be stained with the existing Giemsa staining solution and to demonstrate all the blood cells with accurate & expected color and morphology<br><br>Mixture of Staphylococcus aureus and E. coli shall be smeared on a glass slide and stained with gram staining procedure. After staining if the gram positive or gram-negative organisms are clearly differentiated then staining solution is okay.		
	2	Use of Neubauer counting chamber for blood cell counting on a sample of know counts	Preparation of diluents<br><br>Making dilution of the blood samples<br><br>Filling Neubauer counting chamber<br><br>Focusing under Microscope (40X)<br><br>Cell Counting and calculations		
6	3	Demonstration of Sample collection tools	Whether he/she is able to demonstrate sample collection tool like; sterile syringe and needle, lancet, cotton /rayon swab sticks, suitable anticoagulants, and sample		

			collection containers for the samples, which are routinely processed.		
	4	Venipuncture	16 Steps (From labeling the tube with the patient's particulars to place request form and specimen in biohazard bag)		
	5	Use of anticoagulants for specific tests	Proper and suitable coagulants in sample collection vials as per the test requirement like; requirement of serum, plasma and whole blood- for hemogram is done in EDTA (Ethylenediaminetetraacetic acid) Containing sample vials; and for blood sugar estimation, sample should be collected in sodium floride containing vial, and the general biochemistry test, no anticoagulants should be in the vial.		
	6	Chain of Custody procedures	A. Labelling the sample with patient ID and tests required as per traceability protocol; uploading the sample related data on Information systems or on hard copies like registers, files.<br>B. Preservation of samples till it processed- keeping sample integrity intact (Conditions- temperature etc).		
7	7	Specimen Preparation for analysis	Example 1:<br>Should be able to process blood sample for separation of serum/ plasma to be subjected to analysis.<br>Example 2:<br>To dilute the blood sample in the suitable diluent for different blood cell counts.<br>Example 3<br>To prepare body fluids or other clinical samples for microscopic examination.		

	8	Staining Procedures	Able to demonstrate the principles, reagents used and procedures of various staining methods used in his/her laboratory.<br>Example 1. Preparation of thin and thick smear of blood for detection of malarial parasite.<br><br>Example 2. Various staining procedures available in the lab for detection of malarial parasite.		
	9	Microscopy	Should be able to focus the smear efficiently under the microscope and observe for abnormalities therein in relation to the expectation of the test. For example- can he find malarial parasite present in the blood film under the microscope.		
	10	Light measuring Instruments	He/she should be able to use light measuring instruments like Colorimeter, spectrophotometer. Flame photometer, reflectometry; turbidimetry, whatever is available in the lab.		
	11	Use of commercially procured diagnostic kits including Card based Immunoassays	To observe the professional doing anyone of the commercially available kit test.		
	12	Manual Testing	Any		
10	13	Spill Management	To observe the professional dealing with the blood spill into the laboratory.<br><br>Specific Observation:<br>1. Whether he wears PPE before dealing with spill.<br>2. Which disinfectant and at what concentration, he uses to deal with spill.<br>3. Whether follows the right procedures.		

C.4. Case Study

Domain	Case	Response (Satisfactory/ Unsatisfactory)	Remarks
4	**Able to organize work to accommodate valid priorities- for example-**<br><br>Last month, one patient Rima landed your hospital with gestational complications. Medical Officer of your facility identified this as critical case that needs immediate lower abdomen Caesarean section. The doctor on duty sent her blood sample to your laboratory for D-dimer. At the same time, a patient comes to doctor saying that he is feeling weak and feeling pain in his legs. Symptomatically, doctor wishes to rule out the diabetes and sends the sample to laboratory for blood sugar testing. Under the circumstances, to which sample you will prioritize to process.		
	Able to demonstrate effective problem solving/trouble-shooting strategies and initiates the appropriate follow-up **for example-**<br><br>Suppose you have received one sample from the physician who wishes to know whether or not the sample contains any gram positive or gram-negative microbe in it. Per chance you found that your gram stain kit is out of stock. On enquiring from the store, the storekeeper informed that except gram staining kit everything is available in the store. Under the circumstances, how would you be able to perform gram staining and help the clinician in making diagnosis of concerned patient?		As the store-keeper clarified that he has everything except gram staining kit, the professional should get the chemicals (Crystal Violet, Safranin, Acetone, and Alcohol) , issued from the store, which are required to prepare the gram staining reagents manually and perform the procedure.
5	Risk assessment for any processes or in using instruments/ equipment **for example-**		He will assess that -1. the machine is properly working

	Suppose you wish to centrifuge the blood samples to separate serum. What type of risks, may be there, which need to be assessed so that centrifugation is expected to be done as risk free?		2. There is nothing unrequired inside the chamber like; broken glass, any type of spilled fluid etc.<br>3. The shock absorbing cushions are present in all the test tube cups.<br>4. The test tubes and cups are properly balanced.<br>5. Ensure that the lid is properly locked, before starting the machine.<br>6. Increases the speed slowly not with the sudden jerk.<br>7. After completion of centrifugation time and switching it off he waits till the rotor stops automatically.<br>8. Whether he waits minimum for 5-10 Minutes allowing the aerosols to settle if any before opening the lid.
8	Critical Alerts:<br>**for example-**<br>A sixty-year-old patient approaches the emergency with some problems and symptomatically the physician expects it as a hypoglycemic case and sends		Since the blood glucose level of the patient is very low and patient may enter into

	his blood sample for blood sugar testing. You performed the test and found that his blood glucose level is 40 mg/dl. What will your further action?		coma phase, considering it critical the laboratory professional should convey the report immediately to the physician, as a critical alert so that the patient may be saved.
	Inform competent Authorities for Out of order equipment-**for example**<br><br>Suppose you were working with the semi auto analyzer or a centrifuge machine or any mandatory equipment required to perform the test. You have talked to the engineer concerned immediately who informed you that the instrument will take seven days to be functional. What would be your immediate action ?		The first action should be to inform the higher authorities. Secondly an immediate circular/ intimation should be released to all the concerned medical officers that because of an out of order equipment these tests can not be performed till seven days and when the equipment will be ready a subsequent notification will be released, hence the laboratory shall not receive any sample related to concerned tests till the further notice.

Annex-1 List of Diagnostics Tests [5]

Essential

Haemoglobin, Total Leucocyte Count, Differential Leucocyte Count, Platelet count, Complete Blood Count, Erythrocyte Sedimentation Rate, Blood Group And Rh Typing, Blood Cross Matching, Peripheral Blood Film, Reticulocyte Count, Absolute Eosinophil Count, Bleeding Time And Clotting Time, Sickling Test For Screening Of Sickle Cell Anemia*, Sickle Cell Test Rapid For Screening Of Sickle Cell Anemia (Strip Test) *, NESTROFT Test for screening of Thalassemia*, DCIP test for screening HbE hemoglobinopathy*, Screening test for G6PD enzyme deficiency, MP slide method, Malaria rapid test, Human chorionic gonadotropin (HCG) (Urine test for pregnancy), Urine test for ph, specific gravity, leucocyte esterase, glucose, bilirubin, urobilinogen, ketone, protein, nitrite, Urine Microscopy, Urine for microalbumin, Stool for ova and cyst, Stool for Occult Blood, RPR/VDRL test for syphilis, HIV test (Antibodies 1/2 and HIV 1/2), Hepatitis B surface antigen test, HCV Antibody Test (Anti HCV), Sputum, pus etc. for AFB, Typhoid test (IgM), Blood sugar, Glucose Tolerance test (GTT), S. Bilirubin (T), S. Bilirubin direct and indirect, Serum creatinine, Blood Urea, SGPT, SGOT, S. Alkaline Phosphatase, S. Total Protein, S. Albumin & AG ratio, S. Globulin, S. Total Cholesterol, S. Triglycerides, S.VLDL, S.HDL, S. LDL, S. Uric acid, Glycosylated haemoglobin (HbA1C), Serum Calcium, Wet mount and Gram stain for RTI/STD, Gram staining for clinical specimen, Throat swab (Albert stain) for Diphtheria, Stool for hanging drop for Vibrio Cholera, Visual Inspection Acetic Acid (VIA), rK39 for Kala Azar, TB – Mantoux, Troponin – I, Pap smear

Desirable

D- Dimer, S. Sodium, S. Potassium, Magnesium, Test for Filariasis, S.TSH (including for new-born screening), CRP (including new-born) (Quantitative)

Annex-2 List of Equipment[5]

Essential

Hemoglobinometer, 3 Part Hematology analyzer*, Manual with reading using, Blood group kit (manual), Microscopy, Manual, Rapid Card test, Manual with microscopy/Solubility test/Cover slip test, Rapid card tests for combined P. Falciparum and P. vivax, Multiparameter urine strip (dipstick), Turbidometer/Nephelometer, Manual Kit, Glucometer, Semi Automated Biochemistry analyzer*, Wet mounting, gram staining, Microscopy/Filaria Strip test, HemeChip Point of Care device (D)

Desirable

ESR Analyzer, Turbidometer, Indirect ion selective electrode Electrolyte Analyzer*,

** For Hub Lab*

[5] Indian Public Health Standard for Health and Wellness Centre, Primary Health Centre, 2022
