# Supplementary material for: Developing a competency assessment framework for medical laboratory technologists in primary healthcare settings in India
Source: PLoS One. 2024 Apr 1;19(4):e0294939. doi: 10.1371/journal.pone.0294939 (PMC10984544; doi:10.1371/journal.pone.0294939)
Supplement: S4 File — (PDF) [file pone.0294939.s004.pdf]

Competency Assessment Tool- Medical Laboratory Technician

A. General Information:

A1. Date & Time:	A4. Designation:
A2. Age:	A5. Health Facility:
A3. Gender:	A6. District:

A7. Education & In Service Training:		High School	Intermediate	Technical Education
	Place			
	Year of Completion			

A8. Postings details	Place of Posting	District	Period (From-to)	Regular/ Contractual	Reason(s) for transfer
1					
2					
3					
4					
5					

B. General Questions (Self Appraisal):

B1. Do you know, how many tests are provisioned for your facility?
B2. How many laboratory tests are being done in this facility? Can you name those tests?
B3. In your opinion, how competent you are to perform your day-to-day activities?
B4. In a scale of 1-5, how would you rate your overall clinical / technical knowledge & skills to perform your duty?<br><br>(1-very little, 2- less, 3- adequate, 4- more than needed, 5-exceptional)
B5. What competencies you have, those are of use the most in carrying out the assigned duties?
B6. Do you feel that you have additional skill sets to perform tasks, other than the assigned duties to you, competently?
B7. In your opinion, what other competencies do you need to perform better in your job?

B8. What all trainings have been given to you to make yourself competent to carry out the assigned duties?
B9. Is there any mechanism to provide training/ orientation before introduction of any new sample collection tool?
B10. What additional trainings are required for you to carry out your duties, efficiently?
B11. Do you feel that you are performing well or at least, at par with your expectations in this job?<br><br>If yes/no, then why do you feel so?
B12. How would you describe your working conditions, here at this facility?
B13. What are the working conditions, which enables / disables you to carry out your duties?
B 14. What is the supply chain mechanism to ensure the availability of adequate diagnostic supplies and consumables, equipment in your facility/ laboratory?

C. Competencies Domains[1]

Competencies Domains	Codes
**Human Values and Professional Ethics**	1
**Quality Management**	2
**Communication and Interaction**	3
**Critical Thinking**	4
**Equipment Instruments and Consumables**	5
**Test Requisition Data, Sample collection and transportation**	6
**Specimen Preparation**	7
**Assessment & Analysis**	8
**Recording and Reporting**	9
**Laboratory Safety and Infection Control**	10

This tool is meant to assess the competencies of Medical Laboratory Technicians in terms of **Knowledge[2]**, **Skills[3]** and **Attitudes[4]** through **Checklist**, **Direct Observation of Procedural skills**, **Mini Clinical Laboratory Evaluation**.

C.1. Questionnaire (Knowledge and Attitude)

Domain Code	S. N.	Points	Knowledge/ Attitude/ Skill	Response (Yes/No)	Remarks
1	1.	Do you understand the terms "human values and ethics"?	Knowledge		
	2.	In case of sharp cuts, do you feel the need to report to medical officer/ physician nearby?	Attitude		
	3.	Do you feel that handwashing is not much important particularly before and after contact with the patient or patient sample or any other activities likely to cause contamination?	Attitude		
	4.	Do you know what the critical components (Addressing the complains and feedbacks from Patient, Physician, Staff and learnings from accidents/ Incidents), for continual improvement for laboratory Services are?	Knowledge		
	5.	In case of needle stick injury, do you contact the physician for further action?	Attitude		

[1] Broadest Category of Competencies
[2] Concepts and Theories
[3] Use of techniques to integrate knowledge into practice
[4] A person's feelings, values, and beliefs, which influence their behaviour and the performance of tasks

	6.	Do you know what are the legislations/ regulatory bodies that governs medical laboratory in your facilities?	Knowledge		
	7.	Do you have any idea, that how could you acquire those knowledge and skills?	Knowledge		
	8.	Do you know about Informed Consent and its use including patients right to refuse?	Knowledge		
	9.	If there is an infection control policy, do you feel it is not worthful to follow?	Attitude		
2	10.	Do you understand the term “Quality Control including internal and external QC”?	Knowledge		
	11.	Do you maintain and display turnaround time (TAT) for all the tests done in your laboratory specific to each test?	Knowledge		
	12.	Do you wear masks and gloves while working in the laboratory?	Attitude		
	13.	Do you Know the concept of Corrective Action and Preventive Action (CA-PA)?	Knowledge		
	14.	Do you know about the protocols defined in the quality policies, process, and procedure manuals, if any ?	Knowledge		
	15.	Do you feel that the continuing education and training programs could be helpful, in improving your performance, work environment and career progression?	Attitude		
	16.	Do you know about Calibration and preventive maintenance of equipment?	Knowledge		
	17.	Do you feel the protocols as defined in the quality policies, processes and procedure manuals are not useful in ensuring quality laboratory Services?	Attitude		
	18.	Do you know that the equipment should be tested in routine for their efficacy?	Knowledge		
	19.	Do you know about First in and First Out (FIFO)?	Knowledge		
	20.	Have you ever considered, proper earthing in electrical circuit is important to avoid electric shocks from any metallic equipment and its damage?	Attitude		
4	21.	Do you know about higher education in your profession?	Knowledge		
	22.	In case of emergency or physical inability to take patient to the laboratory, would you like to go at sight for possible laboratory tests?	Attitude		
5	23.	Do you know the importance of Shelf life of reagents, chemicals and diagnostic kits etc.?	Knowledge		

	24.	In case you observe any test results in critical range (Life threatening), would you like to inform the clinician immediately, by taking extra efforts?	Attitude		
	25.	Do you know about the work principles of equipment/instruments used in your laboratory, e.g. Autoclave; Hot Air oven, Sahli's apparatus, Neubauer counting chamber?	Knowledge		
	26.	Do you consider that the patient counselling/ preparation before clinical sample collection?	Attitude		
	27.	Do you know all the equipment/instrument should be labelled with their respective unique I.D.s, date of purchase, date of installation, date of putting into service, date of the last calibration, and name and contact of address mechanic whom to inform in case of emergency?	Knowledge		
	28.	Do you feel that human values and ethics are not much important in delivering Laboratory Services at your facility?	Attitude		
	29.	Do you know what SOPs are?	Knowledge		
	30.	Do you feel that it is wastage of time in labelling all the equipment/instrument with their respective unique I.D.s, date of purchase, date of installation, date of putting into service, date of the last calibration, and name and contact of address mechanic whom to inform in case of emergency?	Attitude		
6	31.	Do you consider that the conformity of patient identification before collection of any sample is very much important?	Attitude		
	32.	Do you feel the importance of informing competent authorities in case any equipment goes out of order?	Attitude		
	33.	Do you know that after collecting the sample, if transportation is required, the sample is carried in closed sample carrier, with the conditions as per test required?	Knowledge		
7	34.	Do you know about the suitability properties of samples for its intended sample test done in his laboratory? (Should explain at least one example like; Renal Function Test and Liver Function Tests require clear serum of the patient blood but if the blood sample is haemolysed it is not suitable for these tests to be performed)	Knowledge		
8	35.	Do you know about all the diagnostic kits and Card based Immunoassay tests done in your lab?	Knowledge		
	36.	Do you know about the acceptable reference ranges of test results, example; Normal Values of	Knowledge		

		various parameters like; Blood Urea, creatinine, glucose levels etc.			
	37.	Do you know about the critical values of test results and alarming critical alerts?	Knowledge		
	38.	Do you feel the importance of displaying shelf life of inhouse prepared reagents, like Giemsa Stain for blood cell counting and morphology ; Zeil-Neelson stain for Acid fast Bacilli; Buffers for many other reagents, e.g. for biochemistry test?	Attitude		
	39.	Do you know about point of care testing?	Knowledge		
	40.	Do you think that higher education will improve your professional work quality?	Attitude		
	41.	Do you know that the clinical Specimens are required to be retained for some specific period even after successful completion of tests?	Knowledge		
	42.	Do you know how the clinical samples are stored during the retention period?	Knowledge		
	43.	Do you know how to dispose of various clinical samples after use /after completion of retention period as per prevailing/applicable biomedical waste management rule?	Knowledge		
	44.	Do you understand the concept of "segregation at source" in relation to the Bio Medical Waste Management?	Knowledge		
9	45.	Do you know about the concept of transcript check (Example-One should check and observe whether the rough data of results, the data entered into the lab register or on LIS and the report data entered into the final report format to be given to the patient are same)	Knowledge		
	46.	Do you know the standard units used in test reports of various parameters, like the units used in blood sugar, Hb, TLC, DLC, and platelets count etc.	Knowledge		
10	47.	Do you know about general (Chemical, Biological and non-biological like electrical safety, fire safety) and specific safety precautions in clinical laboratory like, how to deal with blood spill, exposure of your skin to acid or alkalis etc?	Knowledge		
	48.	Do you know what are the precautions required to deal with fire safety?	Knowledge		
	49.	Have you ever been trained/ participated in any fire safety training/ mock drill?	Knowledge		
	50.	Do you know which precautions are required to deal with electrical safety?	Knowledge		

	51.	Do you know that in the electrical fittings, proper earthing is important to avoid electric shocks from any metallic equipment and its damage?	Knowledge		
	52.	Do you feel that FIFO is not much useful in maintaining inventory for reagents?	Attitude		
	53.	Do you know about the laboratory hygiene and infection control practices / Policy in the laboratory?	Knowledge		
	54.	Do you think calibration and validation of equipment and the test procedures are important for proper test results?	Attitude		
	55.	Do you know what the biosafety equipment are?	Knowledge		
	56.	Do you know that the micropipettes or auto pipettes should be used in place of mouth pipetting?	Knowledge		
	57.	Do you know that the labelling dating, handle, store and dispose of chemicals, dyes, reagents, and solutions should be as per applicable legislations is mandatory?	Knowledge		
	58.	Do you know that every chemical accompanied by a document called as Master safety data sheet (MSDS), which explains "how that chemical can be used safely" explaining specifically the safety precautions specifically to all the requirements?	Knowledge		
	59.	Are you aware of that the burning type of syringe needle destroyers are not recommended these days? if not then why?	Knowledge		
	60.	Do you know that how does the mechanical syringe needle destroyers are safe to be used?	Knowledge		
	61.	Do you know the different type of disinfectants used in the laboratory for disinfection of floors, work benches, environmental disinfections and discarding jars?	Knowledge		
	62.	Do you know about Needle Stick Injuries?	Knowledge		
	63.	Do you feel that you have acquired all knowledge and skills for delivering your assigned work?	Attitude		
	64.	Do you know about the needle stick injury recording process?	Knowledge		
	65.	In the case of breakage of glass tubes containing clinical samples or some microbial suspension, inside the centrifuge machine, do you know how to handle this situation?	Knowledge		
	66.	Do you think, the application of quality control concept can improve the quality of laboratory test reports?	Attitude		
	67.	Do you know about the type of infection, that could be acquired during sample collection?	Knowledge		

	68.	Do you know about the precautions measure while working in the laboratory?	Knowledge		
	69.	Do you know that laboratory environment can be contaminated while working with clinical samples/ live organisms?	Knowledge		
	70.	Do you know the protocols could be followed in case of exposure to body fluids (as applicable)	Knowledge		
	71.	Do you know that hands are the most important vehicle for microbial transmission?	Knowledge		
	72.	Do you think, it is important for Medical Laboratory Technicians to respect the diversity, dignity, values and beliefs of patients /clients and colleagues, for delivering Laboratory Services?	Attitude		
	73.	Do you know that the proper handwashing with soap and water reduces hospital acquired infections by more than 80 %?	Knowledge		
	74.	Do you know under what circumstances alcohol-based hand sanitizer are recommended for use?	Knowledge		
	75.	Do you know that if you encounter any sharp cuts, then how will you respond?	Knowledge		
	76.	Do you feel preparation and display of SOPs for all the processes and equipment are waste of resources ?	Attitude		

C.2. Observational Tools (Skills)

Score Definition

A. None—No demonstrated skills at all/does not perform the task(s) completely
B. Limited Demonstrated very limited strengths/skills in this area
C. Some—Demonstrated some ability/skills in this area.
D. Strong—Demonstrated strong skills/strength in this area.
E. Excellent—Demonstrated excellent skills/strength in this area.
F. Not applicable
G. Don't know- Not even heard about that skill
H. Skill limitation is clearly related to resource limitations

Domain Code	S. N.	Observation Points	Means of Verification	Response (Score)
1	1.	Responsibilities towards Patients and their attendants	Listens to patient's ideas and concerns. Doesn't rush patient and doesn't take too much time; Maintains speaking in low pitch voice; Provides documentation that is accurate and complete	
	2.	Compliances towards legislation that applies for Medical Laboratory Services in that facility	Documentation in relation to ; Notifiable Diseases, Biomedical Waste Management Rules 2016 , Minimum Standards of Medical Diagnostic Laboratories as per notifications	

			from Government bodies from time to time like; Clinical Establishment Act	
2	3.	Corrective Action and Preventive Action (CA-PA)?	Documentation in terms of<br>-Incident/Accident Log/Record<br>-CA-PA Log/ Record	
	4.	Simple calculations	Final Result of Test like ; A. Conjugated, Non conjugated and Total Bilirubin B. Calculation of Prothrombin Index (PTI)	
	5.	Calibration	Internal Calibration of Commonly used equipment e.g., Weighing Balance; and Micro Pipettes etc and **check their calibration records**	
	6.	Efficacy Testing	The efficacy of any equipment may vary time to time because of many technical or mechanical reasons, hence it needs to be checked frequently that the concerned equipment is working as per required efficacy for example -Hot Air Oven; Autoclave or any other sterilizer; Incubators; pH meters, any other instruments/ equipment used in the laboratory.<br>If done, **check the records.**	
	7.	First in First Out (FI-FO or First Entry and first out (FE-FO)<br>With special reference to Shelf life/ Expiry Date	Whether First in First out concept is followed?<br><br>Verify the following?<br><br>1. Check the Inventory for any expired materials kept in the stock including perishable items in refrigerator<br>2. Match the stock the with the stock register or digital data., if any , as applied therein<br>3. Check that the recently procured items are kept serially behind the previously procured items<br>4. Whether the stocks are placed as per their hazardous category requirements for example A. Alcohols are stored separately under lock B. Acids are always kept separately on floor only.	

5	8.	Use of positive and negative control for tests	Availability of Positive and negative control reagents<br><br>Check the use log of controls samples or reagents	
	9.	Labelling of equipment/instrument	Respective unique I.D.s,<br>date of purchase,<br>date of installation,<br>date of putting into service,<br>date of the last calibration, and<br>Name and contact of address mechanic whom to inform in case of emergency	
6	10.	Test requisition form	Test requisition form: Name, age, sex, Patient ID, Sample and Test required, Clinical diagnosis, Previous investigation result if any, Clinician Identity, Date, Box for Sampling date and time. Signature of recommending doctor, name of Ward/OPD/Clinic	
	11.	Sample Collection Instructions	To observe whether he/she gives the instructions to the patient (how to collect the sample) for example;<br>1. how to collect a urine sample for microbial culture from a male or a female patient?<br>2. How to collect urine sample for routine examination and for 24-hour urine protein analysis<br>3. How to collect throat sample from suspected cases of diphtheria.<br><br>Instructions to patients to collect sputum sample from suspected pneumonia or Tuberculosis case	
7	12.	Specimen suitability	Should explain at least one example like; Renal Function Test and Liver Function Tests require clear serum of the patient blood but if the blood sample is haemolysed it is not suitable for these tests to be performed.	
8	13.	Manual Counting of Blood cells	To observe whether the professional is able to perform the manual blood cell counting like, TLC, DLC, Platelets count etc.	
	14.	Manual Biochemistry Tests	To observe whether the professional is able to perform manually the common biochemistry tests example-Blood Sugar, Urea, Creatinine, electrolytes, Urine sugar, urine proteins, pregnancy tests etc.	

	15.	Microscopic Analysis of Clinical samples for cell morphology and counting	To observe whether the professional is able to perform for various clinical samples for cell morphology and counting. Example- Urine and other body fluid microscopy	
	16.	Reference Values	To observe whether reference values of each test results have been displayed in the laboratory or on the test requisition form	
9	17.	Result Recording and Reporting	Observe the recording of results (Rough and final) - Sample Identity (Transcript Check)	
	18.	Positive and negative control Results to be matched with their known values	Observation – Before final report recording, does professional match the positive and negative control values with their actual and known values respectively.	
	19.	Laboratory data Safety in reference to notifiable diseases like HIV, Tuberculosis etc.	Observe the accessibility to the laboratory records by any un-authorized persons.<br><br>Records kept under lock.<br><br>Maintains the charge handover takeover records, while during shift change or while leaving for or joining back from leave or holidays.	
10	20.	General Safety precautions in medical laboratory	To demonstrate dealing with major and minor blood spills in and outside the equipment.<br><br>Observe his/ her laboratory for fire safety protocol which has been validated within year.<br><br>Observe the documentation related to fire safety training/ mock drill.<br><br>Observe for any the naked electrical connection /wire	
	21.	Laboratory Safety	To observe what type of pipetting devices are being used in the laboratory.	
	22.	Chemical Safety	To observe if any MSDS has been recorded in the laboratory documentation.<br><br>Observe that the chemicals have been kept following their hazardous category.	
	23.	laboratory hygiene and infection control practices / Policy	Whether the laboratory has any written / documented and displayed infection control policy.<br><br>If yes, does the professional follow that policy?	

	24.	Disposal of used Syringes and needles	To observe how does the needles and syringes are being disposed of?<br><br>Whether the laboratory has dedicated functional syringe needle destroyer<br><br>What type of syringe needle destroyer you have: burning type and mechanical cutting based?	
	25.	Hand hygiene	To observe whether the professional follows the proper hand hygiene protocol as per WHO Protocol.	
	26.	Sharp Cuts	To observe the register/ documented record in which sharp cuts are being recorded.	

C.3. Mini Clinical Laboratory Skills

Score Definition

A. None—No demonstrated skills at all/does not perform the task(s) completely
B. Limited Demonstrated very limited strengths/skills in this area
C. Some—Demonstrated some ability/skills in this area.
D. Strong—Demonstrated strong skills/strength in this area.
E. Excellent—Demonstrated excellent skills/strength in this area.
F. Not applicable
G. Don't know- Not even heard about that skill
H. Skill limitation is clearly related to resource limitations

Dom.	S.N.	Clinical Evaluation Points	Means of Verification	Response (Score)	Remarks
6	3	Demonstration of Sample collection tools	Whether he/she is able to demonstrate sample collection tool like; sterile syringe and needle, lancet, cotton /rayon swab sticks, suitable anticoagulants, and sample collection containers for the samples, which are routinely processed.		

	5	Use of anticoagulants for specific tests	Proper and suitable coagulants in sample collection vials as per the test requirement. For example; requirement of serum, plasma and whole blood-for hemogram is done in EDTA (Ethylenediaminetetraacetic acid) Containing sample vials; and for blood sugar estimation, sample should be collected in sodium fluoride containing vial, and the general biochemistry test, no anticoagulants should be in the vial.		
	8	Staining Procedures	Able to demonstrate the principles, reagents used and procedures of various staining methods used in his/her laboratory.<br>Example 1. Preparation of thin and thick smear of blood for detection of malarial parasite.<br><br>Example 2. Various staining procedures available in the lab for detection of malarial parasite.<br><br>Example 3. Preparation of blood film on glass slide for differential leucocyte count (DLC) and cell morphology.		
	9	Microscopy	Should be able to focus the smear efficiently under the microscope and observe for abnormalities therein in relation to the expectation of the test. For example- can he find malarial parasite present in the blood film under the microscope.		

C.4. Case Study

Dom	Sr. No.	Case	Response (Satisfactory/ Unsatisfactory)	Remarks
4	**1**	**Able to organize work to accommodate valid priorities- for example-**<br>Last month, one patient Rima landed your hospital with gestational complications. Medical Officer of your facility identified this as critical case that		

		needs immediate lower abdomen Caesarean section. The doctor on duty sent her blood sample to your laboratory for D-dimer. At the same time, a patient comes to doctor as an emergency case. Symptomatically, doctor wishes to rule out the diabetes and sends the sample to laboratory for blood sugar testing. Under the circumstances, to which sample you will prioritize to process.		
	2	Able to demonstrate effective problem solving/trouble-shooting strategies and initiates the appropriate follow-up **for example-**<br>Suppose you have received one sample from the physician who wishes to know whether or not the sample contains any gram positive or gram-negative microbe in it. Per chance you found that your gram stain kit is out of stock. On enquiring from the store, the storekeeper informed that except gram staining kit everything is available in the store. Under the circumstances, how would you be able to perform gram staining and help the clinician in making diagnosis of concerned patient?<br><br>By this case study, the assessor wishes to know whether the laboratory technician will do efforts to perform the test using some alternative method, if the standard method diagnostic kit or reagents are not available per chance.		As the store-keeper clarified that he has everything except gram staining kit, the professional should get the chemicals (Crystal Violet, Safranin, Acetone, and Alcohol) , issued from the store, which are required to prepare the gram staining reagents manually and perform the procedure.<br><br>Some more example on trouble shouting in a basic clinical lab
5	3	Risk assessment for any processes or in using instruments/ equipment **for example-**<br><br>Suppose you wish to centrifuge the blood samples to separate serum. What type of risks, may be there, which need to be assessed so that centrifugation is expected to be done as risk free?		He will assess that -1. the machine is properly working<br>2. There is nothing unrequired inside the chamber like; broken glass, any type of spilled fluid etc.<br>3. The shock absorbing cushions are present in all the test tube cups.<br>4. The test tubes and cups are properly balanced.

Formatted: Font color: Red

				5. Ensure that the lid is properly locked, before starting the machine.<br>6. Increases the speed slowly not with the sudden jerk.<br>7. After completion of centrifugation time and switching it off he waits till the rotor stops automatically.<br>8. Whether he waits minimum for 5-10 Minutes allowing the aerosols to settle if any before opening the lid.
8	4	Critical Alerts:<br>**for example-**<br>A sixty-year-old patient approaches the emergency with some problems and symptomatically the physician expects it as a hypoglycemic case and sends his blood sample for blood sugar testing. You performed the test and found that his blood glucose level is 40 mg/dl. What will your further action?		Since the blood glucose level of the patient is very low and patient may enter into coma phase, considering it critical the laboratory professional should convey the report immediately to the physician, as a critical alert so that the patient may be saved.

**********************************The End**
