# Supplementary material for: Developing a competency assessment framework for medical laboratory technologists in primary healthcare settings in India
Source: PLoS One. 2024 Apr 1;19(4):e0294939. doi: 10.1371/journal.pone.0294939 (PMC10984544; doi:10.1371/journal.pone.0294939)
Supplement: S5 File — (PDF) [file pone.0294939.s005.pdf]

Competency Assessment Tool- Medical Laboratory Technician
ଦକ୍ଷତା ମୂଲ୍ୟାୟନ ପ୍ରଶ୍ନାବଳୀ - ମେଡିକାଲ୍ ଲାବୋରେଟୋରୀ ଟେକ୍ନିସିଆନ୍

A. General Information:
କ. ସାଧାରଣ ସୂଚନା:

A1. Date & Time:<br>କ୧. ଦିନାଙ୍କ ଏବଂ ସମୟ:	A4. Designation:<br>କ୪. ପଦବୀ:
A2. Age:<br>କ୨. ବୟସ:	A5. Health Facility:<br>କ୫. ସ୍ୱାସ୍ଥ୍ୟ ସେବା ସ୍ଥଳ:
A3. Gender:<br>କ୩. ଲିଙ୍ଗ:	A6. District:<br>କ୬. ଜିଲ୍ଲା:

A7. Education & In Service Training:<br>କ୭. ଶିକ୍ଷା ଏବଂ ସେବା କାଳୀନ ପ୍ରଶିକ୍ଷଣ:		High School<br>ମାଧ୍ୟମିକ ଶିକ୍ଷା	Intermediate<br>ଇଣ୍ଟରମିଡିଏଟ୍	Technical Education<br>ବୈଷୟିକ ଶିକ୍ଷା
	Place<br>ସ୍ଥାନ			
	Year of Completion<br>ସମ୍ପୂର୍ଣ୍ଣ ହେବାର ବର୍ଷ			

A8. Postings details<br>କ୮. କାର୍ଯ୍ୟ ସ୍ଥଳ ବିବରଣୀ	Place of Posting<br>କାର୍ଯ୍ୟ ସ୍ଥଳର ସ୍ଥାନ	District<br>ଜିଲ୍ଲା	Period (From-to)<br>ଅବଧି (ଆରମ୍ଭ- ଶେଷ)	Regular/ Contractual<br>ନିୟମିତ/ ଠିକା	Reason(s) for transfer<br>ବଦଳି ହେବାର କାରଣ
1<br>୧					
2<br>୨					
3<br>୩					
4<br>୪					
5<br>୫					

B. General Questions (Self Appraisal):
ଖ. ସାଧାରଣ ପ୍ରଶ୍ନାବଳୀ (ନିଜେ ମୂଲ୍ୟାୟନ କରିବେ):

B1. Do you know, how many tests are provisioned for your facility?<br><br>ଖ୧. ଆପଣ ଜାଣିଛନ୍ତି କି ଆପଣଙ୍କ ସ୍ୱାସ୍ଥ୍ୟ ସେବା ସ୍ଥଳରେ କେତେ ଗୋଟି ପରୀକ୍ଷା ପାଇଁ ବ୍ୟବସ୍ଥା ଉପଲବ୍ଧ ଅଛି?
B2. How many laboratory tests are being done in this facility? Can you name those tests?<br><br>ଖ୨. ଏହି କେନ୍ଦ୍ରରେ କେତୋଟି ଲାବୋରେଟୋରୀ ପରୀକ୍ଷା କରାଯାଉଛି? ଆପଣ ସେହି ପରୀକ୍ଷା ଗୁଡିକର ନାମ କହିପାରିବେ କି?
B3. In your opinion, how competent you are to perform your day-to-day activities?<br><br>ଖ୩. ଆପଣଙ୍କ ମତରେ ଆପଣ ନିଜର ଦୈନିକ କାର୍ଯ୍ୟରେ କେତେ ଦକ୍ଷ ଅଟନ୍ତି ?
B4. In a scale of 1-5, how would you rate your overall clinical / technical knowledge & skills to perform your duty?<br><br>(1-very little, 2- less, 3- adequate, 4- more than needed, 5-exceptional)<br><br>ଖ୪. ୧- ୫ ର ସ୍କେଲ୍ ରେ , ଆପଣ ନିଜର କ୍ଲିନିକାଲ୍/ ବୈଷୟିକ ଜ୍ଞାନ ଏବଂ ଦକ୍ଷତାକୁ କେତେ ମୂଲ୍ୟ ଦେବେ?<br><br>(୧ – ବହୁତ କମ, ୨- କମ, ୩- ଯଥେଷ୍ଟ, ୪- ଆବଶ୍ୟକ ରୁ ଅଧିକ, ୫- ଅସାଧାରଣ)
B5. What competencies you have, those are of use the most in carrying out the assigned duties?<br><br>ଖ୫. ଆପଣଙ୍କ ନିକଟରେ କେଉଁ ଦକ୍ଷତା ଅଛି ଯାହା ଆପଣଙ୍କୁ ଦିଆଯାଇଥିବା କାର୍ଯ୍ୟକୁ ଠିକ ଭାବରେ ପୁରଣ କରିବା ପାଇଁ ଅଧିକ ଉପଯୋଗୀ ଅଟେ?
B6. Do you feel that you have additional skill sets to perform tasks, other than the assigned duties to you, competently?<br><br>ଖ୬. ଆପଣ ଭାବୁଛନ୍ତି କି, ଦିଆଯାଇଥିବା କାର୍ଯ୍ୟ ଛଡା ଅନ୍ୟ କାର୍ଯ୍ୟକୁ ଯୋଗ୍ୟତାର ସହ ପୁରଣ କରିବାର ଅତିରିକ୍ତ ଦକ୍ଷତା ଆପଣଙ୍କ ନିକଟରେ ଅଛି ?
B7. In your opinion, what other competencies do you need to perform better in your job?<br><br>ଖ୭. ଆପଣଙ୍କ ମତରେ କାର୍ଯ୍ୟକୁ ଅଧିକ ଭଲ ଭାବରେ କରିବାରେ ଆପଣଙ୍କୁ ଅନ୍ୟ କେଉଁ ଦକ୍ଷତାର ଆବଶ୍ୟକତା ଅଛି?
B8. What all trainings have been given to you to make yourself competent to carry out the assigned duties?<br><br>ଖ୮. ଆପଣଙ୍କୁ ଦିଆଯାଇଥିବା କାର୍ଯ୍ୟକୁ ଠିକ ଭାବରେ କରିବା ପାଇଁ କ'ଣ କ'ଣ ପ୍ରଶିକ୍ଷଣ ଦିଆଯାଇଅଛି?

B9. Is there any mechanism to provide training/ orientation before introduction of any new sample collection tool?<br><br>ଖ୯. କୌଣସି ନୂତନ ନମୁନା ସଂଗ୍ରହ ପଦ୍ଧତି ବ୍ୟବହାର ହେବା ପୂର୍ବରୁ ପ୍ରଶିକ୍ଷଣ ପ୍ରଦାନ କରିବାର ବ୍ୟବସ୍ଥା ଅଛି କି?
B10. What additional trainings are required for you to carry out your duties, efficiently?<br><br>ଖ୧୦. କାର୍ଯ୍ୟକୁ ଅଧିକ ଭଲ ଭାବରେ କରିବାରେ ଆପଣଙ୍କୁ କେଉଁ ଅତିରିକ୍ତ ପ୍ରଶିକ୍ଷଣର ଆବଶ୍ୟକତା ରହିଛି?
B11. Do you feel that you are performing well or at least, at par with your expectations in this job?<br><br>If yes/no, then why do you feel so?<br><br>ଖ୧୧. ଆପଣ ଭାବୁଛନ୍ତି କି ଆପଣ ଏହି କାର୍ଯ୍ୟକୁ ସନ୍ତୋଷଜନକ କିମ୍ବା ଅତି କମରେ ଆପଣଙ୍କ ଆଶାନୁଗତ ସ୍ତରରେ ତୁଲାଇ ପାରୁଛନ୍ତି ?<br><br>ଯଦି ହଁ/ନା, ତେବେ ଆପଣ ଏପରି କାହିଁକି ଭାବୁଛନ୍ତି ?
B12. How would you describe your working conditions, here at this facility?<br><br>ଖ୧୨. ଏହି ସ୍ୱାସ୍ଥ୍ୟ ସେବା ସ୍ଥଳରେ ଆପଣଙ୍କ କାର୍ଯ୍ୟ ପରିବେଶକୁ ଆପଣ କିପରି ବର୍ଣ୍ଣନା କରିବେ?
B13. What are the working conditions, which enables / disables you to carry out your duties?<br><br>ଖ୧୩. କେଉଁ ଭଳି କାର୍ଯ୍ୟ ସ୍ଥିତି ଅଛି ଯାହା ଆପଣଙ୍କୁ ଆପଣଙ୍କ କାର୍ଯ୍ୟ ତୁଲାଇବାରେ ସୁବିଧା/ ଅସୁବିଧା କରୁଛି?
B 14. What is the supply chain mechanism to ensure the availability of adequate diagnostic supplies and consumables, equipment in your facility/ laboratory?<br><br>ଖ୧୪. ଆପଣଙ୍କ ସ୍ୱାସ୍ଥ୍ୟ ସେବା ସ୍ଥଳ/ ଲାବୋରେଟୋରୀରେ ଯଥେଷ୍ଟ ଡାଇଗ୍ନୋସ୍ଟିକ୍ ସପ୍ଲାଇ ଏବଂ କନ୍ଜ୍ୟୁମେବଲ୍ସର ଉପଲବ୍ଧତା ସୁନିଶ୍ଚିତ କରିବା ପାଇଁ ଯୋଗାଣ ବ୍ୟବସ୍ଥା କ'ଣ ଅଟେ ?

C. Competencies Domains[1]

ଗ. ଦକ୍ଷତା କ୍ଷେତ୍ର1

Competencies Domains<br>ଦକ୍ଷତା କ୍ଷେତ୍ର	Codes<br>କୋଡ୍
**Human Values and Professional Ethics**<br>ମାନବିକ ମୂଲ୍ୟ ଏବଂ ବୃତ୍ତିଗତ ଆଚାର ନୀତି	**1**<br>୧
**Quality Management**<br>କ୍ୱାଲିଟି ମ୍ୟାନେଜ୍ମେଣ୍ଟ୍	**2**<br>୨
**Communication and Interaction**<br>କମ୍ୟୁନିକେସନ୍ ଏବଂ ଇଣ୍ଟର୍ -ଆକ୍ସନ୍	**3**<br>୩
**Critical Thinking**<br>କ୍ରିଟିକାଲ୍ ଥିଙ୍କିଙ୍ଗ୍	**4**<br>୪
**Equipment Instruments and Consumables**<br>ଇକ୍ୱିପ୍ମେଣ୍ଟ୍, ଇନ୍ଷ୍ଟ୍ରୁମେଣ୍ଟ୍ ଏବଂ କନ୍ଜ୍ୟୁମେବଲ୍ସ	**5**<br>୫
**Test Requisition Data, Sample collection and transportation**<br>ଟେଷ୍ଟ ରିକ୍ୱିଜିସନ୍ ଡାଟା, ନମୁନା ସଂଗ୍ରହ ଏବଂ ନମୁନା ପରିବହନ	**6**<br>୬
**Specimen Preparation**<br>ସ୍ପେସିମେନ୍ ପ୍ରସ୍ତୁତି	**7**<br>୭
**Assessment & Analysis**<br>ଆସେସ୍ମେଣ୍ଟ୍ ଏବଂ ଆନାଲିସିସ୍	**8**<br>୮
**Recording and Reporting**<br>ରେକର୍ଡିଂ ଏବଂ ରିପୋର୍ଟିଂ	**9**<br>୯
**Laboratory Safety and Infection Control**<br>ଲାବୋରେଟୋରୀ ସୁରକ୍ଷା ଏବଂ ସଂକ୍ରମଣ ନିୟନ୍ତ୍ରଣ	**10**<br>୧୦

This tool is meant to assess the competencies of Medical Laboratory Technicians in terms of **Knowledge[2]**, **Skills[3]** and **Attitudes[4]** through **Checklist**, **Direct Observation of Procedural skills**, **Mini Clinical Laboratory Evaluation**.

ଏହି ଟୁଲ୍ ର ଉଦ୍ଦେଶ୍ୟ ମେଡିକାଲ ଲାବୋରେଟୋରୀ ଟେକ୍ନିସିଆନ୍ ଙ୍କ ଜ୍ଞାନ[2], କୌଶଳ[3] ଏବଂ ମନୋଭାବ [4] କୁ ଚେକଲିଷ୍ଟ, ପ୍ରକ୍ରିୟାଗତ କୌଶଳର ପ୍ରତ୍ୟକ୍ଷ ଅନୁଧ୍ୟାନ, କ୍ଷୁଦ୍ର କ୍ଲିନିକାଲ୍ ଲାବୋରେଟୋରୀ ମୂଲ୍ୟାଙ୍କନ ଦ୍ୱାରା ଆକଳନ କରିବା ଅଟେ |

[1] Broadest Category of Competencies
[୧] ଯୋଗ୍ୟତା ଗୁଡିକର ବିସ୍ତୃତ ବର୍ଗୀକରଣ

[2] Concepts and Theories
[୨] କନ୍ସେପ୍ଟ ଏବଂ ଥିଓରୀ

[3] Use of techniques to integrate knowledge into practice
[୩] ଅଭ୍ୟାସରେ ଜ୍ଞାନର ଏକୀକରଣ ପାଇଁ କୌଶଳର ବ୍ୟବହାର

[4] A person's feelings, values, and beliefs, which influence their behaviour and the performance of tasks
[୪] କୌଣସି ବ୍ୟକ୍ତିଙ୍କର ଭାବନା, ମୂଲ୍ୟ ଏବଂ ବିଶ୍ୱାସ ଯାହା ତାଙ୍କର ବ୍ୟବହାର ଏବଂ କାର୍ଯ୍ୟ ପ୍ରଦର୍ଶନକୁ ପ୍ରଭାବିତ କରେ

C.1. Questionnaire (Knowledge and Attitude)

ଗ ୧. ପ୍ରଶ୍ନାବଳୀ (ଜ୍ଞାନ ଏବଂ ମନୋଭାବ)

Domain Code ଡୋମେନ୍ କୋଡ୍	S. N. କ୍ରମିକ ସଂଖ୍ୟା	Points ପଏଣ୍ଟ	Knowledge/ Attitude/ Skill ଜ୍ଞାନ/ ମନୋଭାବ / କୌଶଳ	Response (Yes/No) ପ୍ରତିକ୍ରିୟା (ହଁ/ ନା)	Remarks ଟିପ୍ପଣୀ
1	1.	Do you understand the terms “human values and ethics”?<br>ଆପଣ ଏହି ଉକ୍ତି "ମାନବିକ ମୂଲ୍ୟ ଏବଂ ଆଚାର ନୀତି" ର ଅର୍ଥ ବୁଝିଛନ୍ତି?	Knowledge<br>ଜ୍ଞାନ		
	2.	In case of sharp cuts, do you feel the need to report to medical officer/ physician nearby?<br><br>ଧାରଜନିତ କଟା କ୍ଷତ ହେବା କ୍ଷେତ୍ରରେ ଆପଣ ନିକଟସ୍ଥ ମେଡିକାଲ୍ ଅଫିସର୍ / ଫିଜିସିଆନ୍ ଙ୍କୁ ଜଣେଇବା ଆବଶ୍ୟକ ମନେ କରନ୍ତି କି?	Attitude<br><br>ମନୋଭାବ		
	3.	Do you feel that handwashing is not much important particularly before and after contact with the patient or patient sample or any other activities likely to cause contamination?<br><br>ଆପଣ ଭାବନ୍ତି କି, ବିଶେଷ କରି ରୋଗୀଙ୍କ ସମ୍ପର୍କରେ ଆସିବା କିମ୍ବା ରୋଗୀଙ୍କ ନମୁନା କିମ୍ବା ଅନ୍ୟ କୌଣସି ଗତିବିଧି ଯାହା କଣ୍ଟାମିନେସନ୍ କରାଇ ପାରେ, ଏହା ପୂର୍ବରୁ ଏବଂ ପରେ ହାତ ଧୋଇବା ନିହାତି ଆବଶ୍ୟକ ଅଟେ ନାହିଁ?	Attitude<br><br>ମନୋଭାବ		
	4.	Do you know what are the ways you can adopt (Addressing the complains and feedbacks from Patient, Physician, Staff and learnings from accidents/ Incidents), for continual improvement in laboratory Services?<br><br>ଆପଣ ଜାଣିଛନ୍ତି କି ଲାବୋରେଟୋରୀ ସେବା ଗୁଡିକର ନିରନ୍ତର ସୁଧାର କରିବା ପାଇଁ ପନ୍ଥା ଗୁଡିକ କଣ ଅଟନ୍ତି (ରୋଗୀ, ଫିଜିସିଆନ୍, କର୍ମଚାରୀ ମାନଙ୍କ ଠାରୁ ମିଳିଥିବା ଅଭିଯୋଗ ଏବଂ ପ୍ରତିକ୍ରିୟାକୁ ସମ୍ବୋଧିତ କରି ଏବଂ ଦୁର୍ଘଟଣା/ ଘଟଣା ଗୁଡ଼ିକରୁ ମିଳିଥିବା ଶିକ୍ଷା)	Knowledge<br><br>ଜ୍ଞାନ		
	5.	In case of needle stick injury, do you contact the physician for further action?	Attitude		

		ନୀଡିଲ୍ ଷ୍ଟିକ୍ ଆଘାତ ହେବା କ୍ଷେତ୍ରରେ ଆପଣ ପରବର୍ତ୍ତୀ ପଦକ୍ଷେପ ପାଇଁ ଫିଜିସିଆନ୍ ଙ୍କୁ ସମ୍ପର୍କ କରିବେ କି ?	ମନୋଭାବ		
	6.	Do you know what are the legislations/ regulatory bodies that governs medical laboratory in your facilities?<br><br>ଆପଣ ଆପଣଙ୍କ ମେଡିକାଲ ଲାବୋରେଟୋରୀ କେନ୍ଦ୍ରରେ ନିୟାମକ/ ନିୟନ୍ତ୍ରଣ ସଂସ୍ଥା ମାନଙ୍କ ବିଷୟରେ ଜାଣନ୍ତି କି?	Knowledge<br><br>ଜ୍ଞାନ		
	7.	Do you have any idea, that how could you acquire those knowledge and skills?<br><br>ଆପଣଙ୍କର କୌଣସି ବିଚାର ଅଛି କି ଆପଣ କିପରି ସେହି ଜ୍ଞାନ ଏବଂ କୌଶଳକୁ ଆହରଣ କରିପାରିବେ?	Knowledge<br><br>ଜ୍ଞାନ		
	8.	Do you know about Informed Consent and its use including patients right to refuse?<br><br>କ'ଣ ଆପଣ ସୂଚିତ ସମ୍ମତି ଏବଂ ଏହାର ବ୍ୟବହାର ସହ ରୋଗୀଙ୍କର ଅନିଚ୍ଛା ପ୍ରକାଶ କରିବାର ଅଧିକାର ବିଷୟରେ ଅବଗତ?	Knowledge<br><br>ଜ୍ଞାନ		
	9.	If there is an infection control policy, do you feel it is not worthful to follow?<br><br>ଯଦି ଏକ ସଂକ୍ରମଣ ନିୟନ୍ତ୍ରଣ ନୀତି ଥାଏ, ଆପଣ ଭାବନ୍ତି କି ଏହାର ଅନୁପାଳନ କରିବା ଫଳପ୍ରଦ ଅଟେ ନାହିଁ?	Attitude<br><br>ମନୋଭାବ		
2	10.	Do you understand the term "Quality Control including internal and external QC"?<br><br>କ'ଣ ଆପଣ ଏହି ଉକ୍ତି "କ୍ୱାଲିଟି କଣ୍ଟ୍ରୋଲ୍ ସହ ଇଣ୍ଟର୍ନାଲ୍ ଏବଂ ଏକ୍ସଟର୍ନାଲ୍ QC (କ୍ୱାଲିଟି କଣ୍ଟ୍ରୋଲ୍)" କୁ ବୁଝିଛନ୍ତି?	Knowledge<br><br>ଜ୍ଞାନ		
	11.	Do you maintain and display turnaround time (TAT) for all the tests done in your laboratory specific to each test?<br><br>କ'ଣ ଆପଣ ଅଣପଣଙ୍କ ଲାବୋରେଟୋରୀରେ କରାଯାଉଥିବା ପ୍ରତ୍ୟେକ ପରୀକ୍ଷାର ଟର୍ଣ୍ଣଆରାଉଣ୍ଡ୍ ଟାଇମ୍ (TAT) କୁ ବଜାୟ ରଖିବା ସହ ଡିସ୍-ପ୍ଲେ କରନ୍ତି?	Knowledge<br><br>ଜ୍ଞାନ		
	12.	Do you wear masks and gloves while working in the laboratory?<br><br>କଣ ଆପଣ ଲାବୋରଟୋରୀରେ କାର୍ଯ୍ୟ କରିବା ସମୟରେ ମାସ୍କ୍ ଏବଂ ଗ୍ଲୋବ୍ସ ପରିଧାନ କରନ୍ତି କି ?	Attitude<br><br>ମନୋଭାବ		
	13.	Do you Know the concept of Corrective Action and Preventive Action (CA-PA)?	Knowledge		

		କ'ଣ ଆପଣ କରେକ୍ଟିଭ୍ ଆକ୍ସନ୍ ଏବଂ ପ୍ରିଭେଣ୍ଟିଭ୍ ଆକ୍ସନ୍ (CA - PA)ର କନ୍ସେପ୍ଟ ବିଷୟରେ ଅବଗତ ?	ଜ୍ଞାନ		
	14.	Do you know about the protocols defined in the quality policies, process, and procedure manuals, if any ?<br><br>କ'ଣ ଆପଣ ଗୁଣବତ୍ତା ନୀତି , ପ୍ରକ୍ରିୟା ଏବଂ ପ୍ରକ୍ରିୟା ମାନୁଆଲ୍ ରେ ପରିଭାଷିତ ପ୍ରୋଟୋକଲ୍ ଯଦି କିଛି ଥାଏ, ତାହା ବିଷୟରେ ଅବଗତ କି?	Knowledge<br><br>ଜ୍ଞାନ		
	15.	Do you feel that the continuing education and training programs could be helpful, in improving your performance, work environment and career progression?<br><br>ଆପଣ ଅନୁଭବ କରନ୍ତି କି ଶିକ୍ଷା ଏବଂ ପ୍ରଶିକ୍ଷଣ କାର୍ଯ୍ୟକ୍ରମ ଜାରି ରଖିବା ଆପଣଙ୍କ ପ୍ରଦର୍ଶନ, କାର୍ଯ୍ୟ ପରିବେଶ ଏବଂ କ୍ୟାରିୟର ପ୍ରଗତି ସୁଧାରିବାରେ ସାହାଯ୍ୟକାରୀ ହୋଇ ପାରିବ?	Attitude<br><br>ମନୋଭାବ		
	16.	Do you know about Calibration and preventive maintenance of equipment?<br><br>କ'ଣ ଆପଣ ଉପକରଣ ଗୁଡିକର କ୍ୟାଲିବରେସନ୍ ଏବଂ ପ୍ରିଭେଣ୍ଟିଭ୍ ମେଣ୍ଟେନାନ୍ସ ବିଷୟରେ ଅବଗତ କି?	Knowledge<br><br>ଜ୍ଞାନ		
	17.	Do you feel the protocols as defined in the quality policies, processes and procedure manuals are not useful in ensuring quality laboratory Services?<br><br>ଆପଣ ଭାବନ୍ତି କି ପ୍ରଟୋକଲ୍ ରେ ପ୍ରଦତ୍ତ ଗୁଣବତ୍ତା ନୀତି, ପ୍ରକ୍ରିୟା ଏବଂ ପ୍ରକ୍ରିୟା ନିୟମାବଳୀ ଗୁଣଯୁକ୍ତ ଲାବୋରେଟୋରୀ ସେବା ସୁନିଶ୍ଚିତ କରିବାରେ ଉପଯୋଗୀ ନୁହନ୍ତି?	Attitude<br><br>ମନୋଭାବ		
	18.	Do you know that the equipment should be tested in routine for their efficacy?<br><br>ଆପଣ ଜାଣନ୍ତି କି ଉପକରଣ ଗୁଡିକୁ ସେମାନଙ୍କ ସଠିକ ପ୍ରଭାବ ପାଇଁ ନିୟମିତ ତଦାରଖର ଆବଶ୍ୟକତା ଅଛି?	Knowledge<br><br>ଜ୍ଞାନ		
	19.	Do you know about First in and First Out (FIFO)?<br><br>କ'ଣ ଆପଣ ଫାର୍ଷ୍ଟ ଇନ୍ ଏବଂ ଫାର୍ଷ୍ଟ ଆଉଟ୍ (FIFO) ବିଷୟରେ ଜାଣିଛନ୍ତି କି?	Knowledge<br><br>ଜ୍ଞାନ		
	20.	Have you ever considered, proper earthing in electrical circuit is important to avoid electric shocks from any metallic equipment and its damage?	Attitude		

		ଆପଣ କେବେ ବିଚାର କରିଛନ୍ତି କି କୌଣସି ଧାତବ ଉପକରଣ ଏବଂ ଏହାର କ୍ଷୟ ରୁ ହେଉଥିବା ବୈଦ୍ୟୁତିକ ଆଘାତ କୁ ଏଡାଇବା ପାଇଁ ବୈଦ୍ୟୁତିକ ସଂଯୋଗ ଗୁଡିକର ଉପଯୁକ୍ତ ଆର୍ଥିଂ କରାଇବା ଆବଶ୍ୟକ ଅଟେ?	ମନୋଭାବ		
4	21.	Do you know about higher education in your profession?<br><br>କ'ଣ ଆପଣ ନିଜ କର୍ମ କ୍ଷେତ୍ରରେ ଉଚ୍ଚତର ଶିକ୍ଷା ବିଷୟରେ ଅବଗତ ଅଟନ୍ତି କି?	Knowledge<br><br>ଜ୍ଞାନ		
	22.	In case of emergency or physical inability to take patient to the laboratory, would you like to go at sight for possible laboratory tests?<br><br>ଜରୁରୀକାଳୀନ ପରିସ୍ଥିତିରେ କିମ୍ବା ରୋଗୀଙ୍କୁ ଲାବୋରଟୋରୀକୁ ନେବାରେ ଶାରୀରିକ ଅକ୍ଷମତା କ୍ଷେତ୍ରରେ କ'ଣ ଆପଣ ସମ୍ଭାବ୍ୟ ଲାବୋରେଟୋରୀ ପରୀକ୍ଷା ଗୁଡିକ ପାଇଁ ସେହି ସ୍ଥାନକୁ ଯିବାକୁ ଚାହିଁବେ କି?	Attitude<br><br>ମନୋଭାବ		
5	23.	Do you know the importance of Shelf life of reagents, chemicals and diagnostic kits etc.?<br><br>କ'ଣ ଆପଣ ରିଏଜେଣ୍ଟ, ରାସାୟନିକ ପଦାର୍ଥ ଏବଂ ଡାଇଗ୍ନୋସ୍ଟିକ୍ କୀଟ୍ ଇତ୍ୟାଦି ମାନଙ୍କ ଶେଲ୍ଫ ଲାଇଫ୍ ର ମହତ୍ୱ ବୁଝନ୍ତି?	Knowledge<br><br>ଜ୍ଞାନ		
	24.	In case you observe any test results in critical range (Life threatening), would you like to inform the clinician immediately, by taking extra efforts?<br><br>ଯଦି ଆପଣ ଅନୁଧ୍ୟାନ କରନ୍ତି କି କୌଣସି ପରୀକ୍ଷାର ପରିଣାମ ବିପଦଜନକ ସ୍ତରରେ (ଜୀବନକୁ ବିପଦ) ଅଛି, କ'ଣ ଆପଣ ଅତିରିକ୍ତ ଚେଷ୍ଟା କରିକି କ୍ଲିନିସିଆନ୍ ଙ୍କୁ ଅତିଶୀଘ୍ର ଜଣେଇବେ?	Attitude<br><br>ମନୋଭାବ		
	25.	Do you know about the work principles of equipment/instruments used in your laboratory, e.g. Autoclave; Hot Air oven, Sahli's apparatus, Neubauer counting chamber?<br><br>କ'ଣ ଆପଣ ନିଜ ଲାବୋରେଟୋରୀରେ ବ୍ୟବହୃତ ଉପକରଣ/ ସରଞ୍ଜାମ ଗୁଡିକର କାର୍ଯ୍ୟ ପ୍ରଣାଳୀ ବିଷୟରେ ଜାଣନ୍ତି କି, ଉଦାହରଣ ସ୍ୱରୂପ ଅଟୋକ୍ଲେଭ୍, ହଟ୍ ଏଆର୍ ଓଭନ୍, ସାହିଲ୍ ଆପାରଟସ୍, ନିଉବର୍ କାଉଣ୍ଟିଙ୍ଗ ଚ୍ୟାମ୍ବର୍?	Knowledge<br><br>ଜ୍ଞାନ		
	26.	Do you consider that the patient counselling/ preparation before clinical sample collection?	Attitude		

		ଆପଣ ନମୁନା ସଂଗ୍ରହ ପୂର୍ବରୁ ରୋଗୀଙ୍କ କାଉନ୍ସେଲିଂ/ ପ୍ରସ୍ତୁତି ବିଚାର କରନ୍ତି କି?	ମନୋଭାବ		
	27.	Do you know all the equipment/instrument should be labelled with their respective unique I.D.s, date of purchase, date of installation, date of putting into service, date of the last calibration, and name and contact of address mechanic whom to inform in case of emergency?<br><br>କ'ଣ ଆପଣ ଉପକରଣ/ ସରଞ୍ଜାମ ଗୁଡିକୁ ସେମାଙ୍କ ୟୁନିକ୍ ଆଇଡି, କ୍ରୟ ତାରିଖ, ଇନଷ୍ଟଲେସନ୍ ତାରିଖ, କାର୍ଯ୍ୟ ଆରମ୍ଭ କରିବା ତାରିଖ, ସର୍ଭିସିଂ ତାରିଖ, ଶେଷ କ୍ୟାଲିବରେସନ୍ ତାରିଖ ଏବଂ ମେକାନିକ୍ ଙ୍କ ନାମ, ଠିକଣା ଯାହାଙ୍କୁ ଅପାତକାଳୀନ ସମୟରେ ସୂଚିତ କରାଯିବ, ଏସବୁ ତଥ୍ୟ ଦ୍ୱାରା ଚିହ୍ନଟ କରାଯିବା ଆବଶ୍ୟକ ବୋଲି ଜାଣନ୍ତି କି?	Knowledge<br><br>ଜ୍ଞାନ		
	28.	Do you feel that human values and ethics are not much important in delivering Laboratory Services at your facility?<br><br>ଆପଣ ଭାବନ୍ତି କି ଆପଣ ଙ୍କ କେନ୍ଦ୍ରରେ ଲାବୋରେଟୋରୀ ସେବା ପ୍ରଦାନ କରିବାରେ ମାନବିକ ମୂଲ୍ୟ ଏବଂ ଆଚାର ଆବଶ୍ୟକ ଅଟନ୍ତି ନାହିଁ ?	Attitude<br><br>ମନୋଭାବ		
	29.	Do you know what SOPs are?<br><br>ଆପଣ SOP କ'ଣ ବୋଲି ଜାଣିଛନ୍ତି କି?	Knowledge<br><br>ଜ୍ଞାନ		
	30.	Do you feel that it is wastage of time in labelling all the equipment/instrument with their respective unique I.D.s, date of purchase, date of installation, date of putting into service, date of the last calibration, and name and contact of address mechanic whom to inform in case of emergency?<br><br>ଆପଣ ଭାବନ୍ତି କି ଉପକରଣ/ ସରଞ୍ଜାମ ଗୁଡିକୁ ସେମାଙ୍କ ୟୁନିକ୍ ଆଇଡି, କ୍ରୟ ତାରିଖ, ଇନଷ୍ଟଲେସନ୍ ତାରିଖ, କାର୍ଯ୍ୟ ଆରମ୍ଭ କରିବା ତାରିଖ, ସର୍ଭିସିଂ ତାରିଖ, ଶେଷ କ୍ୟାଲିବରେସନ୍ ତାରିଖ ଏବଂ ମେକାନିକ୍ ଙ୍କ ନାମ, ଠିକଣା ଯାହାଙ୍କୁ ଅପାତକାଳୀନ ସମୟରେ ସୂଚିତ କରାଯିବ, ଏସବୁ ତଥ୍ୟ ଦ୍ୱାରା ଚିହ୍ନଟ କରାଯିବା ସମୟର ଅପଚୟ ଅଟେ?	Attitude<br><br>ମନୋଭାବ		
6	31.	Do you consider that the conformity of patient identification before collection of any sample is very much important?	Attitude		

		କ'ଣ ଆପଣ ବିଚାର କରନ୍ତି କି ନମୁନା ସଂଗ୍ରହ ପୂର୍ବରୁ ରୋଗୀଙ୍କ ପରିଚୟ ସୁନିଶ୍ଚିତ କରିବା ନିତାନ୍ତ ଆବଶ୍ୟକ ଅଟେ?	ମନୋଭାବ		
	32.	Do you feel the importance of informing competent authorities in case any equipment goes out of order?<br><br>କୌଣସି ଉପକରଣ ତ୍ରୁଟି ପ୍ରକାଶ ହେବ କ୍ଷେତ୍ରରେ ଆପଣ ସକ୍ଷମ ଅଧିକାରୀଙ୍କୁ ସୂଚନା ଦେବାକୁ ଆବଶ୍ୟକ ଅନୁଭବ କରନ୍ତି କି ?	Attitude<br><br>ମନୋଭାବ		
	33.	Do you know that after collecting the sample, if transportation is required, the sample is carried in closed sample carrier, with the conditions as per test required?<br><br>କ'ଣ ଆପଣ ଜାଣନ୍ତି କି ନମୁନା ସଂଗ୍ରହ ପରେ, ଯଦି ପରିବହନ ଆବଶ୍ୟକ ପଡେ, ପରୀକ୍ଷାର ଆବଶ୍ୟକତା ଅନୁଯାଇ ନମୁନାକୁ ଅବରୁଦ୍ଧ ନମୁନା ବାହକରେ ପରିବହନ କରାଯାଏ?	Knowledge<br><br>ଜ୍ଞାନ		
7	34.	Do you know about the suitability properties of samples for its intended sample test done in his laboratory? (Should explain at least one example like; Renal Function Test and Liver Function Tests require clear serum of the patient blood but if the blood sample is haemolysed it is not suitable for these tests to be performed)<br><br>କ'ଣ ଆପଣ ଲାବୋରଟୋରୀରେ ନମୁନାରେ ହେବାକୁ ଥିବା ପରୀକ୍ଷା ନିମନ୍ତେ ଉପଯୁକ୍ତ ଗୁଣ ବିଷୟରେ ଅବଗତ ଅଟନ୍ତି? (କୌଣସି ଗୋଟିଏ ଉଦାହରଣ ଯେପରି; ରେନାଲ୍ ଫଙ୍କସନ୍ ପରୀକ୍ଷା ଏବଂ ଲିଭର୍ ଫଙ୍କସନ୍ ପରୀକ୍ଷା ପାଇଁ ରୋଗୀର ସ୍ୱଚ୍ଛ ସିରମ୍ ଆବଶ୍ୟକ କିନ୍ତୁ ଯଦି ରକ୍ତ ନମୁନା ହିମୋଲାଇଜ୍ ହୋଇଯାଏ ତେବେ ତାହା ଏହି ପରୀକ୍ଷା ଗୁଡିକ କରିବା ପାଇଁ ଉପଯୁକ୍ତ ରହେ ନାହିଁ)	Knowledge<br><br>ଜ୍ଞାନ		
8	35.	Do you know about all the diagnostic kits and Card based Immunoassay tests done in your lab?<br><br>କ'ଣ ଆପଣ ଆପଣଙ୍କ ଲ୍ୟାବ୍ ରେ ହେଉଥିବା ଡାଇଗ୍ନୋସ୍ଟିକ୍ କୀଟ୍ ଏବଂ କାର୍ଡ ଆଧାରିତ ଇମ୍ୟୁନୋଆସ୍ସେ ପରୀକ୍ଷା ବିଷୟରେ ଜାଣନ୍ତି ଅଟନ୍ତି କି?	Knowledge<br><br>ଜ୍ଞାନ		
	36.	Do you know about the acceptable reference ranges of test results, example; Normal Values of various parameters like; Blood Urea, creatinine, glucose levels etc.<br><br>କ'ଣ ଆପଣ ପରୀକ୍ଷା ଫଳ ଗୁଡିକର ସ୍ୱୀକାର୍ଯ୍ୟ ରେଫରେନ୍ସ ରେଞ୍ଜ୍ ବିଷୟରେ ଜାଣନ୍ତି କି; ଉଦାହରଣ; ବିଭିନ୍ନ ମାନଦଣ୍ଡ ମାନଙ୍କ	Knowledge<br><br>ଜ୍ଞାନ		

		ସାମାନ୍ୟ ମୂଲ୍ୟ ଯେପରି; ରକ୍ତ ୟୁରିଆ, କ୍ରିଏଟିନିନ୍, ରକ୍ତ ଶର୍କରା ସ୍ତର ଇତ୍ୟାଦି|			
	37.	Do you know about the critical values of test results and alarming critical alerts?<br><br>କ'ଣ ଆପଣ ପରୀକ୍ଷା ଫଳ ଗୁଡିକର କ୍ରିଟିକାଲ୍ ମୂଲ୍ୟ ଏବଂ ବିପଦଜନକ ସଙ୍କେତ ବିଷୟରେ ଜାଣନ୍ତି କି ?	Knowledge<br><br>ଜ୍ଞାନ		
	38.	Do you feel the importance of displaying shelf life of inhouse prepared reagents, like Giemsa Stain for blood cell counting and morphology ; Zeil-Neelson stain for Acid fast Bacilli; Buffers for many other reagents, e.g. for biochemistry test?<br><br>କ'ଣ ଆପଣ ଅନୁଭବ କରନ୍ତି ଯେ ନିଜ ଦ୍ୱାରା ପ୍ରସ୍ତୁତ ରିଏଜେଣ୍ଟ୍, ଯେପରିକି ବ୍ଲଡ୍ ସେଲ୍ କାଉଣ୍ଟିଙ୍ଗ୍ ଏବଂ ମର୍ଫୋଲୋଜି ପାଇଁ ଉଦ୍ଦିଷ୍ଟ ଜିମ୍ସା ଷ୍ଟେନ୍ : ଏସିଡ୍ ଫାଷ୍ଟ ବାସିଲାଇ ପାଇଁ ଉଦ୍ଦିଷ୍ଟ ଜିଲ୍- ନିଲ୍ସନ୍ ଷ୍ଟେନ୍ ; ଅନ୍ୟ ରିଏଜେଣ୍ଟ ମାନଙ୍କ ପାଇଁ ବଫର୍ ମାନଙ୍କ ଶେଲ୍ଫ ଲାଇଫ୍ ଡିସ୍-ପ୍ଲେ କରିବା ଉଚିତ , ଉଦାହରଣ ସ୍ୱରୂପ: ବାୟୋକେମେଷ୍ଟ୍ରି ପରୀକ୍ଷା ନିମନ୍ତେ ?	Attitude<br><br>ମନୋଭାବ		
	39.	Do you know about point of care testing?<br><br>କ'ଣ ଆପଣ ପଏଣ୍ଟ ଅଫ୍ କେୟାର୍ ପରୀକ୍ଷା ବିଷୟରେ ଜାଣିଛନ୍ତି କି?	Knowledge<br><br>ଜ୍ଞାନ		
	40.	Do you think that higher education will improve your professional work quality?<br><br>କ'ଣ ଆପଣ ଭାବନ୍ତି କି ଉଚ୍ଚତର ଶିକ୍ଷା ଆପଣଙ୍କ ବୃତ୍ତିଗତ କାର୍ଯ୍ୟର ମାନ ବୃଦ୍ଧି କରିବାରେ ସହାୟକ ହେବ?	Attitude<br><br>ମନୋଭାବ		
	41.	Do you know that the clinical Specimens are required to be retained for some specific period even after successful completion of tests?<br><br>କ'ଣ ଆପଣ ଜାଣନ୍ତି ପରୀକ୍ଷା ଗୁଡିକର ସଫଳ ପୂରଣ ପରେ ମଧ୍ୟ କ୍ଲିନିକାଲ୍ ନମୁନା ଗୁଡିକ ରିଟେନ୍ କରିବା ଆବଶ୍ୟକ ଅଟେ?	Knowledge<br><br>ଜ୍ଞାନ		
	42.	Do you know how the clinical samples are stored during the retention period?<br><br>କ'ଣ ଆପଣ ଜାଣନ୍ତି କି କ୍ଲିନିକାଲ୍ ନମୁନା ଗୁଡିକୁ ରିଟେନ୍ସନ୍ ପିରିୟଡ୍ ରେ କିପରି ଭାବରେ ସଂଗ୍ରହ କରାଯାଏ?	Knowledge<br><br>ଜ୍ଞାନ		
	43.	Do you know how to dispose of various clinical samples after use /after completion of retention period as per prevailing/applicable biomedical waste management rule?	Knowledge		

		କ'ଣ ଆପଣ ଜାଣନ୍ତି କି ବିଭିନ୍ନ କ୍ଲିନିକାଲ୍ ନମୁନା ବ୍ୟବହାର/ ରିଟେନ୍ସନ୍ ପିରିୟଡ଼ର ସମାପ୍ତି ପରେ ବାୟୋମେଡ଼ିକାଲ୍ ୱେଷ୍ଟ ଗୁଡିକୁ ପ୍ରଚଳିତ/ ଉପଯୁକ୍ତ ନିୟମ ଅନୁସାରେ ଡିସପୋଜ୍ କିପରି କରାଯାଏ?	ଜ୍ଞାନ		
	44.	Do you understand the concept of "segregation at source" in relation to the Bio Medical Waste Management?<br><br>କ'ଣ ଆପଣ ବାୟୋମେଡ଼ିକାଲ୍ ୱେଷ୍ଟ ପ୍ରବନ୍ଧନ ଦୃଷ୍ଟିରୁ "ସେଗ୍ରୀଗେସନ୍ ଆଟ୍ ସୋର୍ସ୍ " କନ୍ସେପ୍ଟ ବିଷୟରେ ଅବଗତ ଅଟନ୍ତି ?	Knowledge<br><br>ଜ୍ଞାନ		
9	45.	Do you know about the concept of transcript check ?<br>(Example-One should check and observe whether the name of patient, patient ID, OPD/Wards, rough data of results, the data entered into the lab register or on LIS and the report data entered into the final report format to be given to the patient are same) ?<br><br>କ'ଣ ଆପଣ ଟ୍ରାନ୍ସକ୍ରିପ୍ଟ ଚେକ୍ ର କନ୍ସେପ୍ଟ ବିଷୟରେ ଜାଣନ୍ତି ? (ଉଦାହରଣ - ଜଣେ ଯାଞ୍ଚ କରିବା ଉଚିତ କି ଲ୍ୟାବ୍ ରେଜିଷ୍ଟର୍ କିମ୍ବା ଏଲ୍.ଆଇ.ଏସ୍ ଏବଂ ରୋଗୀଙ୍କୁ ଦିଆ ଯିବାକୁ ଥିବା ଅନ୍ତିମ ରିପୋର୍ଟ ରେ ଭରା ଯାଇଥିବା ତଥ୍ୟ ସମାନ ଅଟେ, ଯେପରି ରୋଗୀଙ୍କ ନାମ, ରୋଗୀ ଆଇ.ଡି, ଓ.ପି.ଡି / ୱାର୍ଡ୍, ଫଳାଫଳର ରଫ୍ ଡାଟା)	Knowledge<br><br>ଜ୍ଞାନ		
	46.	Do you know the standard units used in test reports of various parameters, like the units used in blood sugar, Hb, TLC, DLC, and platelets count etc.<br><br>ଆପଣ ମାନକ ମାନଙ୍କ ଏକକ ଯାହା ପରୀକ୍ଷା ଫଳପତ୍ରରେ ବିଭିନ୍ନ ମାପଦଣ୍ଡ ଯେପରି ରକ୍ତ ଶର୍କରା, ଏଚବି, ଟିଏଲସି, ଡିଏଲସି ଏବଂ ପ୍ଲେଟଲେଟ୍ ଗଣନା ଇତ୍ୟାଦି ରେ ବ୍ୟବହାର ହୁଏ ଜାଣନ୍ତି କି ?	Knowledge<br><br>ଜ୍ଞାନ		
10	47.	Do you know about general (Chemical, Biological and non-biological like electrical safety, fire safety) and specific safety precautions in clinical laboratory like, how to deal with blood spill, exposure of your skin to acid or alkalis etc?<br><br>କ'ଣ ଆପଣ କ୍ଲିନିକାଲ୍ ଲାବୋରେଟୋରୀରେ ବିଶିଷ୍ଟ ସୁରକ୍ଷା ସତର୍କତା ଯେପରି ସାଧାରଣ (ରାସାୟନିକ, ଜୈବିକ ଏବଂ ଅଣ ଜୈବିକ ଯେମିତି ବୈଦୁତିକ, ଅଗ୍ନି ନିରାପତ୍ତା) ବିଷୟରେ ଜାଣିଛନ୍ତି	Knowledge<br><br>ଜ୍ଞାନ		

		ଯେମିତି ରକ୍ତ ଢଳିଯିବା, ଏସିଡ୍/ ଆଲକାଲି ର ଚର୍ମ ସମ୍ପର୍କରେ ଆସିବା ଇତ୍ୟାଦିକୁ କେମିତି ପ୍ରତିକ୍ରିୟା କରିବେ?			
	48.	Do you know what are the precautions required to deal with fire safety?<br><br>କ'ଣ ଆପଣ ଅଗ୍ନି ନିରାପତ୍ତା ସମ୍ବନ୍ଧୀୟ ସତର୍କତା ବିଷୟରେ ଅବଗତ ?	Knowledge<br><br>ଜ୍ଞାନ		
	49.	Have you ever been trained/ participated in any fire safety training/ mock drill?<br><br>କ'ଣ ଆପଣ କେବେ କୌଣସି ଅଗ୍ନି ନିରାପତ୍ତା ପ୍ରଶିକ୍ଷଣ / ମକ୍ ଡ୍ରିଲ୍ ରେ ପ୍ରଶିକ୍ଷଣ/ ଭାଗ ନେଇଛନ୍ତି ?	Knowledge<br><br>ଜ୍ଞାନ		
	50.	Do you know which precautions are required to deal with electrical safety?<br><br>କ'ଣ ଆପଣ ଜାଣିଛନ୍ତି କି ବୈଦୁତିକ ନିରାପତ୍ତାରେ କେଉଁ ସତର୍କତା ଅବଲମ୍ବନ କରାଯାଏ ?	Knowledge<br><br>ଜ୍ଞାନ		
	51.	Do you know that in the electrical fittings, proper earthing is important to avoid electric shocks from any metallic equipment and its damage?<br><br>କ'ଣ ଆପଣ ଜାଣିଛନ୍ତି କି ଧାତବ ଉପକରଣ ଏବଂ ଏହାର କ୍ଷୟ ଯୋଗୁଁ ହେଉଥିବା ବୈଦ୍ୟୁତିକ ଆଘାତରୁ ସୁରକ୍ଷା ପାଇଁ ବୈଦ୍ୟୁତିକ ଫିଟିଙ୍ଗ୍ ଗୁଡିକର ସଠିକ୍ ଆର୍ଥିଙ୍ଗ୍ କରାଇବା ଆବଶ୍ୟକ ?	Knowledge<br><br>ଜ୍ଞାନ		
	52.	Do you feel that FIFO is not much useful in maintaining inventory for reagents?<br><br>ଆପଣ ଅନୁଭବ କରନ୍ତି କି ଫାର୍ଷ୍ଟ ଇନ୍ ଏବଂ ଫାର୍ଷ୍ଟ ଆଉଟ୍ (FIFO) ରିଏଜେଣ୍ଟ୍ ମାନଙ୍କ ଇନ୍-ଭେଣ୍ଟୋରି ମେଣ୍ଟେନ୍ ରେ ଉପଯୋଗୀ ଅଟେ ନାହିଁ?	Attitude<br><br>ମନୋଭାବ		
	53.	Do you know about the laboratory hygiene and infection control practices / Policy in the laboratory?<br><br>କ'ଣ ଆପଣ ଲାବୋରେଟୋରୀରେ ଲାବୋରେଟୋରୀ ସ୍ୱଚ୍ଛତା ଏବଂ ସଂକ୍ରମଣ ନିୟନ୍ତ୍ରଣ ଅଭ୍ୟାସ/ ନିୟମ ବିଷୟରେ ଜାଣନ୍ତି କି ?	Knowledge<br><br>ଜ୍ଞାନ		
	54.	Do you think calibration and validation of equipment and the test procedures are important for proper test results?<br><br>ଆପଣ ଭାବନ୍ତି କି କ୍ୟାଲିବରେସନ୍ ଏବଂ ଉପକରଣର ଭ୍ୟାଲିଡେସନ୍ ଏବଂ ପରୀକ୍ଷା ପ୍ରକ୍ରିୟା ସଠିକ ପରକ୍ଷା ଫଳ ପାଇଁ ଆବଶ୍ୟକ?	Attitude<br><br>ମନୋଭାବ		

	55.	Do you know what the biosafety equipment are?<br><br>ଆପଣ ଜୈବସୁରକ୍ଷା ଉପକରଣ କ'ଣ ବୋଲି ଜାଣିଛନ୍ତି କି ?	Knowledge<br><br>ଜ୍ଞାନ		
	56.	Do you know that the micropipettes or auto pipettes should be used in place of mouth pipetting?<br><br>କ'ଣ ଆପଣ ଜାଣିଛନ୍ତି ଯେ ମାଉଥ୍ ପାଇପେଟିଙ୍ଗ୍ ସ୍ଥାନ ରେ ମାଇକ୍ରୋପାଇପେଟ୍ ଅବା ଅଟୋ ପାଇପେଟ୍ ର ବ୍ୟବହାର ହେବା ଉଚିତ ?	Knowledge<br><br>ଜ୍ଞାନ		
	57.	Do you know that the labelling dating, handle, store and dispose of chemicals, dyes, reagents, and solutions should be as per applicable legislations is mandatory?<br><br>କ'ଣ ଆପଣ ଜାଣିଛନ୍ତି କି କେମିକାଲ୍, ଡାଇ, ରୀଏଜେଣ୍ଟ୍ ଏବଂ ସଲ୍ୟୁସନ୍ ଗୁଡିକର ଲେବଲିଙ୍ଗ୍ ଡେଟିଂ, ହ୍ୟାଣ୍ଡେଲ୍, ଭଣ୍ଡାରଣ ଏବଂ ଡିସପୋଜ୍ ଉପଯୁକ୍ତ ନିୟମ ଅନୁସାରେ ହେବା ଆବଶ୍ୟକ ଅଟେ?	Knowledge<br><br>ଜ୍ଞାନ		
	58.	Do you know that every chemical accompanied by a document called as Master safety data sheet (MSDS), which explains "how that chemical can be used safely" explaining specifically the safety precautions specifically to all the requirements?<br><br>କ'ଣ ଆପଣ ଜାଣିଛନ୍ତି କି ପ୍ରତ୍ୟୋକ କେମିକାଲ୍ ସହ ଏକ ମାଷ୍ଟର୍ ସେଫ୍ଟୀ ଡାଟା ଶୀଟ୍ (MSDS) ନାମକ ଡକୁମେଣ୍ଟ୍ ଥାଏ ଯାହା ବର୍ଣନା କରେ କି " ସେହି କେମିକାଲ୍ କିପରି ସୁରକ୍ଷିତ ଭାବରେ ବ୍ୟବହାର କରାହେବ", ଯାହାକି ସମସ୍ତ ସଠିକ୍ ସୁରକ୍ଷା ସତର୍କତା ଆବଶ୍ୟକତାକୁ ବର୍ଣ୍ଣନା କରେ?	Knowledge<br><br>ଜ୍ଞାନ		
	59.	Are you aware of that the burning type of syringe needle destroyers are not recommended these days? if not then why?<br><br>କ'ଣ ଆପଣ ଜାଣିଛନ୍ତି ବର୍ତ୍ତମାନ ସମୟରେ ବର୍ଣ୍ଣିଙ୍ଗ୍ ଟାଇପ୍ ସିରିଞ୍ଜ ନିଡିଲ୍ ନଷ୍ଟ ଉପକରଣର ବ୍ୟବହାର ପାଇଁ ପରାମର୍ଶ ଦିଆ ଯାଉ ନାହିଁ? ଯଦି ନାହିଁ, ତେବେ କେଉଁ କାରଣ ଯୋଗୁଁ?	Knowledge<br><br>ଜ୍ଞାନ		
	60.	Do you know that how does the mechanical syringe needle destroyers are safe to be used?<br><br>କ'ଣ ଆପଣ ଜାଣନ୍ତି ମେକାନିକାଲ୍ ସିରିଞ୍ଜ ନିଡିଲ୍ ନଷ୍ଟ ଉପକରଣ ବ୍ୟବହାର ପାଇଁ କେତେ ସୁରକ୍ଷିତ ଅଟେ?	Knowledge<br><br>ଜ୍ଞାନ		
	61.	Do you know the different type of disinfectants used in the laboratory for disinfection of floors,	Knowledge		

		work benches, environmental disinfections and discarding jars?<br><br>କ'ଣ ଆପଣ ଲାବୋରେଟୋରୀ ଭିତରେ ଚଟାଣ, ବେଞ୍ଚ, ପରିବେଶ ସଙ୍କ୍ରମଣ ଏବଂ ଜାର୍ ଡିସ୍କାର୍ଡିଂର ସଫେଇ ପାଇଁ ବ୍ୟବହାର ହେଉଥିବା ବିଭିନ୍ନ ଡିସ୍‌ଇନ୍‌ଫେକ୍ଟାଣ୍ଟ ବିଷୟରେ ଜାଣିଛନ୍ତି କି?	ଜ୍ଞାନ		
	62.	Do you know about Needle Stick Injuries?<br><br>କ'ଣ ଆପଣ ନିଡିଲ୍ ଷ୍ଟିକ୍ ଆଘାତ ବିଷୟରେ ଜାଣିଛନ୍ତି କି?	Knowledge<br><br>ଜ୍ଞାନ		
	63.	Do you feel that you have acquired all knowledge and skills for delivering your assigned work?<br><br>ଆପଣ ଭାବନ୍ତି କି ଆପଣଙ୍କୁ ନ୍ୟସ୍ତ ହୋଇଥିବା କାର୍ଯ୍ୟକୁ ପୁରଣ କରିବା ପାଇଁ ସମସ୍ତ ଜ୍ଞାନ ଏବଂ କୌଶଳ କୁ ଆପଣ ଆହରଣ କରିସାରିଛନ୍ତି ?	Attitude<br><br>ମନୋଭାବ		
	64.	Do you know about the needle stick injury recording process?<br><br>କ'ଣ ଆପଣ ନିଡିଲ୍ ଷ୍ଟିକ୍ ଆଘାତର ରେକର୍ଡିଂ ପ୍ରକ୍ରିୟା ବିଷୟରେ ଜାଣିଛନ୍ତି କି ?	Knowledge<br><br>ଜ୍ଞାନ		
	65.	In the case of breakage of glass tubes containing clinical samples or some microbial suspension, inside the centrifuge machine, do you know how to handle this situation?<br><br>କ'ଣ ଆପଣ ଜାଣିଛନ୍ତି କି ସେଣ୍ଟ୍ରିଫ୍ୟୁଜ୍ ଉପକରଣ ମଧ୍ୟରେ ଯଦି କୌଣସି ନମୁନା ଥିବା କାଚ ଟ୍ୟୁବ୍ କିମ୍ବା କିଛି ମାଇକ୍ରୋବିଆଲ୍ ସସ୍ପେନ୍‌ସନ୍ ଘଟେ ତେବେ ଏହାକୁ କିପରି ସମ୍ଭାଳିବାକୁ ହୁଏ?	Knowledge<br><br>ଜ୍ଞାନ		
	66.	Do you think, the application of quality control concept can improve the quality of laboratory test reports?<br><br>ଆପଣ ଭାବନ୍ତି କି ଗୁଣବତ୍ତା ନିୟନ୍ତ୍ରଣ ର କନସେପ୍ଟ ର ଉପଯୋଗ ଲାବୋରେଟୋରୀ ଟେସ୍ଟ ରିପୋର୍ଟର ଗୁଣବତ୍ତା ଉନ୍ନତି କରିପାରେ?	Attitude<br><br>ମନୋଭାବ		
	67.	Do you know about the type of infection, that could be acquired during sample collection?<br><br>କ'ଣ ଆପଣ ଜାଣିଛନ୍ତି ନମୁନା ସଂଗ୍ରହ କଲା ବେଳେ କେଉଁ ପ୍ରକାରର ସଙ୍କ୍ରମଣ ହୋଇପାରେ?	Knowledge<br><br>ଜ୍ଞାନ		
	68.	Do you know about the precautions measure while working in the laboratory?	Knowledge<br><br>ଜ୍ଞାନ		

		କ'ଣ ଆପଣ ଜାଣିଛନ୍ତି ଲାବୋରେଟୋରୀରେ କାର୍ଯ୍ୟ କଲା ବେଳେ କେଉଁ ପ୍ରକାରର ସତର୍କତା ଅବଲମ୍ବନ କରାଯାଏ ?			
	69.	Do you know that laboratory environment can be contaminated while working with clinical samples/ live organisms?<br><br>କ'ଣ ଆପଣ ଜାଣିଛନ୍ତି କି କ୍ଲିନିକାଲ୍ ନମୁନା/ ଜୀବନ୍ତ ଅଣୁଜୀବ ସହ କାମ କଲା ବେଳେ ଲାବୋରେଟୋରୀ ପରିବେଶ ପ୍ରଦୂଷିତ ହୋଇପାରେ?	Knowledge<br><br>ଜ୍ଞାନ		
	70.	Do you know the protocols could be followed in case of exposure to body fluids (as applicable)<br><br>କ'ଣ ଆପଣ ଶରୀର ନିର୍ଗତ ତରଳର ସମ୍ପର୍କରେ (ଯାହା ଉପଯୁକ୍ତ) ଆସିବା କ୍ଷେତ୍ରରେ ଥିବା ପ୍ରକ୍ରିୟା ବିଷୟରେ ଜାଣିଛନ୍ତି ?	Knowledge<br><br>ଜ୍ଞାନ		
	71.	Do you know that hands are the most important vehicle for microbial transmission?<br><br>କ'ଣ ଆପଣ ଜାଣିଛନ୍ତି କି ଅଣୁଜୀବ ସଂକ୍ରମଣ ଲାଗି ହାତ ସବୁଠୁ ପ୍ରଧାନ ବାହକ?	Knowledge<br><br>ଜ୍ଞାନ		
	72.	Do you think, it is important for Medical Laboratory Technicians to respect the diversity, dignity, values and beliefs of patients /clients and colleagues, for delivering Laboratory Services?<br><br>ଆପଣ ଭାବନ୍ତିକି ଲାବୋରେଟୋରୀ ଟେକ୍ନୋଲୋଜିଷ୍ଟଙ୍କ ପାଇଁ ରୋଗୀ/ ଗ୍ରାହକ ଏବଂ ସହକର୍ମୀଙ୍କ ବିବିଧତା, ସ୍ୱାଭିମାନ, ମୂଲ୍ୟ ଏବଂ ବିଶ୍ୱାସକୁ ସମ୍ମାନ ଦେବା ଲାବୋରେଟୋରୀ ସେବା ପ୍ରଦାନ କରିବାରେ ଜରୁରୀ ଅଟେ?	Attitude<br><br>ମନୋଭାବ		
	73.	Do you know that the proper handwashing with soap and water reduces hospital acquired infections by more than 80 %?<br><br>କ'ଣ ଆପଣ ଜାଣନ୍ତି ଚିକିତ୍ସାଳୟରୁ ହୋଇଥିବା ସଂକ୍ରମଣ ଠିକ୍ ଭାବେ ହାତ ଧୋଇବା ଦ୍ୱାରା ୮୦ % ପର୍ଯ୍ୟନ୍ତ କମ୍ ହୋଇପାରେ?	Knowledge<br><br>ଜ୍ଞାନ		
	74.	Do you know under what circumstances alcohol-based hand sanitizer are recommended for use?<br><br>କ'ଣ ଆପଣ ଜାଣନ୍ତି କି କେଉଁ ପରିସ୍ଥିତିରେ ଆଲ୍କୋହଲ୍ ଆଧାରିତ ହ୍ୟାଣ୍ଡ୍ ସାନିଟାଇଜର୍ ବ୍ୟବହାର ପାଇଁ ପରାମର୍ଶ ଦିଆଯାଏ ?	Knowledge<br><br>ଜ୍ଞାନ		
	75.	Do you know that if you encounter any sharp cuts, then how will you respond?	Knowledge<br><br>ଜ୍ଞାନ		

		ଯଦି ଆପଣ କୌଣସି କଟିଯିବା ଯୋଗୁଁ କ୍ଷତ ଦେଖନ୍ତି, ତେବେ ଆପଣ କିପରି ପ୍ରତିକ୍ରିୟା କରିବେ?			
	76.	Do you feel preparation and display of SOPs for all the processes and equipment are waste of resources ?<br><br>ଆପଣ ଅନୁଭବ କରନ୍ତି କି ସମସ୍ତ ପ୍ରକ୍ରିୟା ଏବଂ ଉପକରଣ ଗୁଡିକ ପାଇଁ ଏସ୍.ଓ.ପି ର ପ୍ରସ୍ତୁତି ଏବଂ ଡିସ୍-ପ୍ଲେ କରିବା ସମ୍ବଳର ଅପଚୟ ଅଟେ?	Attitude<br><br>ମନୋଭାବ		

C.2. Observational Tools (Skills)
ଗ୨. ପ୍ରତ୍ୟକ୍ଷ ଅନୁଧ୍ୟାନ ଟୁଲ (କୌଶଳ)

Score Definition

ମୂଲ୍ୟାଙ୍କନର ପରିଭାଷା

A. None—No demonstrated skills at all/does not perform the task(s) completely
କ. ନାହିଁ - କୌଣସି କୌଶଳ ଉପସ୍ଥିତ ନାହିଁ/ କାର୍ଯ୍ୟ ସମ୍ପୂର୍ଣ୍ଣ କରି ନାହାନ୍ତି|
B. Limited Demonstrated very limited strengths/skills in this area
ଖ. ଏହି କ୍ଷେତ୍ରରେ ବହୁତ କମ ମାତ୍ରାରେ ସାମର୍ଥ୍ୟ / କୌଶଳର ପ୍ରଦର୍ଶନ|
C. Some—Demonstrated some ability/skills in this area.
ଗ. ସ୍ୱଳ୍ପ- ଏହି କ୍ଷେତ୍ରରେ ସ୍ୱଳ୍ପ ସାମର୍ଥ୍ୟ/ କୌଶଳର ପ୍ରଦର୍ଶନ|
D. Strong—Demonstrated strong skills/strength in this area.
ଘ. ଦୃଢ - ଏହି କ୍ଷେତ୍ରରେ ଦୃଢ ସାମର୍ଥ୍ୟ/ କୌଶଳର ପ୍ରଦର୍ଶନ|
E. Excellent—Demonstrated excellent skills/strength in this area.
ଙ. ଅସାଧାରଣ - ଏହି କ୍ଷେତ୍ରରେ ଅସାଧାରଣ ସାମର୍ଥ୍ୟ/ କୌଶଳର ପ୍ରଦର୍ଶନ|
F. Not applicable.
ଚ. ଆବଶ୍ୟକତା ନାହିଁ|
G. Don’t know- Not even heard about that skill.
ଛ. ଜାଣି ନାହାନ୍ତି - କୌଶଳ ବିଷୟରେ ଅନଭିଜ୍ଞ|
H. Skill limitation is clearly related to resource limitations.
ଜ. ସୀମିତ ସମ୍ବଳ କାରଣରୁ ସୀମିତ କୌଶଳର ପ୍ରଦର୍ଶନ ସ୍ପଷ୍ଟ ପରିଲକ୍ଷିତ|

Domain Code ଡୋମେନ୍ କୋଡ୍	S. N. କ୍ରମିକ ସଂଖ୍ୟା	Observation Points ଅନୁଧ୍ୟାନ ବିନ୍ଦୁ	Means of Verification ସତ୍ୟାପନର ସାଧନ	Response (Score) ପ୍ରତିକ୍ରିୟା (ଅଙ୍କ)
1	1.	Responsibilities towards Patients and their attendants<br><br>ରୋଗୀ ଏବଂ ତାଙ୍କ ପରିଚାରକଙ୍କ ପ୍ରତି ଦାୟିତ୍ୱ	Listens to patient’s ideas and concerns. Doesn’t rush patient and doesn’t take too much time; Maintains speaking in low pitch voice; Provides documentation that is accurate and complete<br><br>ରୋଗୀଙ୍କ ବିଚାର ଏବଂ ଚିନ୍ତା ଗୁଡିକୁ ଶୁଣିବା ରୋଗୀଙ୍କୁ ବ୍ୟତିବ୍ୟସ୍ତ କରନ୍ତିନି ଏବଂ ଅତ୍ୟଧିକ ସମୟ ନିଅନ୍ତିନି; ଧୀର ସ୍ୱରରେ କଥା	

			ବାର୍ତ୍ତା ବଜାୟ ରଖନ୍ତି; ତଥ୍ୟ ପ୍ରଲେଖନ ଦିଅନ୍ତି ଯାହା ସଠିକ ଏବଂ ସମ୍ପୂର୍ଣ ଅଟେ|	
	2.	Compliances towards legislation that applies for Medical Laboratory Services in that facility<br><br>ଏହି କେନ୍ଦ୍ରରେ ମେଡିକାଲ ଲାବୋରେଟୋରୀ ପାଇଁ ଥିବା ନିୟମର ଅନୁପାଳନ	Documentation in relation to ; Notifiable Diseases, Biomedical Waste Management Rules 2016 , Minimum Standards of Medical Diagnostic Laboratories as per notifications from Government bodies from time to time like; Clinical Establishment Act<br><br>ନୋଟିଫାଏବଲ୍ ଡିଜିଜ୍, ବାୟୋ ମେଡିକାଲ ୱେଷ୍ଟ ସଂକ୍ରାନ୍ତିୟ ମ୍ୟାନେଜ୍-ମେଣ୍ଟ୍ ରୁଲ୍ ୨୦୧୬, ସମୟ ସମୟରେ ସରକାରୀ ସଂସ୍ଥା ପକ୍ଷରୁ ଆସୁଥିବା ମେଡିକାଲ ଡାଇଗ୍ନୋଷ୍ଟିକ ଲାବୋରେଟୋରୀଙ୍କ ପାଇଁ ନ୍ୟୁନତମ ମାନକ ସମ୍ପର୍କିତ ସୂଚନା ଯେପରି; କ୍ଲିନିକାଲ୍ ଏସ୍ଟାବ୍ଲୀଶ୍-ମେଣ୍ଟ୍ ଆକ୍ଟ ସଂକ୍ରାନ୍ତିୟ ଅଭିଲେଖନ	
2	3.	Corrective Action and Preventive Action (CA-PA)?<br><br>କରେକ୍ଟିଭ୍ ଆକ୍ସନ୍ ଏବଂ ପ୍ରିଭେଣ୍ଟିଭ୍ ଆକ୍ସନ୍ (ସିଏପିଏ)?	Documentation in terms of<br>-Incident/Accident Log/Record<br>-CA-PA Log/ Record<br><br>-ଘଟଣା/ ଦୁର୍ଘଟଣା ଲଗ୍/ ରେକର୍ଡ୍<br>-ସି.ଏ.ପି.ଏ ଲଗ୍/ ରେକର୍ଡ୍<br>ସଂକ୍ରାନ୍ତିୟ ଅଭିଲେଖନ	
	4.	Simple calculations<br><br>ସାଧାରଣ ଗଣନା	Final Result of Test like ;<br><br>**1.** Indirect bilirubin (conjugated bilirubin) is calculated as follows:<br><br>Indirect bilirubin (Unconjugated) = Total bilirubin – direct (Conjugated) bilirubin.<br><br>**2.** Calculation of Prothrombin time Index (PTI)<br>PTI = PT of patient x 100 / PT of normal control<br><br>**3.** Calculation of WBCs observed in an improved Neubauer Counting Chamber<br><br>ପରୀକ୍ଷା ଗୁଡିକର ଅନ୍ତିମ ପରିଣାମ ଯେପରି;<br><br>୧ . ଇନ୍- ଡାଇରେକ୍ଟ ବିଲିରୁବିନ୍ (କଞ୍ଜୁଗେଟେଡ୍ ବିଲିରୁବିନ୍) କୁ ନିମ୍ନଲିଖିତ ରୂପେ ଗଣନା କରାଯାଏ :<br><br>ଇନ୍- ଡାଇରେକ୍ଟ ବିଲିରୁବିନ୍ (କଞ୍ଜୁଗେଟେଡ୍ ବିଲିରୁବିନ୍)= ଟୋଟାଲ୍ ବିଲିରୁବିନ୍- ଡାଇରେକ୍ଟ (କଞ୍ଜୁଗେଟେଡ୍) ବିଲିରୁବିନ୍<br>୨ . ପ୍ରୋଥ୍ରମ୍ବିନ୍ ଟାଇମ୍ ଇଣ୍ଡେକ୍ସ (ପି.ଟି.ଆଇ)ର ଗଣନା	

			ପି.ଟି.ଆଇ = ରୋଗୀଙ୍କ ପି.ଟି * ୧୦୦ / ସାଧାରଣ ନିୟନ୍ତ୍ରକର ପି.ଟି<br>୩ . ଉନ୍ନତ ନିଉବର୍ କାଉଣ୍ଟିଙ୍ଗ୍ ଚ୍ୟାମ୍ବର୍ ରେ ଅନୁଧ୍ୟାନ ହୋଇଥିବା ଶ୍ୱେତ ରକ୍ତ କୋଷ (WBC) ର ଗଣନା	
	5.	Calibration<br><br>କ୍ୟାଲିବରେସନ୍	Internal Calibration of Commonly used equipment e.g., Weighing Balance; and Micro Pipettes etc and **check their calibration records**<br><br>ସାଧାରଣତଃ ବ୍ୟବହାର ହେଉଥିବା ଉପକରଣ, ଉଦାହରଣ ସ୍ୱରୂପ ୱେଇଂ ବାଲାନ୍ସ ଏବଂ ମାଇକ୍ରୋ ପାଇପେଟ୍ ଇତ୍ୟାଦି, ଇଣ୍ଟରନାଲ୍ କ୍ୟାଲିବରେସନ୍ ଏବଂ **ତାହାର କ୍ୟାଲିବରେସନ୍ ରେକର୍ଡ୍ ଯାଞ୍ଚ୍ କରିବା**	
	6.	Efficacy Testing<br><br>ଏଫିକେସି ଟେଷ୍ଟିଙ୍ଗ୍	The efficacy of any equipment may vary time to time because of many technical or mechanical reasons, hence it needs to be checked frequently that the concerned equipment is working as per required efficacy for example -Hot Air Oven; Autoclave or any other sterilizer; Incubators; pH meters, any other instruments/ equipment used in the laboratory<br>If done, **check the records.**<br><br>କୌଣସି ଉପକରଣର ପ୍ରଭାବ ସମୟ ସମୟରେ ଅନେକ ବୈଷୟିକ କିମ୍ବା ଯାନ୍ତ୍ରିକ କାରଣରୁ ପରିବର୍ତ୍ତନ ହୋଇଥାଏ , ତେଣୁ ଏହାକୁ ପ୍ରାୟ ସମୟରେ ଯାଞ୍ଚ୍ କରିବାକୁ ହୁଏ ଯେମିତି କି ସମ୍ବନ୍ଧିତ ଉପକରଣ ଆବଶ୍ୟକୀୟ ପ୍ରଭାବ ସହିତ କାମ କରୁଛି।<br><br>ଉଦାହରଣ ସ୍ୱରୂପ - ହଟ୍ ଏୟାର୍ ଓଭନ୍, ଅଟୋ କ୍ଲେଭ୍ କିମ୍ବା ଅନ୍ୟ ବିସଙ୍କ୍ରମଣ ଯନ୍ତ୍ର, ଇନ୍କ୍ୟୁବେଟର୍, ପିଏଚ୍ ମିଟର୍ ଇତ୍ୟାଦି<br><br>ଯଦି କରାଯାଇଥାଏ, **ତେବେ ଏହାର ରେକର୍ଡ୍ ଯାଞ୍ଚ୍**	

	7.	First in First Out (FI-FO or First Entry and first out (FE-FO)<br>With special reference to Shelf life/ Expiry Date<br><br>ଫାର୍ଷ୍ଟ ଇନ୍ ଫାର୍ଷ୍ଟ ଆଉଟ୍ (FI-FO) କିମ୍ବା ଫାର୍ଷ୍ଟ ଏଣ୍ଟ୍ରି ଫାର୍ଷ୍ଟ ଆଉଟ୍ (FE-FO) ଶେଲ୍ଫ୍ ଲାଇଫ / ଏକ୍ସପାଇରି ଡେଟ୍ ସନ୍ଦର୍ଭ ରେ	Whether First in First out concept is followed?<br><br>Verify the following?<br><br>1. Check the Inventory for any expired materials kept in the stock including perishable items in refrigerator<br>2. Match the stock the with the stock register or digital data., if any , as applied therein<br>3. Check that the recently procured items are kept serially behind the previously procured items<br>4. Whether the stocks are placed as per their hazardous category requirements for example A. Alcohols are stored separately under lock B. Acids are always kept separately on floor only.<br><br>ଫାର୍ଷ୍ଟ ଇନ୍ ଫାର୍ଷ୍ଟ ଆଉଟ୍ କନସେପ୍ଟ୍ ଅନୁସରଣ କରା ଯାଉଛି କି ?<br>ନିମ୍ନଲିଖିତ ମାନଙ୍କ ସତ୍ୟାପନ?<br><br>୧. ରେଫ୍ରୀଜରେଟର୍ ରେ ଏକ୍ସପାୟାର୍ଡ ଜିନିଷ ସମେତ ସ୍ୱଳ୍ପ ଆୟୁ ଯୁକ୍ତ ଜିନିଷର ଉପସ୍ଥିତ ଯାଞ୍ଚ କରିବା<br><br>୨. ଜିନିଷ ଗୁଡିକର ଷ୍ଟକ୍ ରେଜିଷ୍ଟର୍/ କିମ୍ବା ଡିଜିଟାଲ୍ ଡାଟା ଯଦି କିଛି ଥାଏ, ତାହା ସହ ମିଳାଇବା<br><br>୩. ଯାଞ୍ଚ କରିବା କି ନିକଟରେ କ୍ରୟ ହୋଇଥିବା ଜିନିଷକୁ ପୂର୍ବରୁ କ୍ରୟ ହୋଇଥିବା ଜିନିଷ ପଛରେ କ୍ରମାନ୍ୱୟରେ ଜମା କରାଯାଇଛି<br><br>୪. ଜିନିଷ ଗୁଡିକ ଏହାର ବିପଦ ଶ୍ରେଣୀ ମୁତାବକ ଜମା ହୋଇଛି କି ଉଦାହରଣ ସ୍ୱରୂପ<br><br>କ. ଆଲକୋହଲ କୁ ଅନ୍ୟତ୍ର ଲକ୍ କରି ଜମା ହୋଇଛି<br><br>ଖ. ଏସିଡ୍ ଗୁଡିକ ସର୍ବଦା ଅନ୍ୟତ୍ର ଚଟାଣ ଉପରେ ରଖା ହୋଇଛି	
5	8.	Use of positive and negative control for tests	Availability of Positive and negative control reagents<br><br>Check the use log of controls samples or reagents	

		ଟେଷ୍ଟ ଗୁଡିକ ପାଇଁ ପଜିଟିଭ୍ ଏବଂ ନେଗେଟିଭ୍ ନିୟନ୍ତ୍ରଣ ମାନଙ୍କ ବ୍ୟବହାର	ପଜିଟିଭ୍ ଏବଂ ନେଗେଟିଭ୍ ନିୟନ୍ତ୍ରଣ ମାନଙ୍କ ରୀଏଜେଣ୍ଟ୍ ର ଉପସ୍ଥିତି<br><br>ନିୟନ୍ତ୍ରଣ ନମୁନା କିମ୍ବା ରୀଏଜେଣ୍ଟ୍ ମାନଙ୍କ ବ୍ୟବହାର ଲଗ୍ ର ଯାଞ୍ଚ୍ କରିବା	
	9.	Labelling of equipment/instrument<br><br>ଉପକରଣ / ସରଞ୍ଜାମ କୁ ଚିହ୍ନିତ କରିବା	Respective unique I.D.s,<br>date of purchase,<br>date of installation,<br>date of putting into service,<br>date of the last calibration, and<br>Name and contact of address mechanic whom to inform in case of emergency<br><br>ସମ୍ବନ୍ଧୀତ ୟୁନିକ୍ ଚିହ୍ନଟ ସଂଖ୍ୟା,<br>କ୍ରୟ ତାରିଖ,<br>ଇନ୍-ସ୍ଟଲେସନ୍ ତାରିଖ,<br>କାର୍ଯ୍ୟକାରୀ ହେବାର ତାରିଖ,<br>ଶେଷ କ୍ୟାଲିବରେସନ ତାରିଖ ଏବଂ<br>ଅପାତକାଳୀନ ସ୍ଥିତିରେ ମେକାନିକ୍ ଙ୍କ ଯୋଗାଯୋଗ କରିବା, ନାମ ଏବଂ ଠିକଣା	
6	10.	Test requisition form<br><br>ପରୀକ୍ଷା ଅନୁରୋଧ ପତ୍ର	Test requisition form: Name, age, sex, Patient ID, Sample and Test required, Clinical diagnosis, Previous investigation result if any, Clinician Identity, Date, Box for Sampling date and time. Signature of recommending doctor, name of Ward/OPD/Clinic<br><br>ପରୀକ୍ଷା ଅନୁରୋଧ ପତ୍ର : ନାମ, ବୟସ, ଲିଙ୍ଗ, ରୋଗୀଙ୍କ ପରିଚୟ ସଂଖ୍ୟା, ନମୁନା ଏବଂ ଆବଶ୍ୟକ ପରୀକ୍ଷା, କ୍ଲିନିକାଲ୍ ଡାଇଗ୍ନୋସିସ୍, ପୂର୍ବ ଯାଞ୍ଚ ଫଳ ଯଦି କିଛି ଅଛି, କ୍ଲିନିସିଆନ୍ ଙ୍କ ପରିଚୟ, ତାରିଖ, ନମୁନା ସଂଗ୍ରହ ପାଇଁ ବକ୍ସର ତାରିଖ ଏବଂ ସମୟ, ପରାମର୍ଶ ଦେଇଥିବା ଡାକ୍ତରଙ୍କ ସ୍ୱାକ୍ଷର, ୱାର୍ଡ / ଓପିଡି / କ୍ଲିନିକ୍ ର ନାମ	
	11.	Sample Collection Instructions	To observe whether he/she gives the instructions to the patient (how to collect the sample) for example;<br>1. how to collect a urine sample for microbial culture from a male or a female patient?<br>2. How to collect urine sample for routine examination and for 24-hour urine protein analysis	

		 <br><br><br><br><br><br><br><br><br><br><br><br>ନମୁନା ସଂଗ୍ରହ ନିର୍ଦ୍ଦେଶ	3. How to collect throat sample from suspected cases of diphtheria.<br>4. ESR test : patient should be fasting<br>5. Coagulation studies: No hypertension medicine should be taken before sampling.<br>6. Iron Profile : No iron / folic acid intake prior to test<br><br>Instructions to patients to collect sputum sample from suspected pneumonia or Tuberculosis case<br><br>ସେ ରୋଗୀଙ୍କୁ ନିର୍ଦ୍ଦେଶ ଦେଉଛନ୍ତି କି (କିପରି ନମୁନା ସଂଗ୍ରହ କରିବେ) ଏହାର ଅନୁଧ୍ୟାନ କରିବେ ଉଦାହରଣ ସ୍ୱରୂପ;<br><br>୧. ମାଇକ୍ରୋବିଆଲ୍ କଲ୍ଚର୍ ପାଇଁ ପୁରୁଷ କିମ୍ବା ମହିଳା ରୋଗୀଙ୍କ ମୂତ୍ର ନମୁନା କିପରି ସଂଗ୍ରହ କରିବେ<br><br>୨. ରୁଟିନ୍ ପରୀକ୍ଷା ଏବଂ ୨୪ ଘଣ୍ଟିଆ ୟୁରିନ୍ ପ୍ରୋଟିନ୍ ଆନାଲିସିସ୍ ପାଇଁ ମୂତ୍ର ନମୁନା କିପରି ସଂଗ୍ରହ କରିବେ<br><br>୩. ଡିପ୍ଥେରିଆ ସନ୍ଦେହ ଥିବା କ୍ଷେତ୍ରରେ କିପରି ଗଳାରୁ ନମୁନା ସଂଗ୍ରହ କରିବେ<br><br>୪ . ଇ.ଏସ୍.ଆର୍ ପରୀକ୍ଷା : ରୋଗୀ ଉପବାସରେ ଥିବେ<br><br>୫ . କୋଆଗୁଲେସନ୍ ଅଧ୍ୟୟନ: ନମୁନା ପ୍ରସ୍ତୁତି ସମୟରେ କୌଣସି ଉଚ୍ଚ ରକ୍ତ ଚାପ ଔଷଧ ସେବନ କରି ନଥିବେ<br><br>୬ . ଆଇରନ୍ ପ୍ରୋଫାଇଲ୍ : ପରୀକ୍ଷା ପୂର୍ବରୁ ଆଇରନ୍ / ଫଲିକ୍ ଏସିଡ୍ ଗ୍ରହଣ ବର୍ଜିତ<br><br><br>ନିମୋନିଆ କିମ୍ବା ଯକ୍ଷ୍ମା ସନ୍ଦେହ ଥିବା ରୋଗୀଙ୍କର କଫ ନମୁନା ସଂଗ୍ରହ ପାଇଁ ନିର୍ଦ୍ଦେଶ	
7	12.	Specimen suitability<br><br><br><br><br>ନମୁନାର ଉପଯୁକ୍ତତା	Should explain at least one example like; Renal Function Test and Liver Function Tests require clear serum of the patient blood but if the blood sample is haemolysed it is not suitable for these tests to be performed.<br><br>ଅତି କମ୍ ରେ ଗୋଟିଏ ଉଦାହରଣ ଦେବେ ଯେପରି ରେନାଲ୍ ଫଙ୍କ୍ସନ୍ ପରୀକ୍ଷା ଏବଂ ଲିଭର୍ ଫଙ୍କ୍ସନ୍ ପରୀକ୍ଷା ପାଇଁ ରୋଗୀର ସ୍ୱଚ୍ଛ ସିରମ୍ ଆବଶ୍ୟକ କିନ୍ତୁ ଯଦି ରକ୍ତ ନମୁନା ହିମୋଲାଇଜ୍ ହୋଇଯାଏ ତେବେ ତାହା ଏହି ପରୀକ୍ଷା ଗୁଡିକ କରିବା ପାଇଁ ଉପଯୁକ୍ତ ରହେ ନାହିଁ	

8	13.	Manual Counting of Blood cells<br><br>ରକ୍ତ କୋଷର ମାନୁଆଲ୍ ଗଣନା	To observe whether the professional is able to perform the manual blood cell counting like, TLC, DLC, Platelets count etc.<br><br>ରକ୍ତ କୋଷ ର ଗଣନା ଟି ଏଲ୍ ସି, ଡି ଏଲ୍ ସି, ପ୍ଲେଟ୍ଲେଟ୍ ଗଣନା ଭଳି ଭଳି କାର୍ଯ୍ୟ କର୍ମଚାରୀ କରିପାରୁଛନ୍ତି କି ଏହାର ଅନୁଧ୍ୟାନ କରିବା	
	14.	Manual Biochemistry Tests<br><br>ମାନୁଆଲ୍ ବାୟୋକେମିଷ୍ଟ୍ରି ପରୀକ୍ଷା	To observe whether the professional is able to perform manually the common biochemistry tests example-Blood Sugar, Urea, Creatinine, electrolytes, Urine sugar, urine proteins, pregnancy tests etc.<br><br>କର୍ମଚାରୀ ସାଧାରଣ ବାୟୋକେମିଷ୍ଟ୍ରି ପରୀକ୍ଷା ଉଦାହରଣ ସ୍ୱରୂପ ରକ୍ତ ଶର୍କରା , ୟୁରିଆ , କ୍ରିଏଟିନିନ୍, ଇଲେକ୍ଟ୍ରୋଲାଇଟ୍, ମୂତ୍ର ଶର୍କରା, ୟୁରିନ୍ ପ୍ରୋଟିନ୍, ଗର୍ଭ ଧାରଣ ପରୀକ୍ଷା ଇତ୍ୟାଦି କରିବାରେ ସମର୍ଥ ଅଟନ୍ତି ଏହାର ଅନୁଧ୍ୟାନ କରିବା	
	15.	Microscopic Analysis of Clinical samples for cell morphology and counting<br><br>କ୍ଲିନିକାଲ୍ ନମୁନାର ମର୍ଫୋଲୋଜୀ ଏବଂ ଗଣନାର ମାଇକ୍ରୋସ୍କୋପିକ୍ ଆନାଲିସିସ୍	To observe whether the professional is able to perform for various clinical samples for cell morphology and counting. Example- Urine and other body fluid microscopy<br><br>ବିଭିନ୍ନ କ୍ଲିନିକାଲ୍ ନମୁନାର ମର୍ଫୋଲୋଜୀ ଏବଂ ଗଣନା ଉଦାହରଣ ସ୍ୱରୂପ ମୂତ୍ର ଏବଂ ଅନ୍ୟ ଶାରୀରିକ ତରଳର ମାଇକ୍ରୋସ୍କୋପି କରିବାରେ କର୍ମଚାରୀ ସମର୍ଥ ଅଟନ୍ତି ଏହାର ଅନୁଧ୍ୟାନ କରିବା	
	16.	Reference Values<br><br>ରେଫେରେନ୍ସ ଭାଲ୍ୟୁ	To observe whether reference values of each test results have been displayed in the laboratory or on the test requisition form<br><br>ଲାବୋରେଟୋରୀ କିମ୍ବା ପରୀକ୍ଷା ରିକ୍ୱିଜିସନ୍ ପତ୍ରରେ ରେଫେରେନ୍ସ ଭାଲ୍ୟୁ ଉଲ୍ଲେଖ ହୋଇଛି ଏହାର ଅନୁଧ୍ୟାନ କରିବା	
9	17.	Result Recording and Reporting<br><br>ରେଜଲ୍ଟ ରେକର୍ଡିଂ ଏବଂ ରିପୋର୍ଟିଂ	Observe the recording of results (Rough and final) - Sample Identity (Transcript Check)<br><br>ରେଜଲ୍ଟ ରେକର୍ଡିଂ(ରଫ୍ ଏବଂ ଫାଇନାଲ୍) – ନମୁନାର ପରିଚୟ (ଟ୍ରାନ୍ସକ୍ରୀପ୍ଟ ଚେକ୍) କୁ ଅନୁଧ୍ୟାନ କରିବା	
	18.	Positive and negative control Results to be matched with their known values<br><br>ପଜିଟିଭ୍ ଏବଂ ନେଗେଟିଭ୍ ନିୟନ୍ତ୍ରଣର ଜ୍ଞାତ ମୂଲ୍ୟ ସହ ମେଳନ	Observation – Before final report recording, does professional match the positive and negative control values with their actual and known values respectively.<br><br>ଅନୁଧ୍ୟାନ – କର୍ମଚାରୀ ଫାଇନାଲ୍ ରିପୋର୍ଟ ରେକର୍ଡିଂ ପୂର୍ବରୁ ପଜିଟିଭ୍ ଏବଂ ନେଗେଟିଭ୍ ନିୟନ୍ତ୍ରଣ ର ମୂଲ୍ୟକୁ ଏହାର ଜ୍ଞାତ ମୂଲ୍ୟ ସହ ମିଳାନ୍ତି କି	
	19.	Laboratory data Safety in reference to notifiable diseases like HIV, Tuberculosis etc.	Observe the accessibility to the laboratory records by any un-authorized persons.<br><br>Records kept under lock.	

		 <br><br><br><br>ନୋଟିଫାଏବଲ୍ ଡିଜିଜ୍ ଯେପରି ଏଚ.ଆଇ.ଭି, ଜକ୍ଷ୍ମା ଇତ୍ୟାଦି ସନ୍ଧର୍ଭରେ ଲାବୋରେଟୋରୀ ଡାଟା ଶୀଟ୍	Maintains the charge handover takeover records, while during shift change or while leaving for or joining back from leave or holidays.<br><br>କୌଣସି ଅନଧିକୃତ ବ୍ୟକ୍ତିଙ୍କ ଦ୍ୱାରା ଲାବୋରେଟୋରୀ ରେକର୍ଡକୁ ଆକ୍ସେସ୍ ର ଅନୁଧାନ<br>ରେକର୍ଡ୍ ଗୁଡିକ ଲକ୍ ରଖିବା| ପାଳି ପରିବର୍ତ୍ତନ , ଅବକାଶ ସମୟ କିମ୍ବା ଛୁଟି ପରେ କାର୍ଯ୍ୟରେ ପୁନଃ ଯୋଗ ଦେବା ସମୟରେ ରେକର୍ଡ୍ ଗୁଡିକର ହସ୍ତାନ୍ତରଣ ବଜାୟ ରଖିବା	
10	20.	General Safety precautions in medical laboratory<br><br><br><br><br><br><br><br><br><br><br><br>ମେଡିକାଲ ଲାବୋରଟୋରୀରେ ସାଧାରଣ ସୁରକ୍ଷା ସତର୍କତା	To demonstrate dealing with major and minor blood spills in and outside the equipment.<br><br>Observe his/ her laboratory for fire safety protocol which has been validated within year.<br><br>Observe the documentation related to fire safety training/ mock drill.<br><br>Observe for any the naked electrical connection /wire<br><br>ଉପକରଣ ଭିତରେ ଏବଂ ବାହାରେ ରକ୍ତ ଢାଳିଯିବା ପରିସ୍ଥିତି ସମ୍ଭାଳିବାର ପ୍ରଦର୍ଶନ<br>ତାଙ୍କ ଲାବୋରଟୋରୀରେ ଗତ ଏକ ବର୍ଷ ମଧ୍ୟରେ ସତ୍ୟାପିତ ହୋଇଥିବା ଅଗ୍ନି ସୁରକ୍ଷା ପ୍ରୋଟୋକଲ୍ ର ଅନୁଧାନ କରିବା<br>ଅଗ୍ନି ସୁରକ୍ଷା ପ୍ରଶିକ୍ଷଣ/ ମକ୍ ଡ୍ରିଲ୍ ସମ୍ବନ୍ଧୀୟ ଅଭିଲେଖର ଅନୁଧାନ କରିବା<br>କୌଣସି ନଗ୍ନ ବୈଦ୍ୟୁତିକ ସଂଯୋଗ/ ତାରର ଅନୁଧାନ କରିବା	
	21.	Laboratory Safety<br><br><br>ଲାବୋରେଟୋରୀ ସୁରକ୍ଷା	To observe what type of pipetting devices are being used in the laboratory.<br><br>ଲାବୋରେଟୋରୀରେ କେଉଁ ପ୍ରକାରର ପାଇପେଟିଂ ଯନ୍ତ୍ର ବ୍ୟବହାର ହେଉଛି ଏହାର ଅନୁଧାନ	
	22.	Chemical Safety<br><br><br><br><br><br>ରାସାୟନିକ ସୁରକ୍ଷା	To observe if any MSDS has been recorded in the laboratory documentation.<br><br>Observe that the chemicals have been kept following their hazardous category.<br><br>ଲାବୋରେଟୋରୀ ଅଭିଲେଖରେ କୌଣସି ଏମ୍.ଏସ୍.ଡି.ଏସ୍ ରେକର୍ଡ୍ ହୋଇଛି କି ଏହାର ଅନୁଧାନ<br>ରସାୟନ ଗୁଡିକ ତାଙ୍କ ବିପଦ ଶ୍ରେଣୀ ମୁତାବକ ରଖା ଯାଇଛି ଏହାର ଅନୁଧାନ କରିବା	

	23.	laboratory hygiene and infection control practices / Policy<br><br>ଲାବୋରେଟୋରୀ ପରିମଳ ଏବଂ ସଙ୍କ୍ରମଣ ନିୟନ୍ତ୍ରଣ ଅଭ୍ୟାସ/ ନିୟମ	Whether the laboratory has any written / documented and displayed infection control policy.<br><br>If yes, does the professional follow that policy?<br><br>ଲାବୋରେଟୋରୀ ର କୌଣସି ଲିଖିତ/ ପ୍ରଲେଖିତ ଏବଂ ପ୍ରଦର୍ଶିତ ସଙ୍କ୍ରମଣ ନିୟନ୍ତ୍ରଣ ନିୟମ ଅଛି କି<br>ଯଦି ହଁ , ତେବେ କର୍ମଚାରୀ ଏହି ନିୟମର ଅନୁପାଳନ କରନ୍ତି କି ?	
	24.	Disposal of used Syringes and needles<br><br>ବ୍ୟବହାର ହୋଇଥିବା ସିରିଞ୍ଜ ଏବଂ ନିଡିଲ୍ ର ଡିସପୋଜାଲ୍	To observe how does the needles and syringes are being disposed of?<br><br>Whether the laboratory has dedicated functional syringe needle destroyer<br><br>What type of syringe needle destroyer you have: burning type and mechanical cutting based?<br><br>ଅନୁଧ୍ୟାନ କରିବା କି ସିରିଞ୍ଜ ଏବଂ ନିଡିଲ୍ କିପରି ଡିସପୋଜ୍ ହେଉଛି ?<br>ଲାବୋରେଟୋରୀରେ ଡେଡିକେଟେଡ୍ ସିରିଞ୍ଜ ଏବଂ ନିଡିଲ୍ ନଷ୍ଟ ଯନ୍ତ୍ର ଅଛି କି<br>କେଉଁ ପ୍ରକାରର ସିରିଞ୍ଜ ଏବଂ ନିଡିଲ୍ ନଷ୍ଟ ଯନ୍ତ୍ର ଆପଣଙ୍କ ନିକଟରେ ଉପଲବ୍ଧ : ବର୍ଣ୍ଣିଙ୍ଗ ଟାଇପ୍ ଏବଂ ମେକାନିକାଲ୍ କଟିଙ୍ଗ ଆଧାରିତ?	
	25.	Hand hygiene<br><br>ହସ୍ତ ପରିମଳ	To observe whether the professional follows the proper hand hygiene protocol as per WHO Protocol.<br><br>କର୍ମଚାରୀ ଡବ୍ଲୁ.ଏଚ.ଓ ପ୍ରୋଟୋକଲ୍ ଅନୁସାରେ ସଠିକ୍ ହ୍ୟାଣ୍ଡ ହାଇଜିନ୍ ପ୍ରୋଟୋକାଲ୍ ପାଳନ କରନ୍ତି କି ଏହାର ଅନୁଧ୍ୟାନ|	
	26.	Sharp Cuts<br><br>ଧାର ଜନିତ କଟା କ୍ଷତ	To observe the register/ documented record in which sharp cuts are being recorded.<br><br>ରେଜିଷ୍ଟର୍ / ଅଭିଲେଖ ରେକର୍ଡ୍ ଯେଉଁଥିରେ ଧାର ଜନିତ କଟା କ୍ଷତ ରେକର୍ଡ୍ କରାଯାଇଥିବା ଏହାର ଅନୁଧ୍ୟାନ	

C.3. Mini Clinical Laboratory Skills
ଗ୩. ମିନି ଲାବୋରେଟୋରୀ କୌଶଳ

Score Definition
ମୂଲ୍ୟାଙ୍କନର ପରିଭାଷା

A. None—No demonstrated skills at all/does not perform the task(s) completely
କ. ନାହିଁ - କୌଣସି କୌଶଳ ଉପସ୍ଥିତ ନାହିଁ/ କାର୍ଯ୍ୟ ସମ୍ପୂର୍ଣ୍ଣ କରି ନାହାନ୍ତି|

B. Limited Demonstrated very limited strengths/skills in this area
ଖ. ଏହି କ୍ଷେତ୍ରରେ ବହୁତ କମ ମାତ୍ରାରେ ସାମର୍ଥ୍ୟ / କୌଶଳର ପ୍ରଦର୍ଶନ|
C. Some—Demonstrated some ability/skills in this area.
ଗ. ସ୍ୱଳ୍ପ- ଏହି କ୍ଷେତ୍ରରେ ସ୍ୱଳ୍ପ ସାମର୍ଥ୍ୟ/ କୌଶଳର ପ୍ରଦର୍ଶନ|
D. Strong—Demonstrated strong skills/strength in this area.
ଘ. ଦୃଢ - ଏହି କ୍ଷେତ୍ରରେ ଦୃଢ ସାମର୍ଥ୍ୟ/ କୌଶଳର ପ୍ରଦର୍ଶନ|
E. Excellent—Demonstrated excellent skills/strength in this area.
ଙ. ଅସାଧାରଣ - ଏହି କ୍ଷେତ୍ରରେ ଅସାଧାରଣ ସାମର୍ଥ୍ୟ/ କୌଶଳର ପ୍ରଦର୍ଶନ|
F. Not applicable.
ଚ. ଆବଶ୍ୟକତା ନାହିଁ|
G. Don’t know- Not even heard about that skill.
ଛ. ଜାଣି ନାହାନ୍ତି - କୌଶଳ ବିଷୟରେ ଅନଭିଜ୍ଞ|
H. Skill limitation is clearly related to resource limitations.
D. ଜ. ସୀମିତ ସମ୍ବଳ କାରଣରୁ ସୀମିତ କୌଶଳର ପ୍ରଦର୍ଶନ ସ୍ପଷ୍ଟ ପରିଲକ୍ଷିତ|

Dom. ଡୋମେନ୍ କୋଡ୍	S.N. କ୍ରମିକ ସଂଖ୍ୟା	Clinical Evaluation Points କ୍ଲିନିକାଲ ଆକଳନ ବିନ୍ଦୁ	Means of Verification ସତ୍ୟାପନର ସାଧନ	Response (Score) ପ୍ରତିକ୍ରିୟା (ଅଙ୍କ)	Remarks ଟିପ୍ପଣୀ
1	1	Demonstration of Sample collection tools<br><br>ନମୁନା ସଂଗ୍ରହ ଉପକରଣ ଗୁଡିକର ପ୍ରଦର୍ଶନ	Whether he/she is able to demonstrate sample collection tool like; sterile syringe and needle, lancet, cotton /rayon swab sticks, suitable anticoagulants, and sample collection containers for the samples, which are routinely processed.<br><br>ଯଦିବା ସେ ଦୈନନ୍ଦିନ କାର୍ଯ୍ୟହୃତ ନମୁନା ସଂଗ୍ରହ ଉପକରଣ ଯେପରି ଷ୍ଟେରାଇଲ୍ ସିରିଞ୍ଜି ଏବଂ ନିଡିଲ୍ ଲାନ୍ସେଟ୍, କଟନ୍/ ରେଅନ୍ ସ୍ୱାବ୍ ଷ୍ଟିକ୍, ଉପଯୁକ୍ତ ଆଣ୍ଟିକୋଆଗୁଲାଣ୍ଟ ଏବଂ ନମୁନା ସଂଗ୍ରହ ବକ୍ସ୍ ଗୁଡିକୁ ପ୍ରଦର୍ଶିତ କରିବାରେ ସମର୍ଥ		
	2	Use of anticoagulants for specific tests	Proper and suitable coagulants in sample collection vials as per the test requirement. For example; requirement of serum, plasma and whole blood-for hemogram is done in EDTA (Ethylenediaminetetraacetic acid) Containing sample vials; and for blood sugar estimation, sample should be collected in sodium fluoride containing vial, and the general biochemistry test, no anticoagulants should be in the vial.		

		ନିର୍ଦ୍ଦିଷ୍ଟ ପରୀକ୍ଷା ପାଇଁ ଆଣ୍ଟିକୋଆଗୁଲାଣ୍ଟ୍ ର ବ୍ୟବହାର	ନମୁନା ସଂଗ୍ରହ ଭାଇଲ୍ ମଧ୍ୟରେ ପରୀକ୍ଷାର ଆବଶ୍ୟକତା ଅନୁସାରେ ସଠିକ୍ ଏବଂ ଉପଯୁକ୍ତ କୋଆଗୁଲାଣ୍ଟର ଉପସ୍ଥିତି ଉଦାହରଣ ସ୍ୱରୂପ ସେରମ୍, ପ୍ଲାଜ୍ମା ଏବଂ ହୋଲ ବ୍ଲଡ୍ ହିମୋଗ୍ରାମ ଇ.ଡି.ଟି.ଏ (ଇଥାଇଲଡାଇଆମାଇନଟେଟ୍ରାଏସିଟିକ୍ ଏସିଡ୍) ଥିବା ନମୁନା ଭାଇଲ୍ ର ଆବଶ୍ୟକତା ଏବଂ ରକ୍ତ ଶର୍କରା ଆକଳନ କରିବା ପାଇଁ ସୋଡ଼ିୟମ୍ ଫ୍ଲୋରାଇଡ୍ ଥିବା ଭାଇଲ୍ ଏବଂ ସାଧାରଣ ବାୟୋକେମିଷ୍ଟ୍ରି ପରୀକ୍ଷା ପାଇଁ ଭାଇଲ୍ ମଧ୍ୟରେ କୌଣସି ଆଣ୍ଟିକୋଆଗୁଲାଣ୍ଟ୍ ର ଆବଶ୍ୟକତା ନାହିଁ		
	3	Staining Procedures<br><br>ଷ୍ଟେନିଂ ପ୍ରକ୍ରିୟା	Able to demonstrate the principles, reagents used and procedures of various staining methods used in his/her laboratory.<br>Example 1. Preparation of thin and thick smear of blood for detection of malarial parasite.<br><br>Example 2. Various staining procedures available in the lab for detection of malarial parasite.<br><br>Example 3. Preparation of blood film on glass slide for differential leucocyte count (DLC) and cell morphology.<br><br>ତାଙ୍କ ଲାବୋରେଟୋରୀରେ ବିଭିନ୍ନ ଷ୍ଟେନିଂ ପଦ୍ଧତିରେ ବ୍ୟବହୃତ ସିଦ୍ଧାନ୍ତ , ରିଏଜେଣ୍ଟ୍ ଏବଂ ପ୍ରକ୍ରିୟାକୁ ପ୍ରଦର୍ଶିତ କରିବାରେ ସମର୍ଥ ଅଟନ୍ତି<br>ଉଦାହରଣ ୧ ମ୍ୟାଲେରିଆ ପାରାସାଇଟ୍ ଚିହ୍ନଟ ପାଇଁ ଥିନ୍ ଏବଂ ଥିକ୍ ସ୍ମିଅର୍ ପ୍ରସ୍ତୁତି<br>ଉଦାହରଣ ୨ ମ୍ୟାଲେରିଆ ପାରାସାଇଟ୍ ଚିହ୍ନଟ ପାଇଁ ଉପଲବ୍ଧ ବିଭିନ୍ନ ଷ୍ଟେନିଂ ପ୍ରକ୍ରିୟା ଉଦାହରଣ<br>୩ ଡିଫରେନ୍ସିଆଲ୍ ଲ୍ୟୁକୋସାଇଟ୍ ଗଣନା (ଡି.ଏଲ୍.ସି) ଏବଂ କୋଷ ମର୍ଫୋଲୋଜି ପାଇଁ ରକ୍ତ ଫିଲ୍ମ ପ୍ରସ୍ତୁତ କରିବା		
	4	Microscopy	Should be able to focus the smear efficiently under the microscope and observe for abnormalities therein in relation to the expectation of the test. For example- can he find malarial parasite present in the blood film under the microscope.		

		ମାଇକ୍ରୋସ୍କୋପି	ରକ୍ତ ସ୍ମିଅର୍ କୁ ମାଇକ୍ରୋସ୍କୋପ୍ ତଳେ ଫୋକସ୍ କରିବା ଏବଂ ପରୀକ୍ଷାର ଆଶାତୀତ ପରିଣାମ ଅନୁଯାଇ ଅସମାନ୍ୟତାର ଅନୁଧ୍ୟାନ କରିବାରେ ସମର୍ଥ ଅଟନ୍ତି ଉଦାହରଣ ସ୍ୱରୂପ କ'ଣ ସେ ରକ୍ତ ଫିଲ୍ମରେ ଥିବା ମ୍ୟାଲେରିଆ ପାରାସାଇଟ୍ କୁ ମାଇକ୍ରୋସ୍କୋପ୍ ରେ ଚିହ୍ନଟ କରିପାରୁଛନ୍ତି|		

C.4. Case Study
ଗ୪. କେସ୍ ଷ୍ଟଡି

Dom<br>ଡୋମେନ୍ କୋଡ୍	Case<br>କେସ୍	Response (Satisfactory/ Unsatisfactory)<br>ପ୍ରତିକ୍ରିୟା (ସନ୍ତୋଷଜନକ /ଅସନ୍ତୋଷଜନକ)	Remarks<br>ଟିପ୍ପଣୀ
1	**Able to organize work to accommodate valid priorities- for example-**<br>Last month, one patient Rima landed your hospital with gestational complications. Medical Officer of your facility identified this as critical case that needs immediate lower abdomen Caesarean section. The doctor on duty sent her blood sample to your laboratory for D-dimer. At the same time, a patient comes to doctor as an emergency case. Symptomatically, doctor wishes to rule out the diabetes and sends the sample to laboratory for blood sugar testing. Under the circumstances, to which sample you will prioritize to process.		1. Hemoglobin, WBCs and platelets estimation prior to Radiotherapy, chemotherapy<br>2. Platelet counts in suspected dengue cases<br>3. Coagulation studies in case of poising may be snake bite<br>4. RFT before MRI/CT scan where contrast is being used.<br>5. PTI in treatment: Healthcare providers often do this test to monitor your prothrombin levels if you're taking the blood thinner warfarin (Coumadin®). Warfarin helps prevent blood clots, which can cause serious conditions such as deep venous thrombosis or pulmonary embolism.

	**ପ୍ରାଥମିକତା ନିଶ୍ଚିତ କରିବା ପାଇଁ କାର୍ଯ୍ୟକୁ ବ୍ୟବସ୍ଥିତ କରିବାରେ ସମର୍ଥ ଉଦାହରଣ ସ୍ବରୂପ-** ଗତ ମାସରେ ରିମା ନାମକ ଜଣେ ରୋଗୀ ଜେଷ୍ଟେସ୍ନାଲ୍ କମ୍ପ୍ଲିକେସନ୍ସ ସହିତ ଆପଣଙ୍କ ହସ୍ପିଟାଲ କୁ ଆସନ୍ତି ଆପଣଙ୍କ କେନ୍ଦ୍ର ମେଡିକାଲ୍ ଅଫିସର୍ ଏହାକୁ କ୍ରିଟିକାଲ୍ କେସ୍ ରୂପେ ଚିହ୍ନଟ କରନ୍ତି ଯାହାକୁ ତୁରନ୍ତ ଲୋଅର୍ ଆବ୍ଡୋମେନ୍ ସିଜରିଆନ୍ ସେକ୍ସନ୍ ର ଆବଶ୍ୟକତା ଅଛି କାର୍ଯ୍ୟରତ ଡାକ୍ତର ତାଙ୍କର ଡି-ଡାଇମର୍ ପାଇଁ ରକ୍ତ ନମୁନାକୁ ଆପଣଙ୍କ ଲାବୋରେଟୋରୀକୁ ପଠାନ୍ତି ଠିକ୍ ସେହି ସମୟରେ ଆଉ ଜଣେ ରୋଗୀ ଦୁର୍ବଳତା ଏବଂ ଗୋଡ଼ରେ ଯନ୍ତ୍ରଣାର ଅସୁବିଧା ସହ ଡାକ୍ତରଙ୍କ ପାଖକୁ ଆସନ୍ତି ଡାକ୍ତର ମଧୁମେହର ସନ୍ଦେହ ଦୂର ପାଇଁ ରକ୍ତ ଶର୍କରା ପରୀକ୍ଷା ପାଇଁ ନମୁନାକୁ ଲାବୋରେଟୋରୀ ପଠାନ୍ତି ଏହି ପରିସ୍ଥିତିରେ ଆପଣ କେଉଁ ନମୁନାକୁ ପରୀକ୍ଷା ପାଇଁ ପ୍ରାଥମିକତା ଦେବେ		୧ . ରେଡିଓଥେରାପି, କେମୋଥେରାପି ପୂର୍ବରୁ ହିମୋଗ୍ଲୋବିନ୍ ଡବ୍ଲୁ.ବି.ସି ଏବଂ ପ୍ଲେଟ୍ଲେଟ୍ ର କଳନା<br>୨ . ସନ୍ଦିଗ୍ଧ ଡେଙ୍ଗୁ କେସ୍ ରେ ପ୍ଲେଟ୍ଲେଟ୍ ଗଣନା<br>୩ . ବିଷକ୍ରିୟା , ସର୍ପାଘାତ ହେଇଥିବା କ୍ଷେତ୍ରରେ କୋଆଗୁଲେସନ୍ ଅଧ୍ୟୟନ<br>୪ . ଏମ୍.ଆର୍.ଆଇ/ ସିଟି ସ୍କାନ୍ ଯେଉଁଠାରେ କଣ୍ଟ୍ରାସ୍ଟ ର ବ୍ୟବହାର ହୋଇଥାଏ ପୂର୍ବରୁ ଆର୍.ଏଫ୍.ଟି କରାଇବା<br>ଚିକିତ୍ସା ରେ ପି.ଟି.ଆଇ : ଯଦି ଆପଣ ରକ୍ତ ତରଳକାରି ବାଫାରିନ୍ (କୋମେଡେନ୍) ସେବନ କରୁଥାନ୍ତି ତେବେ ଆପଣଙ୍କ ପ୍ରଥ୍ରମ୍ବିନ୍ ସ୍ତର ଉପରେ ଦୃଷ୍ଟି ରଖିବା ପାଇଁ ସ୍ୱାସ୍ଥ୍ୟ ସେବା ପ୍ରଦାନକାରୀମାନେ ପ୍ରାୟତଃ ଏହି ପରୀକ୍ଷାଟି କରିଥାନ୍ତି. ବାଫାରିନ୍ ରକ୍ତ ଜମାଟ ରୋକିବାରେ ସାହାଯ୍ୟ କରେ ଯାହା ଗମ୍ଭୀର ପରିସ୍ଥିତି ଯେପରି ଭେନସ୍ ଥ୍ରୋମ୍ବୋସିସ୍ କିମ୍ବା ପଲ୍ମୋନାରୀ ଏମ୍ବୋଲିଜିମ୍ କରାଇପାରେ
2	Able to demonstrate effective problem solving/trouble-shooting strategies and initiates the appropriate follow-up **for example-**<br>Suppose you have received one sample from the physician who wishes to know whether or not the sample contains any gram positive or gram-negative microbe in it. Per chance you found that your gram stain kit is out of stock. On enquiring from the store, the storekeeper informed that except gram staining kit everything is available in the store. Under the circumstances, how would you be able to perform gram staining and help the clinician in making diagnosis of concerned patient?<br>2. The professional did Leishman stain on blood sample but the staining results are not proper. What may be the causes?<br>3. Deposition of deposits on smear after staining. What may be the reasons<br><br>By this case study, the assessor wishes to know whether the laboratory technician will do efforts to perform the test using some alternative method, if the standard method		As the store-keeper clarified that he has everything except gram staining kit, the professional should get the chemicals (Crystal Violet, Safranin, Acetone, and Alcohol) , issued from the store, which are required to prepare the gram staining reagents manually and perform the procedure.

	diagnostic kit or reagents are not available per chance.<br><br>ପ୍ରୋବ୍ଲେମ୍ ଶଲ୍ଭିଙ୍ଗ / ଟ୍ରବଲ୍ ଶୁଟିଂ ରଣନୀତି ଏବଂ ଉପଯୁକ୍ତ ଫଲୋଅପ୍ ଆରମ୍ଭ କରିବାରେ ସମର୍ଥ ଅଟନ୍ତି ଉଦାହରଣ ସ୍ୱରୂପ ଧରନ୍ତୁ ଆପଣ ଫିଜିସିଆନ୍ ଙ୍କଠୁ ଏକ ନମୁନା ପ୍ରାପ୍ତ କରନ୍ତି ଯିଏ ଜାଣିବାକୁ ଚାହାନ୍ତି କି ନମୁନାରେ ଗ୍ରାମ୍ ପଜିଟିଭ୍ କିମ୍ବା ଗ୍ରାମ୍ ନେଗେଟିଭ୍ ମାଇକ୍ରୋବ୍ ଉପସ୍ଥିତ ଅଛି କି ନାହିଁ, କିନ୍ତୁ ଆପଣ ଜାଣିବାକୁ ପାଇଲେ କି ଆପଣଙ୍କ ଗ୍ରାମ ଷ୍ଟେନ୍ କୀଟ୍ ସରି ଯାଇଛି| ଭଣ୍ଡାରରେ ପଚାରିବାରୁ ଭଣ୍ଡାର ରକ୍ଷକ ଜଣାନ୍ତି କି ଗ୍ରାମ୍ ଷ୍ଟେନ୍ କୀଟ୍ ବ୍ୟତୀତ ସମସ୍ତ ଜିନିଷ ଉପଲବ୍ଧ ଅଛି| ଏହି ପରିସ୍ଥିତିରେ ଆପଣ କିପରି ଗ୍ରାମ୍ ଷ୍ଟେନିଂ କରିବେ ଏବଂ ସମ୍ବନ୍ଧିତ ରୋଗୀର ଦଇଗନୋସିସ ଡାଇଗ୍ନୋସିସ୍ ରେ କ୍ଲିନିସିଆନ୍ ଙ୍କୁ ସାହାଯ୍ୟ କରିବେ<br>୨. କର୍ମଚାରୀ ରକ୍ତ ନମୁନା ଉପରେ ଲିସ୍ମାନ୍ ସ୍ଟେନ୍ କରିଥିଲେ କିନ୍ତୁ ଷ୍ଟେନିଂ ର ପରିଣାମ ସଠିକ୍ ନୁହନ୍ତି|କାରଣ ଗୁଡିକ କ'ଣ ହୋଇପାରେ?<br>୩. ଷ୍ଟେନିଂ ପରେ ସ୍ମୀଅର୍ ଉପରେ ପଙ୍କୁ ଜମିଜିବା| କାରଣ ଗୁଡିକ କ'ଣ ହୋଇପାରେ?<br><br>ଏହି କେସ୍ ଷ୍ଟଡି ଦ୍ୱାରା ମୁଲ୍ୟାୟନକାରୀ ଜାଣିବାକୁ ଚାହାନ୍ତି କି, ଯଦି ମାନକ ପ୍ରଣାଳୀ ଡାଇଗ୍ନୋସ୍ଟିକ୍ କୀଟ୍ କିମ୍ବା ରିଏଜେଣ୍ଟ୍ ଯୋଗ ବଶତଃ ଉପଲବ୍ଧ ନାହିଁ, ଲାବୋରେଟୋରୀ ଟେକ୍ନିସିଆନ୍ କିଛି ବିକଳ୍ପ ପ୍ରଣାଳୀ ବ୍ୟବହାର କରି ପରୀକ୍ଷାକୁ ପୁରା କରିବାକୁ ଚେଷ୍ଟା କରିବେ କି|		ଯେ ହେତୁ ଭଣ୍ଡାର ରକ୍ଷକ ଜଣାଇଛନ୍ତି କି ତାଙ୍କ ପାଖରେ ଗ୍ରାମ୍ ଷ୍ଟେନିଂ କୀଟ୍ ବ୍ୟତୀତ ସବୁ ଜିନିଷ ଉପଲବ୍ଧ ଅଛି ତେବେ କର୍ମଚାରୀ ଙ୍କୁ ପରୀକ୍ଷା କରିବା ପାଇଁ କେମିକାଲ ଗୁଡିକ (କ୍ରିଷ୍ଟାଲ୍ ଭାୟୋଲେଟ୍, ସାଫ୍ରାନିନ୍, ଏସିଟୋନ୍ ଏବଂ ଆଲ୍କହଲ) ଷ୍ଟୋର୍ ରୁ ଯୋଗାଣ କରିବାକୁ ହେବ ଯାହାକି ମାନୁଆଲି ଗ୍ରାମ୍ ଷ୍ଟେନିଂ ରିଏଜେଣ୍ଟ୍ ପ୍ରସ୍ତୁତ କରିବା ପାଇଁ ଆବଶ୍ୟକ
3	Risk assessment for any processes or in using instruments/ equipment **for example-**<br><br>Suppose you wish to centrifuge the blood samples to separate serum. What type of risks, may be there, which need to be assessed so that centrifugation is expected to be done as risk free?		He will assess that -1. the machine is properly working<br>2. There is nothing unrequired inside the chamber like; broken glass, any type of spilled fluid etc.<br>3. The shock absorbing cushions are present in all the test tube cups.<br>4. The test tubes and cups are properly balanced.<br>5. Ensure that the lid is properly locked, before starting the machine.<br>6. Increases the speed slowly not with the sudden jerk.

	କୌଣସି ଉପକରଣ / ସରଞ୍ଜାମ ବ୍ୟବହାର କରିବାରେ ବିପଦ ଆକଳନ କରିବା **ଉଦାହରଣ ସ୍ୱରୂପ-**<br>ଧରନ୍ତୁ ଆପଣ ରକ୍ତ ନମୁନାରୁ ସିରମ୍ ଅଲଗା କରିବା ପାଇଁ ସେଣ୍ଟ୍ରୀଫ୍ୟୁଜ୍ ବ୍ୟବହାର କରିବାକୁ ଚାହାନ୍ତି ତେଣୁ ଏଥିରେ କେଉଁ ଧରଣର ବିପଦ ଥାଇପାରେ ଯାହାର ଆକଳନ ସେଣ୍ଟ୍ରୀଫ୍ୟୁଜ୍ ପ୍ରକ୍ରିୟା ଆଶାତୀତ ରୂପେ ବିପଦ ମୁକ୍ତ ରହି କରିବା ପାଇଁ ଆବଶ୍ୟକ?		7. After completion of centrifugation time and switching it off he waits till the rotor stops automatically.<br>8. Whether he waits minimum for 5-10 Minutes allowing the aerosols to settle if any before opening the lid.<br><br>ସେ ଆକଳନ କରିବେ କି<br>୧. ଉପକରଣ ଠିକ୍ କାମ କରୁଛି<br>୨. ଚ୍ୟାମ୍ବର୍ ମଧ୍ୟରେ କୌଣସି ଅଦରକାରୀ ପଦାର୍ଥ ଯେପରି ଭଙ୍ଗା କାଚ, କୌଣସି ପ୍ରକାରର ତରଳ ଇତ୍ୟାଦି ନଥିବ।<br>୩. ସମସ୍ତ ଟେଷ୍ଟ ଟ୍ୟୁବ୍ କପ୍ ରେ ଶକ୍ ଅବ୍ସରବିଙ୍ଗ୍ କୁଶନ୍ ମହଜୁଦ ଅଛି।<br>୪. ଟେଷ୍ଟ ଟ୍ୟୁବ୍ ଏବଂ କପ୍ ଗୁଡିକ ସଠିକ୍ ସନ୍ତୁଳନରେ ଥିବେ।<br>୫. ସୁନିଶ୍ଚିତ କରିବେକି ଉପକରଣ ଆରମ୍ଭ କରିବା ପୂର୍ବରୁ ଢାଙ୍କୁଣୀ ଠିକ୍ ଭାବେ ବନ୍ଦ ଥିବ।<br>୬. ଗତିକୁ ହଠାତ୍ ନ ବଢ଼ାଇ ଧୀରେ ଧୀରେ ବଢାଇବା।<br>୭. ସେଣ୍ଟ୍ରୀଫ୍ୟୁଜ୍ ସରିବା ପରେ ଉପକରଣ ବନ୍ଦ କରିବା ପୂର୍ବରୁ ରୋଟର୍ ସ୍ୱତଃ ସ୍ଥିରାବସ୍ଥା ପାଇଁ ଅପେକ୍ଷା କରିବା।<br>୮. କ'ଣ ସେ ଢାଙ୍କୁଣୀ ଖୋଲିବା ପୂର୍ବରୁ ଏରୋସୋଲ୍ ଗୁଡିକୁ ବସିଜିବା ପାଇଁ ୫- ୧୦ ମିନଟ ଅପେକ୍ଷା କରୁଛନ୍ତି ।
4	Critical Alerts:<br>**for example-**<br>A sixty-year-old patient approaches the emergency with some problems and symptomatically the physician expects it as a hypoglycemic case and sends his blood sample for blood sugar testing. You performed the test and found that his blood glucose level is 40 mg/dl. What will your further action?<br><br>ମହତ୍ୱପୂର୍ଣ୍ଣ ଚେତାବନୀ<br>**ଉଦାହରଣ ସ୍ୱରୂପ-**<br>ଜଣେ ୬୦ ବର୍ଷ ବୟସ୍କ ରୋଗୀ କିଛି ଅସୁବିଧା ସହ ଅପାତକାଳୀନ ସେବା କୁ ଆସନ୍ତି ଏବଂ ଫିଜିସିଆନ୍ ଏହାକୁ ହାଇପୋଗ୍ଲିସେମିକ୍ କେସ୍ ସନ୍ଦେହ କରନ୍ତି ଏବଂ ରକ୍ତ ନମୁନାକୁ ରକ୍ତ ଶର୍କରା ପରୀକ୍ଷା		Since the blood glucose level of the patient is very low and patient may enter into coma phase, considering it critical the laboratory professional should convey the report immediately to the physician, as a critical alert so that the patient may be saved.<br><br>ରୋଗୀଙ୍କର ଯେ ହେତୁ ରକ୍ତ ଶର୍କରା ସ୍ତର ବହୁତ କାମ ଅଛି, ରୋଗୀ କୋମା ଅବସ୍ଥାକୁ ଯାଇପାରନ୍ତି। ଏହାକୁ ବିପଦ୍ଜନକ ବିଚାର କରି ଲାବୋରେଟୋରୀ କର୍ମଚାରୀ ରିପୋର୍ଟକୁ ଯଥା ଶୀଘ୍ର ମହତ୍ୱପୂର୍ଣ୍ଣ ଚେତାବନୀ

	ପାଇଁ ପଠାନ୍ତି | ଆପଣ ପରୀକ୍ଷା କରନ୍ତି ଏବଂ ପାଆନ୍ତିକି ତାଙ୍କ ରକ୍ତରେ ଶର୍କରା ସ୍ତର ୪୦ ଏମ୍.ଜି/ ଡି.ଏଲ ଅଟେ। ଆପଣଙ୍କ ପରବର୍ତ୍ତୀ ପଦକ୍ଷେପ କ'ଣ ରହିବ ?		ହିସାବରେ ଫିଜିସିଆନ୍ ଙ୍କୁ ଅବଗତ କରାଇବା ଉଚିତ ଯାହା ଫଳରେ ରୋଗୀଙ୍କ ଜୀବନ ବଞ୍ଚାଇ ହେବ।

*************************************The End***
