# Supplementary material for: Developing a competency assessment framework for medical laboratory technologists in primary healthcare settings in India
Source: PLoS One. 2024 Apr 1;19(4):e0294939. doi: 10.1371/journal.pone.0294939 (PMC10984544; doi:10.1371/journal.pone.0294939)
Supplement: Attachment — Submitted filename: Rebuttal Letter.pdf [file pone.0294939.s006.pdf]

Sr. No.	Editor/Reviewer Comments	Authors Response	Page No and Line Number
01	Please consider the comments made by the reviewers. It may not be easy to discuss the experience of implementation of the framework if it has not yet been implemented, but please comment based on any experience that is available.	Thank you for your suggestions/comments. We have made the necessary changes.	
02	Results and Discussion should include examples on objections or matters of concern raised against the competencies that have been proposed but did not reach unanimous consensus.	Thank you for your comments. We have made the necessary changes in the results.	Page 20 and Line number 408 to 411
03	Is there record regarding the (average) time spent on completing various parts of the CAT listed in Additional file 3, such as time spent on each questionnaire and each observational assessment? How many assessors were assigned on Observational Tools (Skills) and Mini Clinical Laboratory Skills? How are the case studies conducted, verbally or written?	Thank you. We have addressed your comments.	Page 23 and Line number 448 to 457
04	While such professional assessment is usually conducted by nominees working on voluntary basis, the exercise will still incur financial commitments. Please suggest plans for such financial support.	Thank you. We have addressed your comments.	Page 26, Line No. 523 to 526
05	What is the plan of implementing the CAT studied and will it become mandatory for MLT license or practise? Please also recommend if such assessment will be conducted regularly and the frequency if applicable.	Thank you for the valid question. Policymakers have yet to make this policy decision to implement this framework.	
06	In addition to the limitations discussed near the end of the manuscript, are there other hurdles that the authors anticipate in implementing CAT established in this exercise? For instance, will the diversity of local languages be a problem? The CAT needs to be improvised when the bilingual version is finalized as described under Stage 5 Pre-testing.	Thank you. We have addressed your comments.	Page 25 and Line Number 505 to 507
